# Bacterial Nanocellulose Functionalization for Smart Bioelectronics: Integration into Biosensing, Neural Interfaces, and Tissue Engineering

**DOI:** 10.1021/acspolymersau.5c00074

**Published:** 2025-10-03

**Authors:** Maurelio Cabo, Farbod Ebrahimi, Jeffrey R. Alston, Rutujaa Kulkarni, Samir Kattel, Kristen Dellinger, Dennis LaJeunesse

**Affiliations:** † Department of Nanoscience, Joint School of Nanoscience and Nanoengineering, 14616 The University of North Carolina at Greensboro, Greensboro, North Carolina 27401, United States; ‡ Department of Nanoengineering, Joint School of Nanoscience and Nanoengineering, North Carolina A&T University, Greensboro, North Carolina 27401, United States; § Department of Applied Science and Technology, 3616 North Carolina Agricultural and Technical State University, Greensboro, North Carolina 27411, United States

**Keywords:** bacterial nanocellulose, smart bioelectronics, biosensors, tissue engineering, neural interfaces, nanocomposites, drug delivery systems, biomaterials, biomedical

## Abstract

Bacterial nanocellulose (BNC), a renewable biopolymer biosynthesized by specific bacterial strains, exhibits exceptional mechanical strength, water retention, and biocompatibility due to its nanofibrillar 3D architecture and high purity. Functionalizing BNC with conductive polymers, metal nanoparticles, enzymes, and peptides unlocks its potential for diverse applications in smart bioelectronics, including biosensors, neural interfaces, and tissue engineering. This review presents a comprehensive analysis of recent strategies for tuning BNC’s electrical, optical, biological, and mechanical properties to meet the evolving demands of next-generation biomedical and wearable devices. We discuss a broad range of functionalization methods—from in situ nanoparticle synthesis and electrostatic assembly to cross-linking and doping with ionic liquids—and explore their role in enhancing conductivity, stimuli-responsiveness, and cellular interactions. Furthermore, we examine BNC-based nanocomposites designed for biosensing, wound healing, optoelectronic sensing, and flexible implantable systems. The review concludes by outlining current key hurdles including scalability, device integration, long-term stability, and stringent regulatory requirements for safe production, use, and clinical translation, while uniquely positioning BNC through a cross-domain comparison of biomedical and electronic applications, complemented by techno-economic insights into scale-up, cost, and regulatory challenges.

## Graphical abstract

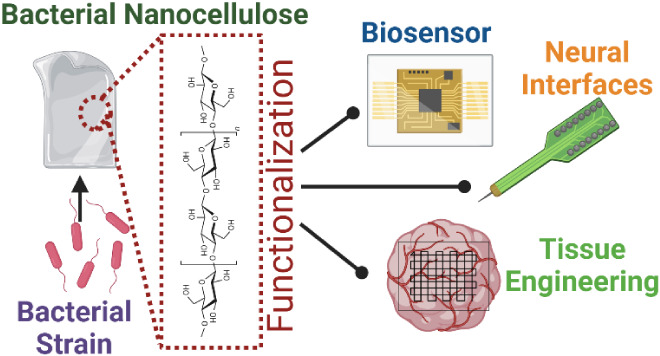

## 1 Introduction

Cellulose, the most abundant biopolymer on Earth with an estimated annual production of over 75 billion metric tons, is synthesized not only by plants but also by certain bacteria.^1^ Among its various forms, bacterial nanocellulose (BNC) or bacterial cellulose (BC), which both are acceptable to use, has gained considerable interest for high-performance applications in bioelectronics due to its unique structural, mechanical, and biochemical properties. First observed in the 1886 by Brown where he called it “vinegar plant”,^2^ BNC emerged in the 1980s and early 2000s as a promising material platform for medical and electronic devices due to its purity, crystallinity, and biocompatibility.^3^ Unlike plant cellulose, which requires extensive chemical treatment to remove lignin and hemicellulose, BNC is biosynthesized extracellularly as a nanofibrous hydrogel, free of these impurities and characterized by a highly porous three-dimensional network. This unique morphology, characterized by nanofibers 20–100 nm wide and a crystallinity of up to 96%, underpins BNC’s exceptional tensile strength, flexibility, and water-holding capacity.^4–7^ These characteristics, combined with excellent biocompatibility and modifiability, have made BNC an attractive material for interfacing with biological systems such as cells, tissues, and biomolecules.^8^ Early studies identified its potential as a scaffold material for biomedical engineering, and recent advances have extended its utility into flexible sensors,^9–12^ wearable devices,^13,14^ implantable electronics,^15^ tissue scaffolds,^16–18^ drug delivery systems,^19–23^ and biosensors.^24–27^

The core advantage of BNC lies in its ability to function chemically or biologically to tailor its surface properties, electrical conductivity, and mechanical behavior. Such customizations allow the development of “smart surfaces” that can modulate protein adsorption, cell adhesion, and molecular recognition—key features for biosensing and antifouling interfaces.^28–35^ Functionalization strategies include the introduction of reactive chemical groups (e.g., aldehydes, carboxyls, and quaternary ammonium), embedding of nanoparticles (e.g., gold, silver, metal oxides), enzymatic grafting, peptide immobilization or boron nitride.^36–38^ These modifications enable BNC to deliver enhanced wettability, selective drug release, and improved electrochemical performance, all while preserving its inherent biocompatibility and bioinertness. Advances in stimuli-responsive materials enhance BNC’s ability to support dynamic biological interactions, as demonstrated by smart hydrogels which integrate with BNC to enable dual pH-sensing and NIR-triggered antibacterial activity or for strain sensors and supercapacitors.^39^ These enable precise control over material–cell interactions in space and time, which is critical for tissue engineering and responsive biosensors.^40–42^ Tailoring properties such as surface chemistry,^43,44^ topography,^45,46^ mechanical strength,^47,48^ porosity,^49,50^ and degradation rate^51,52^ is increasingly essential for the design of next-generation devices. Importantly, functionalized 3D BNC composites have demonstrated high performance in real-world bioelectronics for thermal management and energy storage.^53^ For example, BNC-based flexible sensors have been fabricated for physiological monitoring like electroencephalogram (EEG) measurement,^54^ while multifunctional BNC materials—reinforced with conducting polymers, carbon nanomaterials, or doped with ionic liquid have enabled neural interfaces and wearable energy storage platforms.^33,55^ Studies have also shown that enzyme- and peptide-functionalized BNC can facilitate sensitive detection of analytes like glucose, dopamine, and heavy metals.^19,34^

Despite these advances, a gap remains in consolidating functionalization strategies that improve BNC’s conductivity, optical response, biocompatibility, and mechanical properties for bioelectronic applications. This review explores the application of functionalized bacterial nanocellulose in smart bioelectronics, covering its physicochemical properties, functionalization methods, and integration into devices for biosensing, neural interfacing, and tissue engineering.

Drawing from recent developments, we examine how BNC’s unique structure–property relationships like how to improve pore size uniformity and porosity, thereby reducing impedance and facilitating ion migration^35^—coupled with its renewable nature and customizability—make it a sustainable and high-performing platform for future bioelectronic innovations.

In addition, we highlight key challenges and opportunities for advancing the role of BNC within polymer science and its broader application in diagnostics, therapeutics, and regenerative technologies.

## 2 Properties of Bacterial Nanocellulose

Cellulose is primarily obtained from trees and cotton plants.^56^ However, plant-derived cellulose (PC) contains impurities such as lignin, pectin, and hemicellulose, which require chemical treatments for purification—altering its physical and mechanical properties. Alternatively, bacterial nanocellulose (BNC), synthesized by certain bacteria, fungi, tunicates, and algae, is free of these contaminants and boasts superior crystallinity and molecular weight.^57^ These qualities enable BNC to serve a broad range of applications in biotechnology, pharmaceuticals, and cosmetics.^58^ It forms hydrogels with exceptional water absorption capacity and mechanical strength, making it suitable for wound healing and tissue regeneration due to its biocompatibility.^59^ While plant cellulose and BNC share the same molecular structure, they differ significantly in physical attributes. Wang et al.^7^ provided a comprehensive comparison of these differences, as summarized in Table 1.

**Table 1:** Comparison of Properties of Bacterial Nanocellulose (BNC) and Plant-Based Cellulose (PC)^a^

**Properties**	**BNC**	**PC**
Tensile Strength (MPa)	20–300	25–200
Young’s Modulus (MPa)	Sheet:20000, Single Fiber:130000	2.5–0.170
Water Holding Capacity (%)	>95	25–35
Size of fibers (nm)	20–100	Micrometer Scale
Crystallinity (%)	74–96	40–85
Relative hydrophilicity (%)	40–50	20–30
Purity (%)	>99	<80
Degree of polymerization	14000–16000	300–10000
Porosity (%)	>85	<75
Total Surface Area(m^2^/g)	>150	<10

a Reproduced with permission from ref.^7^. Copyright 2019 Elsevier.

Unlike plant cellulose, which is entangled with polysaccharide impurities and waxes, bacterial nanocellulose contains fewer impurities—primarily bacterial cells, organic acids, salts, residual sugars, and metabolites—which are easier to remove using simple washing, filtration, or mild chemical extraction.^60^ This structural purity makes BNC particularly suited for biomedical engineering, food packaging, and textiles, whereas PC is predominantly used in paper products, textiles, and construction materials. Bacterial cellulose is a translucent, jelly like pellicle formed by a network of water molecules bound via hydrogen bonds. It can be synthesized by several microorganisms, including fungi, algae, and bacteria, through static or agitated fermentation processes.^57^ Table 2 enumerates the milestones in the development and application of bacterial nanocellulose.

**Table 2:** Milestones in the Development and Application of Bacterial Nanocellulose

**Year**	**Category**	**Milestone Description**	**References**
1886	Discovery	First observation of cellulose production by *Acetobacter xylinum* (now *Komagataeibacter*)	^2^
1954	Culturing	Developed Hestrin-Schramm (HS) medium for culturing BNC-producing bacteria	^74^
1976	Structure	First detailed EM studies of BNC’s crystalline and nanofibrillar structure	^94^
1983	Plants	Control Plant Infection	^95^
1990	Genetic Studies	Identified key genes involved in BNC biosynthesis	^96^
1993	Commercial Production	Applied Bacterial Nanocellulose in high-fidelity acoustic membranes (e.g., speaker diaphragms)	^97^
1997	Bioreactor Design	Introduced rotating disc bioreactors to enhance BNC productivity	^98^
2004	Tissue Engineering	Bacterial cellulose scaffolds for cartilage repair	^99^
2006	Nanocomposites	Created BNC-based composites with polymers and nanoparticles for enhanced properties	^100^
2008	Electronics	Explored BNC in flexible electronics and display substrates	^101^
2010	Food Packaging	Bacterial Cellulose Embedded with ε-Polylysine for Sausage Casing	^102^
2013/2018	Antimicrobial Applications	Functionalized BNC with chitosan or peptide	^103,104^
2017/2018	Biomedical Engineering	Developed 3D Printed BNC scaffolds for biomedical implants	^105,106^
2020	Aerogels and Thermal Use	Fabricated lightweight, thermally insulating BNC aerogels	^107^
2020	Coatings	BNC coatings for 3D objects for biomedical applications	^108^
2023/2024/2025	Electronics	3D printing, supercapacitors, cellulose-based separator	^35,39^ ,^53^

Li et al. identified 30 cellulose-producing bacteria, primarily from *Proteobacteria*—especially the *Acetobacteraceae* family.^61^ Only two species were Gram-positive, while the rest, including genera like *Acetobacter, Azotobacter, Rhizobium, Pseudomonas*, and *Salmonella*, were Gram-negative. These bacteria secrete cellulose to adhere to plant cells and protect themselves from UV radiation and antibiotics. Bacterial cellulose production follows an aerobic pathway consisting of four stages:^62,63^ (1) phosphorylation of glucose to glucose-6-phosphate by glucokinase; (2) isomerization to glucose-1-phosphate by phosphoglucomutase; (3) conversion to UDP-glucose via UDP-glucose pyrophosphorylase; and (4) polymerization into cellulose-by-cellulose synthase. Glucose chains are extruded through pores in bacterial cells and self-assemble into microfibrils, forming a 3D reticulated network. These glucan chains consist of β-d-glucopyranose units linked by β-1,4-glycosidic bonds. Cross-linking through hydrogen bonds organizes them into subfibrils, microfibrils, and flat ribbons.^64^

To further understand the BNC biosynthesis, for instance, in Komagataeibacter hansenii, *cdg1* and *cdg2* encode DGC genes (*dgc1* and *dgc2*), PDE-A (*pdea1* and *pdea2*), and four additional c-di-GMP PDEs (*pdea3*–*pdea6*). These proteins show strong structural conservation and isoenzyme packaging similarity.^24^ Sequence analyses of DGC and PDE-A domains reveal shared GGDEF and EAL motifs. Figure 1a illustrates the signal transduction pathway regulating c-di-GMP levels, which influences cellulose biosynthesis.^65^

**Figure 1:**
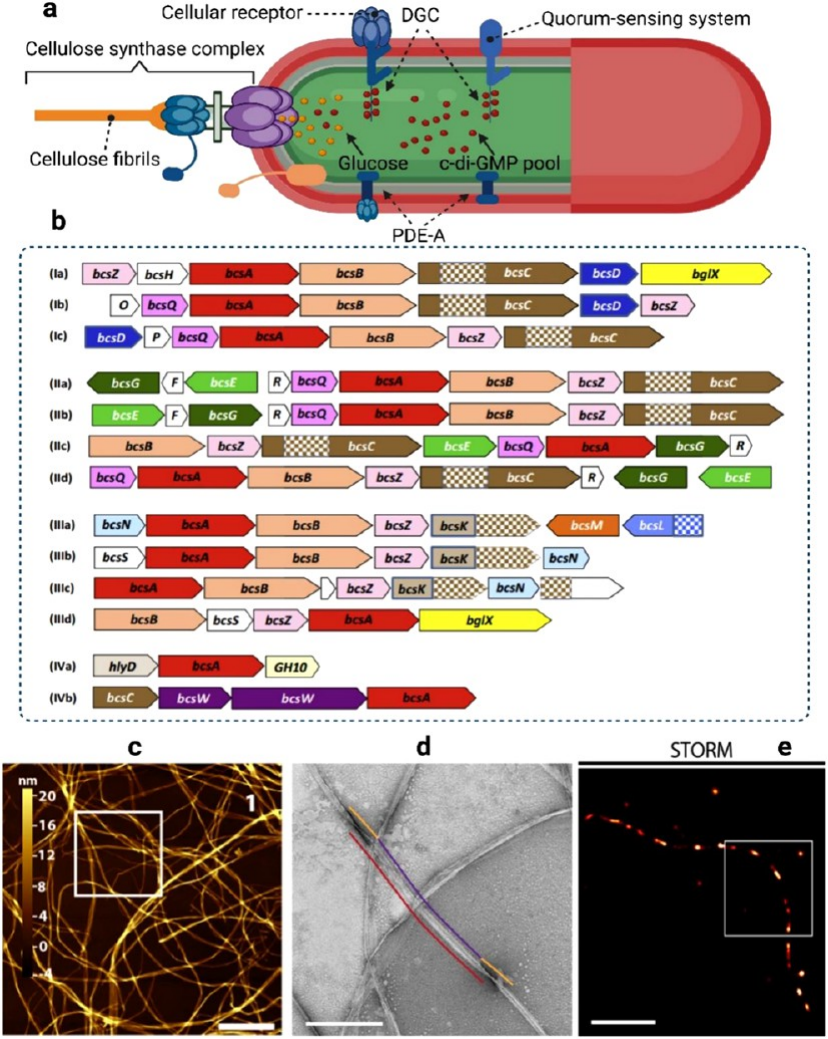
A schematic representation of signal transduction and cellulose production in a bacterial cell (a). Adapted with permission from ref.^65^. Copyright 2022 Elsevier. Diversity of the bacterial nanocellulose synthase (bcs) operons (b). Reproduced with permission from ref.^66^. Copyright 2015 Elsevier. BNC morphological images: AFM (c); TEM (d); STORM (e). Reproduced with permission from ref. 67. Copyright 2023 American Chemical Society.

External factors such as light, temperature, and electrical currents modulate c-di-GMP levels via quorum sensing or direct environmental input. Medium components like oxygen, CO, NO, and nutrients are sensed by DGC and PDE-A receptors, resulting in upregulation or inhibition of cellulose synthesis based on c-di-GMP activation levels. The bcsABCD operon responsible for cellulose biosynthesis was first identified in *Komagataeibacter xylinus*. *BcsA* and *BcsB* are essential for in vitro activity, whereas *BcsC* and *BcsD* are required in vivo for proper cellulose export and assembly. Mutants lacking *bcsC* cannot form cellulose fibrils, and *bcsD* mutants exhibit a 40% reduction in cellulose yield. The *K. xylinus bcs* locus also contains upstream genes *eng* (*bcsZ*) and *ccpA*, and downstream gene *bglX*. Variations in the bcs operons across different bacterial species are also presented in Figure 1b, showing gene organization, open reading frames, and domain elements such as TPR repeats.^66^

The morphology and nanoscale organization of BNC have been examined using techniques like AFM, TEM, and STORM.^67^ AFM images reveal BNC as a fibrous mesh with ribbons of differing widths and heights, Figure 1c. TEM further detail the nonpersistent, right-handed twist of the cellulose ribbons, which generally lie flat against the substrate, Figure 1d. These ribbons feature alternating twisted (orange lines), flat (purple lines), and spaced (red lines) regions. STORM super-resolution imaging, Figure 1e, confirms that the dislocations—bright spots—correspond with twisting areas observed as dark bands under TEM. Bacterial cellulose’s unique mechanical and structural properties arise from its dense hydrogen bonding and 3D fibrillar architecture.^68^

These contribute to its high tensile strength, flexibility, and biocompatibility.^69,70^ Its hydrophilic nature, driven by abundant hydroxyl groups, enhances its water retention capacity, while its crystalline architecture ensures thermal stability.^71^ These features render BNC a promising material across fields like regenerative medicine, electronics, filtration, and sustainable packaging. Multiple variables affect BNC production, including bacterial strain, medium composition, and environmental conditions.^72,73^ *Komagataeibacter xylinus* remains the most efficient producer. Common culture media—Hestrin–Schramm,^74^ Yamanaka’s, or Zhou’s—are supplemented with carbon and nitrogen sources.

Studies using substrates such as maple syrup,^75^ sucrose,^76^ coffee husk,^77^ mango peel,^78^ and paper sludge^79^ illustrate their influence on yield. Optimal pH (4.32–7.2),^80,81^ temperature (25–33 °C),^81–84^ and oxygen levels (up to 30% DO)^85^ are crucial. Static culture methods yield consistent pellicles after 5–6 days of incubation, with optimal yields typically observed within 7 to 14 days.^58,86,87^ Different cultivation strategies—static, agitated, or reactor-based—impact BNC morphology and scalability.^87^ The typical production yield of BNC is reported to range from 0.17 to 2.06 g/L per day,^88^ with production costs between 0.56 and 60.84 USD per kg^89^ or 0.14 to 11 AUD per gram,^90^ whereas plant-based cellulose yields approximately 22.8% to 92%^91^ and costs about 1.17 to 14.94 EUR per kg.^92,93^

Agitated systems produce irregular forms while reactor-based approaches like membrane or aerosol bioreactors offer controlled environments for membrane synthesis. Feeding intervals also affect continuous production; nutrient addition to upper BNC layers can maintain productivity by ensuring concurrent oxygen and nutrient access.

## 3 Functionalization Techniques

Herein, we examined the various ways in which BNC can be modified to enhance its functionality in smart bioelectronics specifically to improve the following properties such as (1) conductivity via electrostatic assembly, layer-by-layer assembly, nanoparticle functionalization and carbonization; (2) optical properties by quantum dots incorporation or addition of photonic crystals; (3) biocompatibility via enzymatic functionalization, peptide immobilization, and ionic liquid doping; and (4) mechanical strength through covalent cross-linking, glyoxalization cross-linking, and carbon-based materials integration. These modifications enable the development of smart bioelectronic applications.

### 3.1 Conductivity

An essential obstacle in incorporating BNC into bioelectronics is its intrinsic deficiency in conductivity.^109^ Functionalizing BNC to become electrically active is an essential stage in the development of bioelectronic devices, as these devices heavily depend on conductive materials for their operation, ranging from sensors^110^ to energy storage systems like supercapacitors.^111–114^ Electrostatic assembly is a commonly used method to functionalize bacterial nanocellulose (BNC) with conductive polymers like polypyrrole (PPy) or PEDOT, improving its electrical conductivity for device applications.^115^ In Muller et al. study, BNC membranes were immersed in pyrrole solutions, and using APS or FeCl3 as oxidants, flexible BC/PPy composites with electrical conductivities up to 1.2 S cm^–1^ were obtained.^116^ SEM revealed PPy nanoparticles uniformly distributed on BNC nanofibers. The study showed that oxidant type and monomer concentration control the properties of BC/PPy composites. Shao’s group developed PPy/BC nanofiber composites using a cost-effective polymerization method, achieving an adsorption capacity of 555.6 mg g^–1^ for Cr­(VI) removal.^117^ The process was pH-dependent, and the PPy/BC composites demonstrated superior operability and recyclability for wastewater treatment. Wang et al. fabricated PPy-carbon cloth (CC) with BNC electrodes for capacitive antimicrobial dressings, which showed excellent bactericidal efficiency through physical stimulation and promoted wound healing in a mouse model. This study offers a potential antibiotic-free solution for infected wound treatment.^118^

Graphene Oxide integration via Layer-by-Layer (LbL) assembly is a functionalization method alternating adsorption of oppositely charged conductive species, a multilayer conductive structure can be built on BNC.^119^ This method offers precise control over the material’s conductivity and noted for its exceptional electrical characteristics into the BNC network. This technique not only increases the ability of the material to conduct electricity but also boosts its mechanical flexibility, making it well-suited for use in wearable electronic devices.^120,121^ Torres et al. investigated BNC films reinforced with reduced graphene oxide (RGO) platelets, aiming to assess their potential as solid polymeric electrolytes. They incorporated GO into the BNC network and reduced it in situ using hydrazine vapor. Their results showed that the incorporation of 5% NH_4_I improved ionic conductivity, reaching 1.32 × 10^–4^ S/cm. Their method maintained the 3D morphology of BNC while increasing conductivity, making the composite suitable for electrochemical applications.^122^ Ccorahua et al. followed a similar approach but focused on reinforcing BNC with RGO using hydrazine treatment. This treatment removed oxygen-containing groups from graphene, increasing the conductivity of the BC-based films from an insulating state to an average of 12 S/m. Raman spectroscopy confirmed the presence of graphene, and XRD tests showed no structural changes in BNC, indicating a successful enhancement of conductivity without compromising the material’s integrity.^114^ Feng et al. employed vacuum-assisted self-assembly to create a flexible nanocomposite film of BNC and GO. The GO nanosheets were uniformly dispersed in the BNC matrix, as confirmed by microscopic and X-ray diffraction analyses. The addition of 5 wt % GO improved Young’s modulus and tensile strength by 10% and 20%, respectively. Furthermore, the electrical conductivity increased by 6 orders of magnitude compared to unmodified BNC after in situ reduction of GO.^123^

Another sophisticated technique entails the utilization of silver nanoparticle functionalization, wherein minuscule silver particles are deposited onto the BNC surface. Parnsubsakul et al. developed a biodegradable, flexible SERS substrate using BNCP composited with AgNPs for rapid pesticide detection on fruit peels. The substrate demonstrated good SERS activity and signal uniformity and demonstrated potential for *in situ* SERS detection. The paste-and-read SERS method is simple, rapid, and low-cost, making it promising for developing novel active substrates based on green material selection in the future.^124^ Another study of Wang et al, bacterial nanocellulose was synthesized and layered with silver nanowires (AgNWs) through a step-by-step in situ biosynthesis, with repeated cycles of spraying AgNW-dispersed medium onto BC membranes, producing a robust BC/AgNW composite. After a 3-day fermentation, the composite was purified and hot-pressed, creating highly flexible and conductive BC/AgNW papers.

The highest AgNW-content paper, BC/AgNW^–4^, achieved an impressive conductivity of 608 S m^–1^ and a high specific shielding effectiveness (SSE) of 6400 dB mm^–1^. This durable, washable material, up to 500 cycles, offers potential for EMI shielding applications in flexible electronics.^125^ The conductive characteristics of silver nanoparticles are widely recognized, and their incorporation into BNC greatly enhances the material’s electrical performance. This technique has been utilized in the development of flexible electronic circuits and biosensors, where the combination of conductivity and flexibility is of utmost importance. For carbonization technique which is used for applications that require stronger conductivity. By subjecting BNC to high temperatures in a process called pyrolysis, the cellulose fibers undergo a transformation and become carbon structures, such as carbon fibers or carbon dots. These carbon structures have exceptional conductivity. These carbonized structures are suitable for application in energy storage devices such as supercapacitors, where both high conductivity and endurance are crucial.

In Wang et al. study, food-grade bacterial nanocellulose cubes were processed by immersing them in sucrose syrup, followed by rinsing in Milli-Q water to remove excess syrup, and autoclaving at 120 °C for 20 min. The cubes were then solvent-exchanged using 1-butanol, dried, and carbonized at 900 °C under an argon flow. The resulting porous carbons displayed significantly higher porosity compared to those treated with water, yielding an optimal structure for use as a cathode in Li–O_2_ batteries. The carbon derived from 1-butanol treatment exhibited superior performance with a capacity of 5.58 mA h cm^–2^, lower overpotentials, and longer cycling life, outperforming even commercial cathode materials like Super P, indicating its potential for various energy storage applications.^126^ While in the review paper by Ma et al, they consolidated BC/BNC derived 3D carbon nanomaterials, such as carbon aerogels, reduced graphene oxide (RGO) foam, and carbon nanotube (CNT) sponges, that were synthesized to leverage their high specific surface area, hierarchical pore structure, electrical conductivity, and chemical stability. The approach involved pyrolyzing biomass precursors, which enables easy fabrication and environmental compatibility, to create conductive 3D carbon aerogels. Activation treatments significantly enhanced the specific surface area, achieving values up to 1037 m^2^/g. Additionally, heteroatom doping strategies, including annealing with heteroatom-rich compounds, resulted in improved electrochemical properties. For example, N-doped carbon nanofibers exhibited a specific capacitance and power density of 390.53 kW/kg in all-solid-state supercapacitors, demonstrating their potential for energy storage applications and as electrocatalysts for various reactions.^127^ Recently, the emergence of MXene- and MoS_2_-based BNC composites has enabled the development of high-performance materials, including a 3D BNC-intercalated MoS_2_@rGO nanocomposite with a specific capacitance of 345 F g^–1^,^128^ and MXene–hydroxylated CoFe_2_O_4_ nanoparticle (h-CFO NP)/BNC hybrids designed to modulate the electromagnetic performance of wave-absorbing materials (EWAMs).^129^

As shown in Figure 2a–h, various BNC-based nanocomposites have been fabricated through integration with conductive nanomaterials such as RGO, PPy, and AgNWs. For instance, the fabrication of nitrogen-doped pyrolyzed bacterial nanocellulose embedded with RGO (PBC-RGO-N) is illustrated in Figure 2a,^111^ the fabrication process of PPy/RGO/BNC paper electrodes is presented in Figure 2b,^115^ FESEM imaging of CBC-N3 nanofibers is provided in Figure 2c^100^ while FEG-SEM micrographs of BNC/FeCl_3_ samples are shown in Figure 2d.^116^ Furthermore, the introduction of RGO into the BC network forms a three-dimensional interconnected conductive path, as exemplified in Figure 2f, which shows a cross-sectional SEM image of BNC/5 wt % GO.^123^ Similarly, PPy coatings enhance charge transport by providing continuous electron pathways along the BNC nanofibers, as observed in the SEM image of PPy/BNC, Figure 2e.^117^ AgNWs embedded within the BNC matrix further boost electrical conductivity and stability. This is demonstrated in Figure 2g, showing a cross-sectional SEM image of BNC/AgNW-4 paper, and in Figure 2h, which highlights that the electrical resistance of the BNC/AgNW papers did not significantly change even after exposure to solvents such as water, ethanol, vegetable oil, and NaCl aqueous solution.^125^ Collectively, these hybrid structures mitigate the intrinsic limitations of individual components—such as GO restacking, the insulating nature of BNC, and polymer aggregation—resulting in nanocomposites with improved conductivity, robust layered morphology, and suitability for applications such as supercapacitors and EMI shielding.

**Figure 2:**
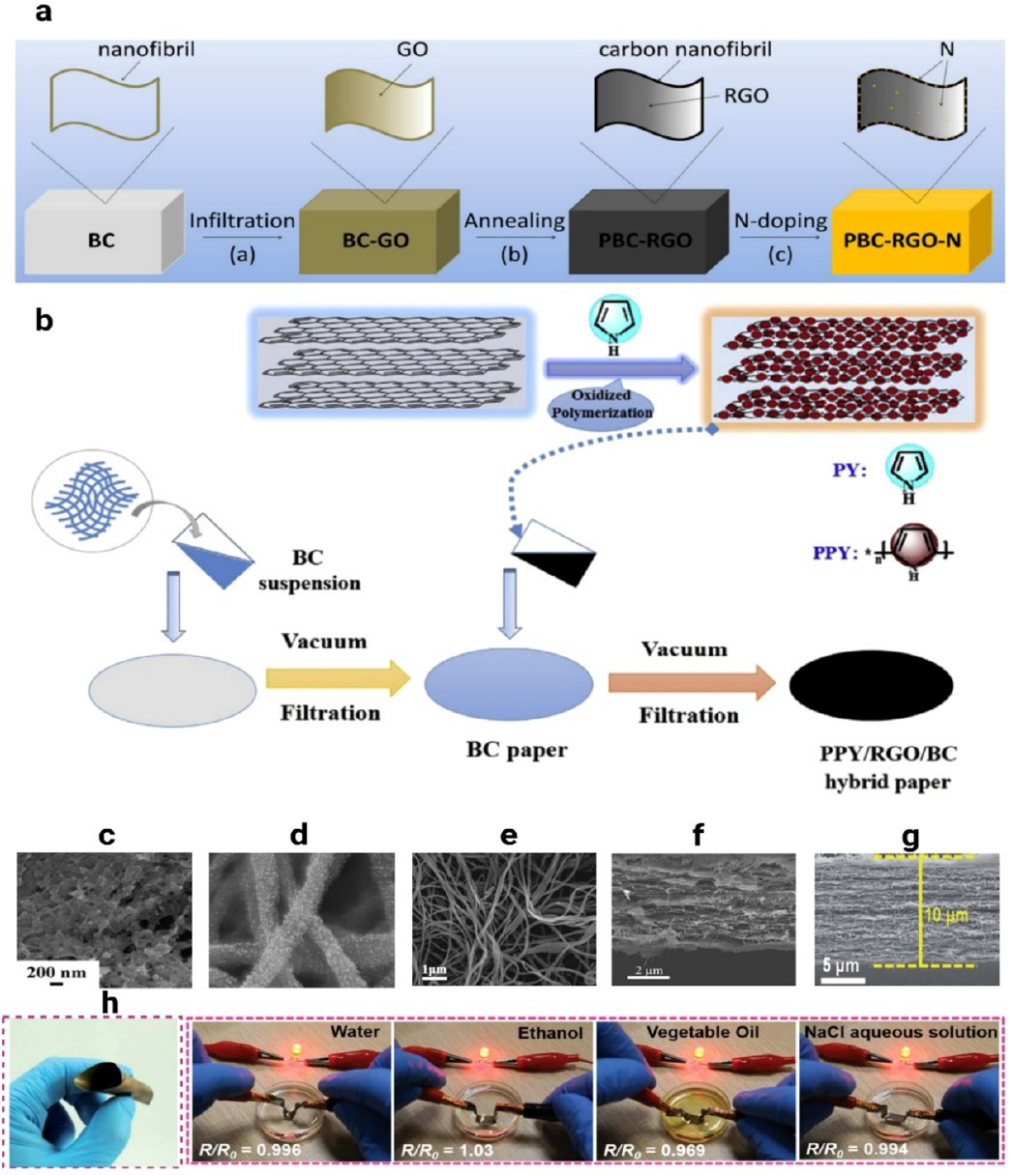
Fabrication of RGO-embedded nitrogen-doped pyrolyzed bacterial nanocellulose (PBC-RGO-N (a). Reproduced with permission from ref.^111^. Copyright 2017 Wiley. Fabrication process of PPY/RGO/BNC paper electrode (b). Reproduced with permission from ref.^115^. Copyright 2016 Elsevier. FESEM image of CBC-N3 nanofibers (c). Reproduced with permission from ref.^100^. Copyright 2016 Wiley. FEG-SEM micrographs of BNC/FeCl_3_ samples (d). Reproduced with permission from ref.^116^ Copyright 2013 Elsevier. SEM image of PPy/BNC (e). Reproduced with permission from ref.^117^. Copyright 2021 Springer Nature. Cross-section SEM image of BNC/5 wt % GO (f). Reproduced with permission from ref.^123^. Copyright 2012 Elsevier. Cross-sectional SEM image of the BNC/AgNW-4 paper (g); the electrical resistance of the BNC/AgNW papers which did not significantly change after exposure to water, ethanol, vegetable oil and 1 M NaCl aqueous solution (h). Reproduced with permission from ref.^125^. Copyright 2022 Royal Society of Chemistry.

### 3.2 Optical Property

Besides conductivity, numerous bioelectronic devices necessitate materials that are light-responsive or capable of functioning as optical sensors.^18^ Incorporation of Boron nitride into bacterial nanocellulose for optical/sensing applications is still unexplored research that can be modified with surface plasmon resonance (SPR) by incorporating metal nanoparticles, such as gold or silver, onto its surface for which these nanoparticles demonstrate plasmonic characteristics, which can be utilized for optical sensing applications.^130^ However, sensors based on surface plasmon resonance

(SPR) may identify alterations in the refractive index of a substance, which makes them extremely responsive to biological interactions. One alternative approach to incorporating optical characteristics into bacterial nanocellulose is by conjugating it with quantum dots. Quantum dots are tiny particles made of semiconductors that have optical properties that vary depending on their size, including the ability to emit light. By incorporating quantum dots into BNC, the material can be converted into an exceptionally responsive optical sensor that can detect precise biological molecules or environmental alterations. For instance, Zhang et al. group, developed a composite BNC membrane by incorporating CdTe quantum dots (QDs) with the quantum yield of 45.6% and red fluorescent RB probes to create a multicolor fluorescent method for pH detection in paper artifacts.

The process involved preparing a Cadmium-Thioglycolic acid (Cd-TGA) solution, followed by the immersion of the neutral BC membrane to achieve adsorption equilibrium, and subsequent treatment with Na_2_TeO_3_ and NaBH_4_ to synthesize CdTe QDs. RB probes were synthesized from RB hydrazide and incorporated into the membrane after adsorption. The resulting composite membrane exhibited a strong photoluminescence (PL) response, emitting green fluorescence under neutral conditions (pH 7.5) and transitioning to red fluorescence under acidic conditions (pH 3.0), allowing for visual detection of pH values between 3.0 and 7.5. This method provides accurate quantification and clear visualization of pH distribution, making it a promising tool for advanced analysis in preserving paper-based cultural relics.^131^ Meanwhile, Celi et al. group, the CdTe-TGA quantum dots (QDs) were synthesized through a process involving the reaction of CdCl_2_ and TGA, followed by the addition of NaOH to achieve a basic pH, and the introduction of a chalcogenide precursor, NaHTe, at 100 °C for 4 h, resulting in QDs with a quantum yield of 43%. These QDs were then purified and assembled with bacterial nanocellulose (BNC) to create a nanocomposite chemosensor. Morphological and structural analyses confirmed the integration of nanofibrils and QDs within the BNC matrix. While the chemosensor demonstrated challenges in compatibility with the DTZ reagent, it effectively detected Hg^2+^ ions, exhibiting noticeable fluorescence changes observable by the naked eye. The combination of low-cost, biocompatible, and biodegradable BNC with high-sensitivity QDs presents a promising platform for the development of optical sensing technologies.^132^ Moreover, Morales-Narváez et al. study, explored the utilization of Bacterial cellulose (BC) as both a reducing agent and template to synthesize silver nanoparticles (AgNPs) and gold nanoparticles (AuNPs) in situ, achieving distinct color changes that confirmed nanoparticle formation. The AgNP-BC and AuNP-BC composites were fabricated through reactions with AgNO_3_ and HAuCl_4_,respectively, followed by thorough washing and storage. The successful synthesis was validated using UV–visible spectroscopy, energy-dispersive X-ray spectroscopy (EDX), and scanning electron microscopy (SEM). Furthermore, the BNC was modified through TEMPO-mediated oxidation to introduce carboxyl groups, allowing for the attachment of photoluminescent nanoparticles (PL-NPs) via EDC/NHS chemistry. The resulting nanopaper-based composites exhibited versatility for optical sensing applications, demonstrating high selectivity and specificity through biomolecular functionalization, and potential use in diagnostics, environmental monitoring, and wearable sensors due to their lightweight, biocompatible, and biodegradable nature.^133^

Another potential avenue for new research and discoveries is the inclusion of photonic crystal in bacterial nanocellulose as a method that utilizes the distinctive characteristics of photonic crystals to produce materials capable of controlling light. Researchers can create materials with the ability to selectively reflect or transmit specific wavelengths of light by including photonic crystals into the Bacterial nanocellulose network. These materials are well-suited for use in optical filters or as components in photonic circuits.^55^

One interesting recent research from Caligiuri et al, that a film-forming Luria–Bertani (LB) solution and a xylan-blended version (x-LBA) were prepared for micro- and nanostructuring, with solutions autoclaved and poured onto master molds where the resulting biodegradable photonic structures effectively balanced moisture retention and cell viability, enabling the creation of eco-friendly photonic architectures and quantum optical framework.^134^ Morphological and diffractive analyses validated the suitability of both LB and x-LBA for soft lithography, with additional features such as controllable surface wettability and plasmonic properties achieved by silver sputtering, paving the way for innovative bioplasmonic applications.^134^ This study will be a good new pathway to optimize the media use to grow bacterial nanocellulose while synthesizing photonic crystals.

As shown in Figure 3a–b, diverse strategies have been employed to functionalize bacterial cellulose (BC) or bacterial nanocellulose (BNC) with optically active nanomaterials to achieve tunable photoluminescent properties. The abundant hydroxyl groups and porous network of BC facilitate the in situ growth and uniform embedding of CdTe quantum dots (QDs) and Rhodamine B (RB) fluorescent probes, where hydrogen bonding serves as the primary anchoring mechanism, enabling efficient fluorescence under UV excitation and pH-responsive emission modulation. The hot-injection method for synthesizing monodispersed CdTe QDs ensures high quantum yield and stability, while RB-derived probes further provide tunable emission through structural modification. Representative plasmonic and photoluminescent nanopapers—including AgNP-BC, AuNP-BC, QD-BC, and UCNP-BC—are presented in Figure 3a highlighting the versatility of noble metal nanoparticle incorporation for optical enhancement.^133^ In addition, the fabrication of nanopaper-based chemosensors with varying QD concentrations is shown in Figure 3b, demonstrating the ability to fine-tune photoluminescent responses for sensing applications.^132^ Furthermore, BC/BNC membranes have been explored as scaffolds for surface carboxylation or integration with conductive nanomaterials such as GO, thereby broadening responsive optical behavior and enhancing overall performance. Collectively, these functionalized BNC-based composites hold significant promise for next-generation sensing, bioimaging, and flexible optoelectronic devices.

**Figure 3:**
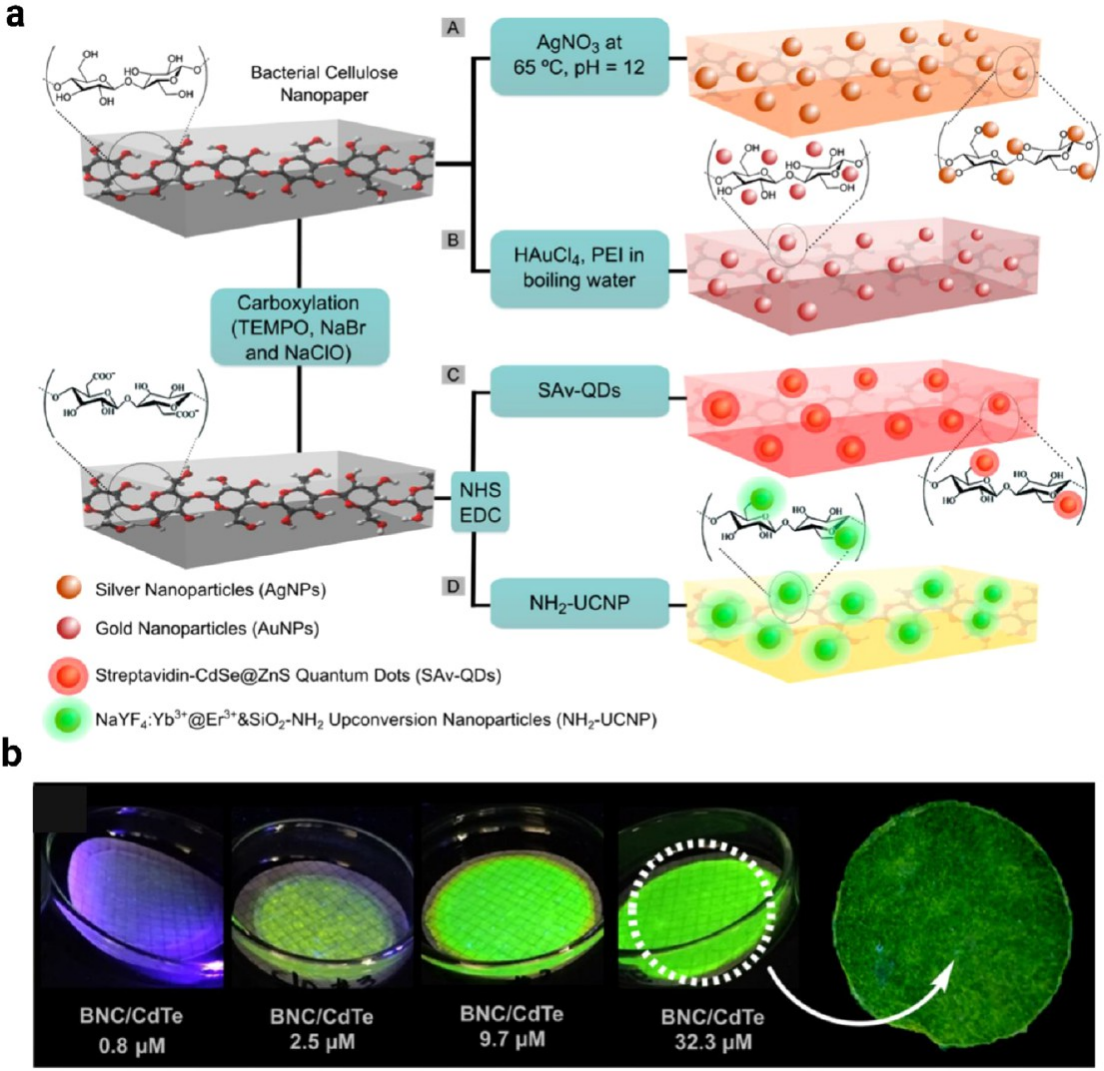
(A) AgNP-BC, (B) AuNP-BC, (C) QD-BC, and (D) UCNP-BC, representing plasmonic and photoluminescent nanopapers (a). Reproduced with permission from ref.^133^. Copyright 2015 American Chemical Society; Preparation of nanopaper specimens chemosensor with varying QD concentrations (b). Reproduced with permission from ref.^132^. Copyright 2023 Royal Society of Chemistry.

### 3.3 Biocompatibility

For BNC to be appropriate for biomedical applications like biosensors or tissue scaffolds, it must have biocompatibility and the capacity to interact with biological systems.^135^ The process of enzymatic functionalization of protein-based fibers involves the incorporation of enzymes such as laccase or glucose oxidase.^136^ These enzymes act as biocatalysts, allowing BNC-based biosensors to detect certain biochemical reactions, such as the presence of glucose in a blood sample.^137^ For instance, Wang et al. studied the details on the development of a bienzymatic biosensor utilizing a gold nanoparticles-bacterial cellulose (Au-BC) nanocomposite immobilizing glucose oxidase (GOx) and horseradish peroxidase (HRP) on a glassy carbon electrode (GCE). The electrode was prepared by polishing the GCE, applying the Au-BC suspension, and incubating it with enzyme solutions, with poly­(diallyldimethylammonium) (PDDA) preventing enzyme leakage.

The resulting PDDA/GOx-HRP/Au-BC/GCE demonstrated a sensitive amperometric response to glucose, maintaining high enzyme activity, with a detection limit of 2.3 mM and a linear range from 10 mM to 400 mM. This biosensor exhibited fast response times, stability, and good reproducibility, making it suitable for glucose detection in human blood samples.^138^ While in Li et al. study, palladium nanoparticle-bacterial cellulose (PdBC) hybrid nanofibers were synthesized using an in situ chemical reduction method, with palladium nanoparticles evenly dispersed on bacterial cellulose surfaces to enhance electrocatalytic effects. The PdBC nanofibers were combined with laccase (Lac) and Nafion to create a suspension, which was then used to modify a glassy carbon electrode, resulting in the GCE/Lac-PdBC-Nafion biosensor. This electrochemical biosensor demonstrated high sensitivity (38.4 μA·mM^–1^), a low detection limit (1.26 μM), and a wide linear range (5–167 μM) for dopamine detection, along with excellent repeatability, reproducibility, selectivity, and stability. It was successfully applied for dopamine

analysis in human urine, highlighting its potential for clinical applications.^139^ Almost similar to another Li et al. group, wherein a biosensor was constructed by attaching BC-AuNPs to a glassy carbon electrode, followed by the addition of laccase (Lac) and Nafion.^140^ This electrochemical biosensor demonstrated excellent electrocatalysis for hydroquinone detection, achieving a low detection limit of 5.71 nM and a wide linear range of 30–100 nM. The biosensor exhibited good repeatability, reproducibility, selectivity, and stability, successfully detecting hydroquinone in lake water samples, highlighting its potential for environmental monitoring.

For peptide immobilization, which refers to the process of attaching bioactive peptides to the surface of BNC, these peptides can be engineered to selectively bind to proteins, cells, or enzymes, making them well-suited for use in biosensing or tissue engineering.^141^ Peptide-functionalized bacterial nanocellulose (BNC) has great significant potential in the advancement of biosensors with exceptional selectivity and sensitivity for the detection of disease indicators, pathogens, and other biomolecules. For instance, in Kim et al. study, bacterial cellulose nanofibrils (BCNFs) were modified by conjugating the Bac7 peptide using EDC/NHS chemistry, resulting in a biocompatible BCNFBac7 complex.

This system exhibited enhanced skin adhesion through hydrophobic and electrostatic interactions with cell membranes, which significantly improved water retention on the skin and reduced evaporation during topical applications. The successful conjugation and structural characteristics were confirmed through various analytical techniques, including NMR, FT-IR, and UV–vis spectroscopy. This peptide-mediated BCNFs skin adhesion system shows promise for applications in dermal therapies, such as treating atopic dermatitis.^142^ Another effective technique was developed by van Zyl et al. group, herein, the chimeric peptides combining the antimicrobial peptide KR-12 with carbohydrate binding peptides (CBPs) were synthesized and designed to noncovalently modify bacterial cellulose surfaces, utilizing flexible linkers to enhance solubility and proteolytic resistance. The secondary structure of the peptides was predicted in silico and confirmed through CD spectroscopy, demonstrating retention of the α-helix structure essential for bioactivity. Both chimeric peptides exhibited antibacterial activity against *E. coli* and *Pseudomonas aeruginosa*, with the Short-CBP-KR12 showing superior lipopolysaccharide binding capabilities. Importantly, these peptides were effectively immobilized on BC over a 7-day period without negatively impacting the metabolic activity of normal human dermal fibroblasts or keratinocytes.^143^

Furthermore, the biocompatibility of BNC is improved by including ionic liquid doping along with enzyme and peptide functionalization. Ionic liquids, which are liquid salts at room temperature, can be infused into BNC to enhance its ionic conductivity and biocompatibility. This characteristic of BNC renders it more appropriate for utilization in implanted devices or bioelectronic interfaces that necessitate enduring stability in biological environments. Munir et al, found that bacterial nanocellulose sheets were rendered biodegradable for potential use in tissue regeneration by treatment with two ionic liquids (ILs): 1-butyl-3-methylimidazolium hydrogen sulfate (BMIM-HSO_4_) and pyridinium hydrogen sulfate (Py-HSO_4_). After optimizing conditions, BC was treated with 75% IL at 100 °C for 20 min, resulting in significant degradation over 28 days—36% for Py-HSO_4_ and 56% for BMIM-HSO_4_. Characterizations (FTIR, SEM, XRD) showed that IL treatment increased hydrophilicity, thinned fibers, and did not alter chemical composition. The IL-treated BC supported cell attachment was nontoxic, and allowed sustained drug release, indicating its potential for degradable implants, drug carriers, and tissue scaffolds likewise although conductivity was not directly measured, swelling and degradation trends suggest a nonlinear impedance trajectory: early (0–7 days) swelling up to 1113% and reduced contact angle (57° → 30°) would lower impedance by roughly an order of magnitude, midstage (14–21 days, 35–49% mass loss) likely stabilizes impedance in the low-kΩ range; and late-stage (28 days, 56% mass loss) would disrupt percolation, driving impedance several-fold above baseline.^144^

As shown in Figure 4a–g, functionalized BNC composites demonstrate broad biomedical applicability through antibacterial delivery, biosensing, and regenerative platforms. Controlled release of antibacterial agents from BNC enables wound healing applications, while drug-loaded BNC systems provide localized cancer immunotherapy with minimal systemic toxicity. In addition, BNC scaffolds have proven effective for tissue regeneration, particularly in chondrogenesis, demonstrating excellent in vivo tolerance without adverse effects. Electrochemical biosensors using BNC nanocomposites as enzyme-immobilized layers on three-electrode systems are illustrated in Figure 4a, while optical detection platforms exploiting fluorescence modulation of BNC membranes are shown in Figure 4b.^137^ Cytotoxicity assays confirm the biocompatibility of ionic liquid-modified BC (BC-IL) and drug-loaded BC-IL membranes, maintaining over 95% fibroblast and HaCaT cell viability across 7 days, Figure 4c.^144^ Similarly, KR-12-functionalized BC slightly enhances HaCaT metabolic activity, Figure 4d.^143^ TEM analysis validates gold nanoparticle incorporation into BNC nanofibers, Figure 4e,^140^ while SEM and fluorescence microscopy images confirm the successful embedding of Bac7 peptides, improving surface adhesion and HaCaT cell interactions, Figure 4f–g.^142^ Collectively, these results highlight the excellent biocompatibility, tunable bioactivity, and strong therapeutic potential of functionalized BNC-based composites.

**Figure 4:**
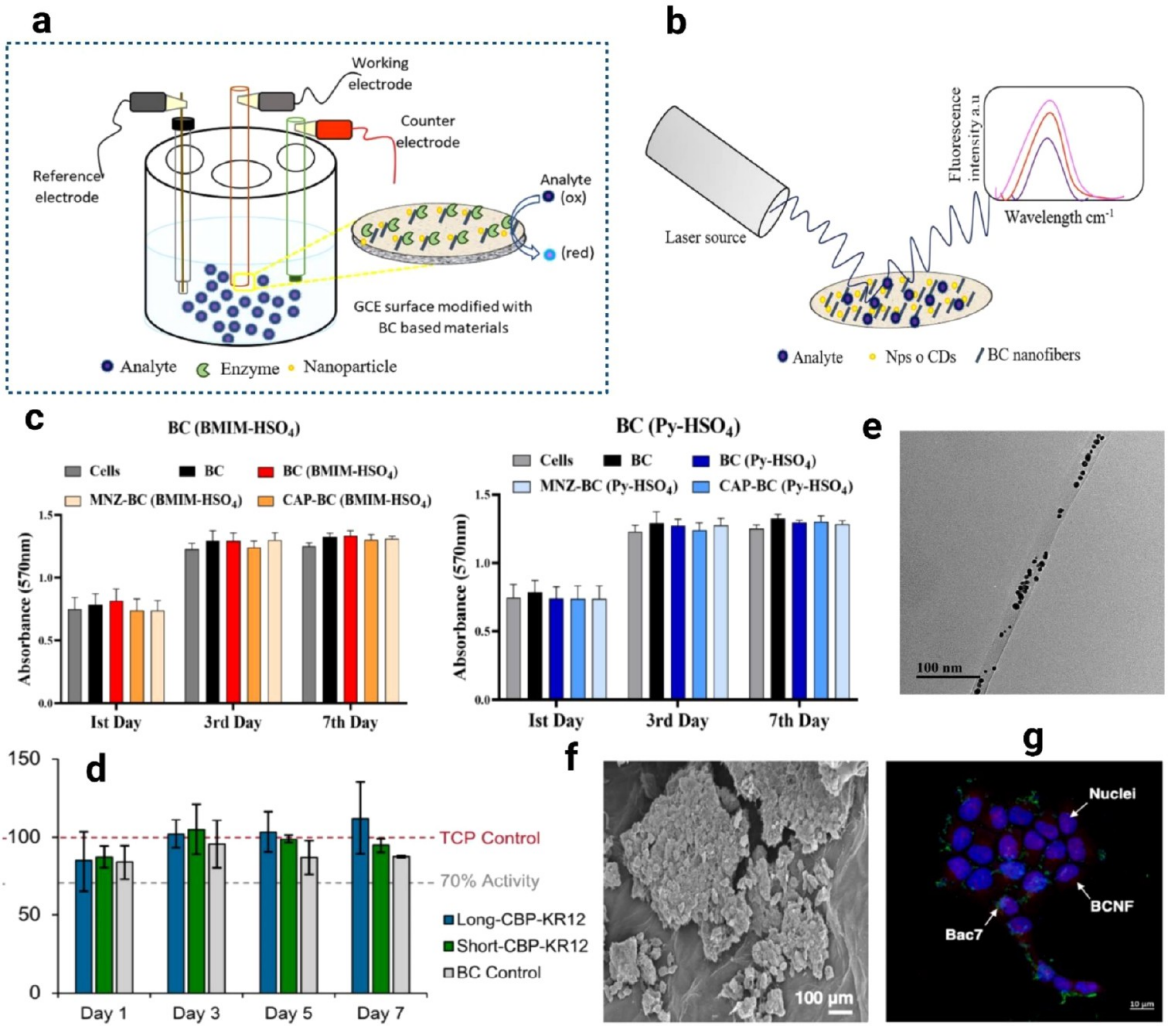
Schematic representation of an electrochemical biosensor with a three-electrode system. The working electrode (GCE) is covered by a BNC nanocomposite on which a suitable enzyme is immobilized (a). Schematic representation of a biosensor in which BNC has been used as a platform for optical detection. In this example, the analyte changes the fluorescence spectrum of a BNC composite membrane (b). Reproduced with permission from ref.^137^. Copyright 2020 Wiley. Cell viability of BNC, BNC-IL and drugs loaded BNC-IL (c). Reproduced with permission from ref.^144^. Copyright 2024 Springer Nature; BNC functionalized with Long-CBP-KR12 and Short-CBP-KR12 normalized to untreated cells (d). Reproduced with permission from ref.^143^. Available under a CC-BY license. Copyright 2024 Van Zyl et al., MDPI. TEM image of BNC-AuNPs hybrid nanofiber (e). Reproduced with permission from ref.^140^. *Copyright 2016 Elsevier*; SEM images of HaCaT cells BCNFBac7 (f). Fluorescence microscopy image of HaCaT cells BCNFBac7 (g). Reproduced with permission from ref.^142^. Copyright 2021 Elsevier.

### 3.4 Mechanical Strength

Although BNC possesses inherent strength, some applications in bioelectronics necessitate additional mechanical reinforcement.^145^ Covalent cross-linking is a method that involves using cross-linking chemicals, like glutaraldehyde,^146–148^ glyoxalization,^149^ and collagen^150,151^ to chemically join bacterial nanocellulose fibers together.

This improves the material’s mechanical strength, increasing its resistance to damage and deterioration. For instance, Brown et al. study found that glutaraldehyde-treatment significantly increased the strain of BNC/fibrin composites, with a 60% difference between untreated and treated composites wherein this treatment introduced an initial low modulus plateau, unlike native BC and untreated composites and it also increased strain at break, improving strength and strain at break.^146^ In addition, Yang et al. work examines the impact of cross-linking degree on tensile strength, elongation at break, and Young’s modulus of a 4% BC/PVA composite membrane with glutaraldehyde as the cross-linker.^148^ The tensile strength and Young’s modulus increased with cross-linking degree, indicating good interfacial adhesion and strong interactions between PVA chains, BNC, and PVA chains. Chemical cross-linking between BNC/PVA composite and glutaraldehyde was formed, resulting in a more compact and rigid three-dimensional structure. The tensile strength and Young’s modulus increased with BNC content, particularly when cross-linking time was 30 min. BNC incorporation significantly enhanced the tensile strength and Young’s modulus of the composite membranes, attributed to the high strength and modulus of BC nanofibrils. The improvements observed in the composite membranes reinforced with BNC could also be attributed to good dispersibility and excellent adhesion between BC and PVA.

For glyoxalization cross-linking, Quero et al. found that glyoxalization reduces stress, strain at failure, and work of fracture in dry BC networks, possibly due to cross-linking between BC layers.^149^ This reduces layer-to-layer mobility and delamination. Researchers investigated the impact of this treatment on dry BC networks using Raman spectroscopy. In the wet state, Young’s modulus and stress at failure decrease, with an increase in strain to failure for wet unmodified BC networks. These changes are due to the disruption of intermolecular hydrogen bonding by water molecules, allowing slippage between BC fibrils. The modulus of wet glyoxalized BC networks is reduced by 77% compared to the dry state, and stress at failure remains unchanged. Cross-links may limit layer-to-layer and nanofiber-to-nanofiber mobility. For collagen-based cross-linker, Sommer et al. found that the results of mechanical tests from highly wrinkled surface of enzymatically cross-linked BC/Col composites can increase their tensile strength.^150^

The highest σ value was found for BC/Col composites cross-linked with Transglutaminase (TGase) at 60–63 MPa. These composites also showed the highest thermal stability, which resulted from their highest CrI and structure strongly cross-linked with covalent bonds. Yang et al. meanwhile fabricated composite films made of bacterial cellulose and collagen were created using BC hydrogel membranes as the base and Collagen (COL) (type I collagen) extracted from fish bone as the reinforcement.^151^ Cross-linking agents Glutaraldehyde (GT) and 1-ethyl-3-(3-(dimethylamino)­propyl) carbodiimide hydrochloride (EDCHCl) were used to prepare the films. The thermal and mechanical properties of the composite films were investigated, showing a 57.9 and 70.8% improvement in tensile strength compared to BC. This technique, cross-linked BNC has been utilized in the fabrication of flexible electronic circuits and wearable sensors, where its resilience is of utmost importance.

Carbon nanoparticles integration has been found to be particularly successful for applications that necessitate both mechanical strength and flexibility.^152^ Fang et al, developed a novel composite membrane combining bacterial cellulose (BC) and graphene oxide (GO) was developed, achieving enhanced mechanical properties and water stability essential for separation applications. The BC network serves as a porous scaffold, uniformly dispersing GO sheets, resulting in a membrane with superior tensile strength (up to 64.5 MPa, over twice that of pure BC or GO) and improved elongation due to strong hydrogen bonding and effective load transfer. The membrane was prepared by dispersing BC in formamide to create a stable micronetwork, mixing it with GO in an aqueous solution, and filtering the mixture to form a freestanding, flexible composite membrane.^119^

In Si et al. study, Graphene oxide–bacterial cellulose (GO/BC) nanocomposite hydrogels were developed using a one-step in situ biosynthesis by adding a GO suspension into the bacterial cellulose culture medium. This method ensures homogeneous dispersion of GO within the BC matrix, sustaining its 3D porous structure and enabling efficient load transfer between components. The incorporation of 0.48 wt % GO significantly enhances the tensile strength and Young’s modulus by 38% and 120%, respectively, without compromising the hydrogel’s elongation, highlighting its potential for tissue engineering applications.^153^ These findings highlight the enhanced mechanical performance and preparation efficiency of the modified composites. Researchers can enhance the strength and electrical conductivity of materials by incorporating carbon nanoparticles into the BNC matrix. CNT-BNC composites provide a set of characteristics that make them highly suitable for applications in flexible bioelectronics, such as stretchable sensors or bendable displays.

Furthermore, the use of nanoclay can be employed to enhance the mechanical characteristics of BNC, alongside carbon nanotubes (CNTs). For instance, Wei et al. on their study developed a 3D-printable nanocomposite hydrogel ink composed of TEMPO-oxidized bacterial cellulose (TOBC), sodium alginate (SA), and laponite nanoclay (Xls), offering enhanced structural stability and sustained protein release. The hydrogel maintained its stability for over 14 days in PBS, supported cell proliferation with optimal Xls content (<0.5%), and demonstrated potential for carrying and releasing growth factors for tissue repair. These properties highlight its applicability in drug delivery, biomedical devices, and tissue engineering.^154^ Montmorillonite, a type of nanoclay, can be incorporated into the cellulose matrix to enhance its structural stability. This is especially advantageous in situations where BNC must retain its form and durability when subjected to mechanical strain, such as in wearable devices or flexible batteries. For example, Menegasso et al. study^155^ developed a bacterial cellulose hydrogel (BCH) incorporated with montmorillonite (MMT) using a deposition ex-situ method, followed by hydrogel formulation involving homogenization, sieving, and the addition of hydroxyethyl cellulose and propylene glycol.

The composite hydrogel (BCH-MMT) demonstrated enhanced mechanical properties, with tensile strength improving from 30 MPa (pure BC) to 38.5 MPa and a slightly higher Young’s modulus (7.08 MPa vs 6.7 MPa for pure BC), attributed to hydrogen bonding and van der Waals interactions between MMT particles and the BC network.

The BCH-MMT also exhibited significant wound healing potential, including reduced inflammation, complete re-epithelialization, and tissue regeneration in a mouse pressure injury model, suggesting its promise as a novel dressing material for pressure injuries and skin tissue engineering.

To date, as shown in Figure 5a–g, this highlights various functionalization techniques employed to enhance the mechanical properties of bacterial nanocellulose. As shown in Figure 5a, 3D-printed BC-based hydrogel constructs demonstrate excellent structural fidelity and adaptability, making them promising platforms for complex biomedical and engineering applications.^154^ Beyond hydrogel design, diverse functionalization strategies have been developed to enhance the mechanical performance of bacterial nanocellulose (BNC). Cross-linking with glyoxal generates stable hemiacetal and acetal linkages, improving resistance to dissolution and reducing delamination, as illustrated by SEM images of dry and wet glyoxalized BC networks, Figure 5b–c.^149^ Although thermal treatment slightly decreases Young’s modulus, these modifications improve fracture resistance and structural integrity. Covalent bonding through EDC-HCl with collagen (COL) and oxidized BC further enhances tensile strength and elongation at break, owing to the triple-helix flexibility of collagen, as shown in the SEM image of BC/Col/TGGS15, Figure 5d.^150^ Likewise, GO nanosheets, tightly bound to BC via in situ biosynthesis, markedly reinforce mechanical performance, increasing tensile strength and modulus by 38% and 120%, respectively Figure 5e.^153^ Additional reinforcement via montmorillonite (MMT) introduces interfacial hydrogen bonding that further strengthens BC networks Figure 5f.^155^ Finally, stress–strain curves of BC, oxBC, and their collagen composites demonstrate the substantial improvement in strength and flexibility provided by covalent COL integration, Figure 5g.^150^ These strategies highlight how chemical cross-linking, biopolymer incorporation, and nanomaterial reinforcement yield robust BNC composites suited for structural biomaterials and tissue engineering.

**Figure 5:**
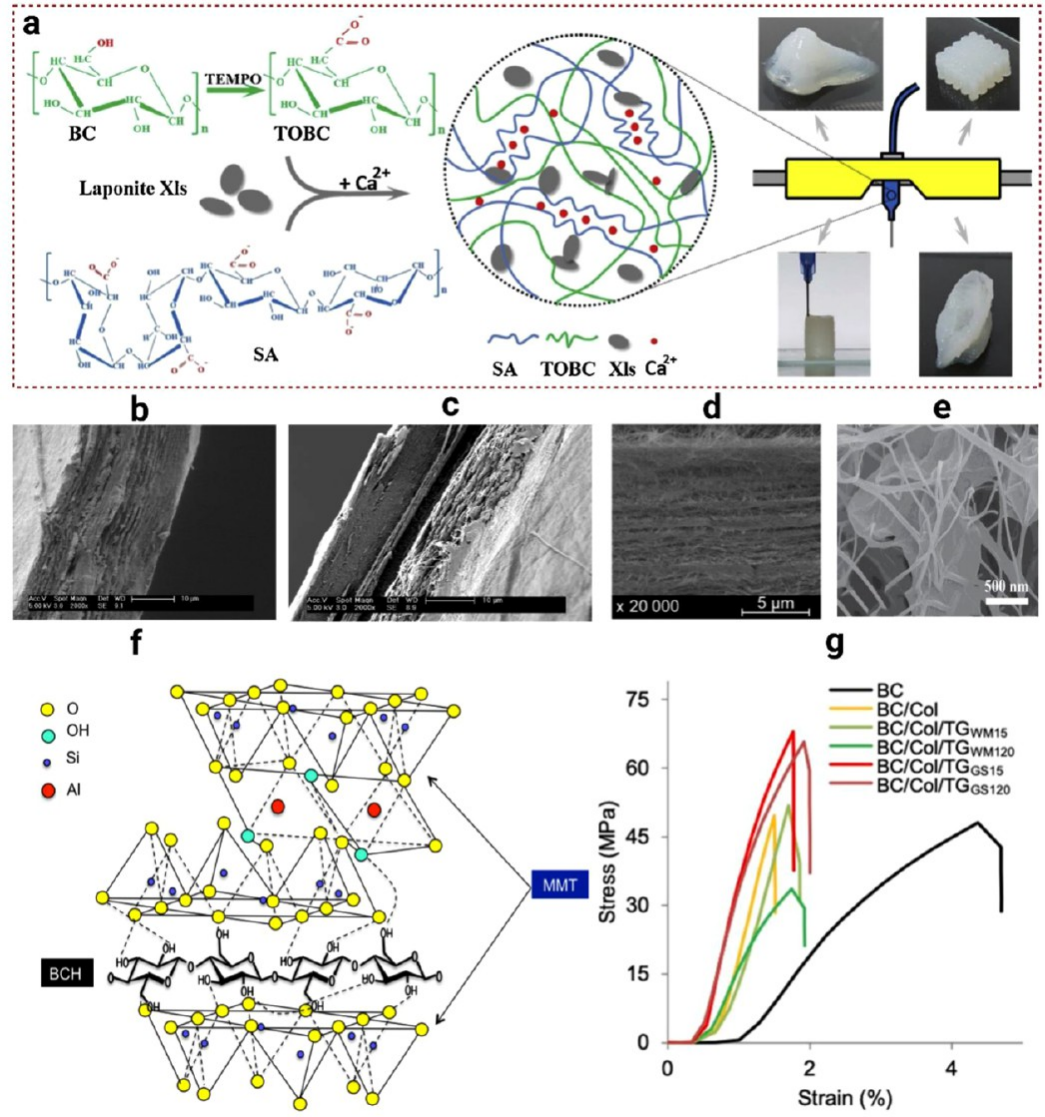
Schematic illustration of the hydrogel fabrication process (a). Reproduced with permission from ref.^154^. Copyright 2020 Elsevier. Scanning electron microscope images of fracture surfaces for dry glyoxalized BC networks (b) and wet glyoxalized BC networks (c). Reproduced with permission from ref.^149^. Copyright 2010 American Chemical Society. SEM image of BC/Col/TGGS15 (d). Reproduced with permission from ref.^150^. Available under a CC-BY license. Copyright 2021 Sommer, A., et al. MDPI. SEM image GO/BC nanocomposite (e). Reproduced with permission from ref.^153^. Copyright 2014 Wiley. Illustration scheme of structure and possible interaction between MMT and BCH (f). Reproduced with permission from ref.^155^. Copyright 2022 Elsevier. Stress–strain curves of BC, oxBC, and their composites with Col (g). Reproduced with permission from ref.^150^. Available under a CC-BY license. Copyright 2021 Sommer, A. et al., MDPI.

The modification of bacterial nanocellulose (BNC) for intelligent bioelectronics presents a promising pathway for creating novel materials that bridge biology and electronics. Researchers are advancing BNC-based devices by enhancing key properties such as electrical conductivity, optical responsiveness, biocompatibility, and mechanical robustness as compiled in Table 3. These functionalization map directly onto device applications: conductive fillers and carbonized structures enable high-performance biosensors and energy-storage components; quantum dots and plasmonic nanoparticles support optical biosensing and neural interfacing; enzyme, peptide, and ionic liquid modifications enhance biocompatibility for tissue scaffolds and implantable platforms, while cross-linkers and nanofillers reinforce mechanical strength for load bearing bioelectronic implants. These strategies establish BNC as a versatile and sustainable platform capable of powering diverse bioelectronic technologies, from flexible sensors to neural interfaces and tissue-engineering scaffolds. With ongoing advancements, the integration of functionalized BNC into next-generation bioelectronics appears increasingly promising.

**Table 3:** List of Recent Published Works in Bacterial Nanocellulose Functionalizations

**Functionalization**	**Method**	**Enhanced Properties**	**Materials**	**Results**	**Reference**
Pyrrole Integration	Electrostatic assembly	Electrical Conductivity; Mechanical Property	Pyrrole; Iron(III) chloride hexahydrate; Ammonium persulfate; G. hansenii, ATC 558232	Conductivity: 0.01–1.2 S/cm;	^116^
				Strength: 40 MPa	
	Polymerization	Adsorption capacity	Nata de coco; Pyrrole monomer; potassium dichromate; 1,5-diphenylcarbazide; Ammonium persulfate; Anhydrous ferric chloride	Adsorption: 372.8 and 555.6 mg.g^-1^ at 298 K	^117^
	Capacitive dressing	Antimicrobial Efficacy	Pyrrole; p-Toluenesulfonic acid; Ferric chloride; G.xylinus (ATCC53582); Carbon cloth fibers	Capacitive dressing acquired a high-efficiency and enduring bactericidal capability	^118^
Graphene Oxide Integration	Layer-by-Layer (LbL) assembly	Mechanical Property	Komagataeibacter xylinus X-2; graphene suspension (purity ≥ 99.4 wt %)	91% improvement in tensile strength and a 279% improvement in tensile modulus	^120^
	Graphene Oxide integration	Electrical Conductivity; Mechanical Property	Komagataeibacter xylinus X-2; graphene suspension (purity ≥ 99.4 wt %)	Conductivity: 70–101 S/m	^121^
				Tensile strength: 218 MPa and modulus at 7.4 GPa	
	Doping Ammonium Iodide	Electrical Conductivity	*Gluconacetobacter xylinus*; Graphene oxide platelets of 300–800 nm width; NH4I suspensions; Hydrazine solution	Conductivity: 1.32 × 10^–4^ S/cm	^122^
	Hydrazine treatment	Electrical Conductivity; Mechanical Property	*Gluconacetobacter xylinus*; Graphene oxide platelets of 300–800 nm width; Hydrazine solution	Conductivity: 12 S/m Tensile Strength: 70–184 MPa Elastic Modulus: 10–13 GPa	^117^
	Vacuum-assisted self-assembly	Electrical Conductivity; Mechanical Property	Bacterial Cellulose; Aqueous Graphene oxide; Hydrazine solution	>1.1 × 10^–4^ S/m	^110^
				> Tensile Strength: 230–242 MPa;	
				> Young Modulus: 1.7–1.8 GPa	
Silver Nanoparticles Integration	Vacuum-assisted filtration	Surface modification to detect pesticide	Acetobacter xylinum ATCC 23769; Silver nitrate (purity ≥ 99.8%); trisodium citrate solution;	Flexible Surface-enhanced Raman scattering substrate.	^123^
				Detection of methomyl pesticide at Raman bands: 674. 940, 1303, and 1420 cm^–1^	
	Modified step-by-step in situ biosynthesis method	Electromagnetic interference shielding	AgNW; poly(vinylpyrrolidone); Komagataeibacter xylinus X-2	Electrical conductivity of 608 365 S m^–1^;	^124^
				EMI shielding effectiveness of 6400 dB mm^–1^	
Carbonization	Water–alcohol solvent exchange	Discharge capacity	Bacterial cellulose; Methanol; ethanol; 1-propanol; 1-butanol; 1-hexanol; 1-octanol; Ether; Acetone; Tetraethylene glycol dimethyl ether ether; *N*-methylpyrrolidone; lithium triflate; bis(2-methoxy ethyl) ether (Diethylene glycol dimethylether); polyvinylidene fluoride(PVDF); carbon black (Super P, Timcal); lithium foil; carbon paper; glass fiber filter	Carbon Yield: 9–14%	^125^
				Discharge capacity: 5.6 mA h cm^–2^)	
				Surface Area: up to 237.42 m^2^g^–1^	
	Sequential imidization	Specific Capacitance Absorption Capacity	Bacterial cellulose pellicles (30” 40 cm^2); pyromellitic dianhydride (PMDA), triethylamine (TEA, 98%),N,N-dimethylacetamide (DMAc), cyclohexane, ethanol, acetone,N,N-dimethylformamide (DMF), benzylalcohol, hexane, heptanes, isopropyl alcohol, octadecylene, toluene and chloroform; pump oil and colza oil	Specific capacitance of 194.7 F g^–1^ and excellent stability.	^127^
				Absorption capacity: 10–40 g/g	
Fluorescent Probes Integration	Soaking	Paper Acidity	Gluconacetobacter xylinus; Cadmiumtelluride (CdTe) quantum dots; Rhodamine B	Strong Photoluminescence intensity response to pH levels ranging from 3.0 to 7.5	^131^
	Immobilization	Heavy Metals detection	Gluconacetobacter xylinus; CdTe quantum dots (QDs; dithizone (DTZ)	Mercury sensing under optimal conditions with a limit of detection (LOD) of 1 nM	^132^
	Nanonetwork embedding	Optical sensing	A. xylinum; Silver and gold nanoparticles; CdSe@ZnS quantum dot; NaYF 4:Yb^3+@Er^3+&SiO 2 nanoparticles	Crystallinity (82%) and crystallite size (6.3 nm); average tensile strength (345 MPa), Young’s modulus (17.3 GPa) and strain-at-break (7%); selectivity toward thiourea and cyanide as sensing platform; 34–57% quenching as a Photoluminescent Platform; lower optical density and a noisy spectra as a Preconcentration Platform	^133^
	Plasmonic coupling	UV radiation detection	Bacterial cellulose nanopaper; silver and gold nanoparticles	Potential preventive healthcare tool against threats derived from uncontrolled UV exposure via wearable/adhesive devices, particularly to avoid either UV-induced skin damage or inadvertently consumption of hazardous agents released from bottled beverages upon UV exposure	^134^
Enzymes Immobilization	Solution Casting	Glucose Detection in Blood	Bacterial cellulose nanofibers; Gold nanoparticles; glucose oxidase (GOx) and horseradish peroxidase (HRP)	Detection limit for glucose in optimized conditions was as low as 2.3 mM with a linear range from 10 mM to 400 mM.	^137,138^
	In Situ Chemical reduction	Dopamine detection	Gluconacetobacter xylinum; Laccase; Nafion; Palladium chloride; dopamine	Excellent electrocatalysis toward dopamine with high sensitivity (38.4 μA.mM^-1^), low detection limit (1.26 μM), and wide linear range (5–167 μM)	^139^
	In Situ Chemical reduction	Hydroquinone detection	Gluconacetobacter xylinum; Laccase; Nafion; Palladium chloride; dopamine	Excellent electrocatalysis toward dopamine with low detection limit (5.71 nM), and wide linear range (30–100 nM	^140^
	Heterofunctional Absorption	Bioactive dressing	Komagataeibacter hansenii; papain; sodium periodate	Membranes showed an anomalous and non-Fickian release mechanism; e wet OxBC-Papain membrane showed the highest percentage of diffusion of active papain	^141^
	Physical Absorption	Antimicrobial Activity	Bacterial cellulose nanofibers; Lysozyme enzyme	Most inhibiting effect against *A. niger* and *S. cerevisiae*	^142^

## 4 Smart Integration for Bioelectronics

### 4.1 Biosensors

Biosensors are analytical tools or devices that provide quantitative biological information through a biological recognition element such as enzymes, proteins, antibodies, microorganisms or DNA that is in direct or spatial contact with a physicochemical transducer element. This transducer element can convert the biological response into a detectable signal, which may involve chemical, electrical, or optical methods.^140,156–158^ Signal conditioning to become clinical metrics follows this pathway from raw potential signals (ΔE in mV) → dose–response curves and calibration models → clinical diagnostic metrics (positive/negative, viral load correlation, LOD/LOQ)^156^ while for raw Raman spectra the data conditioning process follows from raw data → baseline correction → peak intensity extraction → calibration curves → clinical metrics (LOD, recovery, cancer vs normal).^158^

As hybrid devices, biosensors are crucial for achieving rapid and selective quantitative or semiquantitative analysis compared to conventional detection methods like chromatography and spectroscopy, which are typically expensive and time-consuming. Moreover, biosensors are cost-effective, be able to detect real-time, do not require extensive instrumentation, and offer relatively fast response times due to their chemical redox or optical responses.^25,158^

Bacterial nanocellulose (BNC) holds great promise as a biosensor material due to its biocompatibility, substantial water-holding capacity, high surface area, and mechanical strength. BNC has a hydrophilic nature that enhances biomolecule adsorption on its surface. This hydrophilicity, along with BNC’s high surface area and nanoscale pore structure, provides numerous anchoring points for biomolecule immobilization on transducer surfaces, allowing efficient transport of biomolecules to the sensor surface and increasing binding affinity.^26,159^ These features lead to enhanced sensitivity, making BNC suitable for developing customized biosensors for health monitoring, including wearable and implantable devices. Furthermore, BNC has higher purity and degradability compared to petroleum-based and plant-based materials used in biosensors, such as poly­(methyl methacrylate), polyethylene terephthalate, polydimethylsiloxane, and polyurethane, which are difficult to degrade.^160^ Additionally, due to the zero content of hemicellulose and lignin, BNC has higher purity than cellulose nanofibers and cellulose nanocrystals extracted from plant fibers, which involve complex purification steps.^161,162^ However, there is a need to enhance conductivity, functionalization and sensitivity of BNC for its use in electrochemical and optical biosensing. To overcome this, BNC can be combined with functional materials such as graphene oxide, hydrogel, carbon nanotubes (CNT), conductive polymers, zinc, silver (Ag), gold (Au) nanoparticles (NPs) and boron nitride to form functionalized nanocomposites and nanostructures.^156,159,162,163^ These combinations enhance conductivity, improve sensitivity and functionalization of BNC, making versatile and effective material for various biosensing applications like interdigitated electrode screen-printed on bacterial nanocellulose. Introducing NPs enhances the electronic and optical transduction characteristics of biosensors. The interaction of biomolecules and NPs on polymer matrices, like BNC, can be facilitated through physical, chemical, or biological methods.^168,169^ CNTs and graphene, known for their high surface area, electrical conductivity, and stability, are also used in BNC-based material manufacturing.^152,156^ Even small amounts of CNTs can significantly improve the mechanical and thermal properties of polymer composites.^152^

Electrochemical systems are among the most frequently used transducers in commercial biosensors for converting biochemical signals into measurable outputs. They operate by detecting redox reactions when a bioreceptor selectively interacts with an analyte in solution, generating an electrochemical signal.^140,165^ This signal can produce various responses, including amperometric, potentiometric, field-effect transistors, and conductometric outputs.

Progress in biosensing analysis necessitates a deep understanding of charge transport and electron transfer at the electrode/electrolyte interface, which ensures a robust bond between the bioreceptor and target analyte, thereby immobilizing the biomolecule on the transducer.^24,25^ To enhance the quantitative accuracy, selectivity, and reactivity of these techniques, the physicochemical properties of the transducer interface can be modified by BNC-based materials. Constructing a modified layer on the transducer’s surface is particularly desirable as it helps retain specific biological functions. Designing an appropriate surface support for the electrode/electrolyte interface, tailored to the

biomolecule’s properties and stability, is one way to optimize electron transfer and ensure effective biomolecule immobilization. Enhancing the integration between the biomolecule and the electrode surface results in higher biosensor sensitivity.^25^

Conducting polymers such as polyaniline and polypyrrole are often used to prepare BNC-based materials, combining their conductive properties with the porous structure of nanocellulose to create superior sensing characteristics.^170,171^ BNC’s excellent water-holding capacity and hydrophilic properties enhance the physical and structural properties of these conducting polymers.^25^ Consequently, these polymers improve the transduced analytical signal by facilitating interactions between immobilized biomolecules and the target analyte.^19^ Despite these advantages, the application of conducting polymers has been limited due to their low solubility in common solvents and poor mechanical properties.^25,172^

To modify the electrode interface and enhance the immobilizing the biomolecule on the transducer, the use of BNC nanocomposites with various NPs, such as Au, and Zinc were studied.^140,159^ Zhang and colleagues utilized a glassy carbon electrode (GCE) as the transducer, applying the Au-BNC nanocomposites to its surface to provide a suitable environment for immobilizing horseradish peroxidase (HRP).^173^ In this process, BNC are uniformly coated with AuNPs in an aqueous suspension using poly­(ethylenimine) (PEI) as both the reducing and linking agent. The incorporation of different halides results in Au-BNC nanocomposites with varying Au shell thicknesses, with PEI playing a crucial role in the formation mechanism. A novel H_2_O_2_ biosensor is developed using these Au-BNC nanocomposites, providing excellent support for HRP immobilization and achieving a limit of detection (LOD) below 1 μM.^174^

Li et al. used BNC-Au nanocomposites to immobilize the laccase enzyme on a GCE, creating a biosensor for hydroquinone detection.^140^ The BNC-Au nanocomposites, formed through vacuum filtration of AuNPs onto BNC, showed effective electron transfer between the electrode surface and the enzyme’s electroactive center. This biosensor demonstrated a satisfactory electrochemical response, verifying the significant surface area and nanostructure of BNC in supporting biomolecule immobilization with LOD of 5.71 nM.^140^ In another study, a novel composite electrode material, BNC-Zinc-BIM (benzimidazole), was developed. BNC served as a conductive medium in the composite, facilitating charge transport, which was investigated through density functional theory calculations.^159^

The composite was applied in the electrochemical detection of Bisphenol A, achieving a LOD of 12 nM and a sample recovery rate of 95.1%–105.6%. The study revealed that the oxygen-containing groups in BNC and nitrogen-containing heterocycles in BIM tend to lose electrons, while zinc ions actively acquire electrons, promoting charge transfer and enhancing the composite’s electrocatalytic properties.^159^ The functionalized BNC could serve as a substrate to build an optical biosensor. Recent study presents a biomimetic approach using polymer functionalized BNC to construct optical biosensors, leveraging the biocompatibility, mechanical strength, and modifiable surface area of BNC.^24^

By functionalizing BNC with polydopamine (PDA), which enhances adhesion and provides a black background, a molecularly imprinted photonic sensing layer was created. The resulting biosensor, utilizing PDA as both the substrate modifier and molecularly imprinted polymer for lysozyme detection, achieved a LOD of 0.8 nmol/L in spiked human serum and demonstrated selectivity against cystatin C.^24^

As BNC, it is not only provides opportunities for functionalization and immobilization but also shows a reduced optical response, especially in surface-enhanced Raman spectroscopy (SERS), along with lower background noise and fewer interfering signals during detection.^158,166^ Additionally, BNC can facilitate the synthesis of plasmonic NPs, such as Au and Ag, with diverse sizes and shapes. Utilizing these characteristics, BNC is an advantageous and flexible substrate for NP loading, offering considerable potential in various optical sensing applications.^158,166,174^

In this regard, a recent study successfully developed a two-dimensional BNC nanogold substrate for SERS-based biosensor via in situ reduction and employed a sandwich model with 4-MBN as a Raman reporter for the sensitive detection of TNF-α.^166^ This cytokine, which plays a critical role in various diseases, was detectable over a wide range between 10^–8^ to 10^–4^ mg/mL with an impressive LOD of 0.35 pg/mL. The assay demonstrated exceptional specificity, with a 98% recovery rate for serum samples at 5 × 10^–7^ mg/mL.^166^ Another recent study developed an in situ reduced BNC membrane for creating a 3D flexible SERS substrate.^158^ Ag NPs were uniformly distributed on the BNC membrane nanofibers through a controlled reaction, resulting in highly stable and uniform hot spots. The optimized Ag NPs-BNC membrane substrate demonstrated high SERS activity with an enhancement factor of 5.3 × 10^8^ and a relative standard deviations (RSD) of 11.0%, maintaining performance even after 45 days. This substrate was used to detect glutathione (GSH), in serum samples, distinguishing GSH levels in cancerous versus normal samples.^158^

BNC hydrogel serves as an excellent substrate and matrix for sensor preparation owing to its beneficial characteristics such as biocompatibility, ease of processing, high water retention capability, and attractive mechanical properties.^162^ By integrating BNC within in-situ generated NP, such as AuNPs, novel composite hydrogels can be developed. These composite hydrogels combine the advantageous properties of BNC and the enhanced ionization efficiency provided by AuNPs, leading to highly sensitive biosensors capable of detecting biomarkers.^175^ Researchers developed a BNC/carboxymethyl cellulose (CMC)/AuNPs composite hydrogel by soaking a BNC membrane in CMC solution, followed by embedding AuNPs through in situ chemical reduction.^175^ The composite hydrogel retained the physical and chemical properties of BNC, with uniformly distributed AuNPs of about 13 nm. This hydrogel was used for the selective adsorption of GSH and detected using laser desorption/ionization mass spectrometry. The hydrogel provided a linear detection range of 50–10,000 nM and a LOD of 54.1 nM. The AuNPs acted as a self-matrix, and the hydrogel effectively preconcentrated GSH, enabling ultrasensitive detection necessary for disease diagnosis.^175^ In an interesting study, researchers highlighted the pivotal role of self-powered sensors in the advancement of wearable electronics, examining the potential of eco-friendly ionic thermoelectric hydrogels based on carboxylated BNC to harness mild heat from human skin for continuous electricity generation.^176^ They developed a TEMPO-oxidized carboxylated BNC coordination double-network ionic thermoelectric hydrogel, incorporating lithium bis­(trifluoromethane) sulfonamide as an ion provider for thermos-diffusion for enhanced thermoelectric properties. This BNC hydrogel demonstrated a high-power output of 538 nW at a 20 K temperature difference and excellent mechanical properties, providing a viable solution for self-powered sensor design and application.^176^

Figure 6 highlights representative strategies for tailoring bacterial nanocellulose (BNC) toward flexible and sustainable biosensing platforms. A stepwise fabrication route demonstrates how *Gluconacetobacter hansenii*–derived BC can be processed into biodegradable electrochemical devices: after incubation and purification (i–iii), the BC sheets were screen-printed with carbon and Ag/AgCl inks (iv) to form three-electrode configurations, subsequently cut and assembled into portable, low-cost sensors (v–vi), here exemplified in SARS-CoV-2 detection, Figure 6a.^156^ Complementary approaches further expand BNC versatility, such as the preparation of NBC–NBN composite membranes by vacuum-assisted filtration, Figure 6b,^167^ which enhances structural and functional performance, and the development of ionic thermoelectric hydrogels Figure 6c,^176^ illustrating chemical functionalization routes that impart advanced energy-harvesting and sensing capabilities. Together, these examples emphasize the adaptability of BNC substrates through physical, chemical, and compositional modifications, underlining their promise as sustainable building blocks for next-generation biosensor technologies.

**Figure 6:**
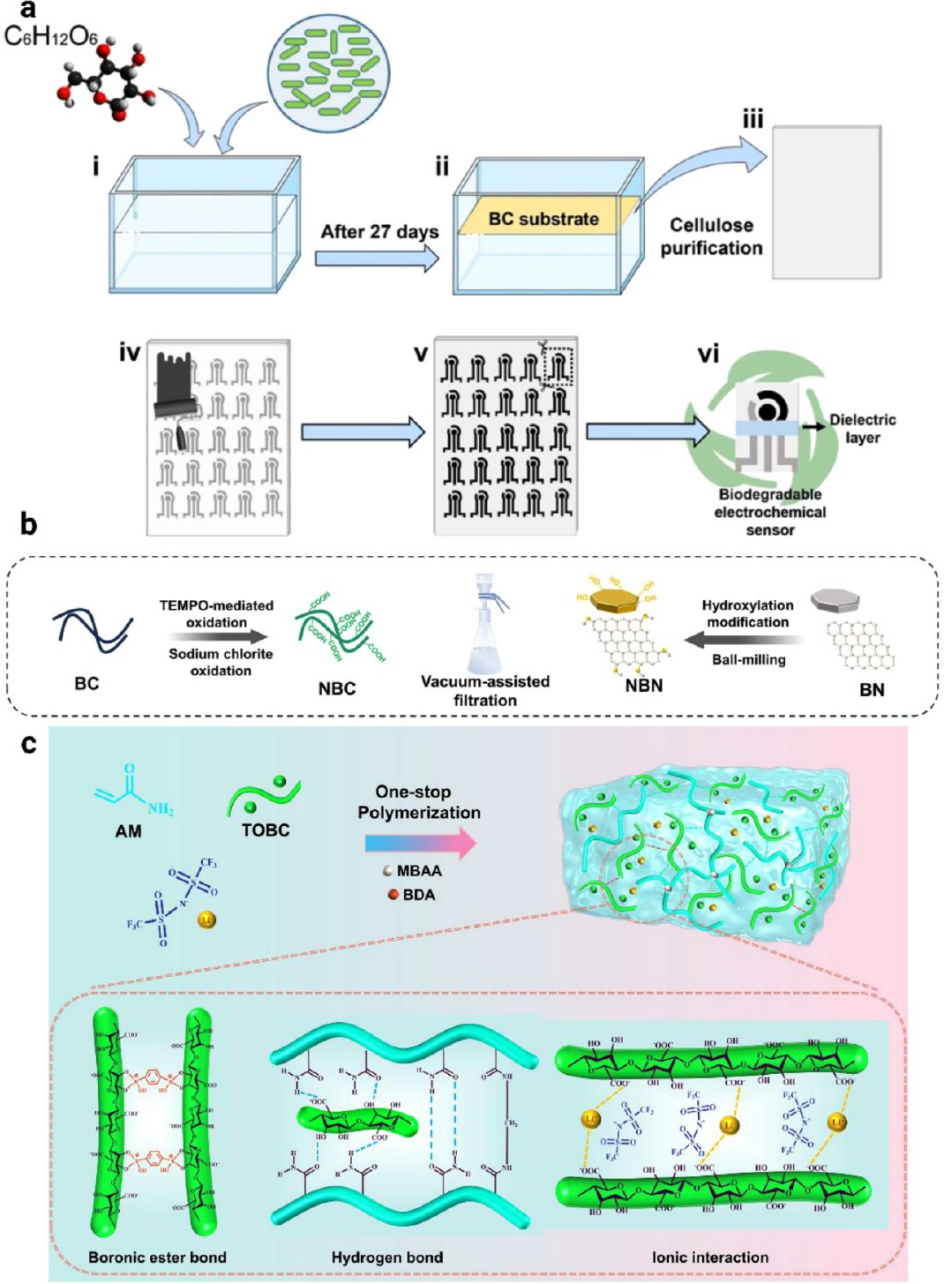
Fabrication steps of the biodegradable BC substrate and the electrochemical devices. First, the bacterium *Gluconacetobacter hansenii* was incubated in HS medium with 20 g L^–1^ glucose (i); after 27 days, a BC substrate was collected and treated with 5 mmol L^–1^ NaOH at 80C (ii), resulting in a clear sheet (iii). Next, the biodegradable BC substrate was screen printed with carbon and Ag/AgCl conductive ink (iv), resulting in a device with three electrodes (WE, CE, and RE), which were cut out using a scissor (v), yielding a portable, biodegradable, and inexpensive electrochemical sensor (vi) (a). Reproduced with permission from ref.^156^. Copyright 2023 Elsevier. Preparation of NBC-NBN composite membranes by a vacuum-assisted filtration method (b). Reproduced with permission from ref.^167^. Copyright 2024 American Chemical Society. Schematic of the fabrication process and chemical structure for the ionic thermoelectric hydrogel (c). Reproduced with permission from ref.^176^. Copyright 2023 Elsevier.

Functionalization with polyethylenimine (PEI) and carbon-modified multiwalled carbon nanotubes (c-MWCNTs) enabled phage immobilization for selective bacterial sensing via electrostatic interactions. Further surface oxidation enhanced charge density in BC/BN composites, improving ion transport for energy conversion applications, while BC substrates served as scaffolds for interdigitated electrodes modified with bioactive layers like chitosan and chondroitin sulfate for biomarker detection. Incorporation of metal oxides (Ag, CuO, Cu_2_O) into BC-based carbon paste electrodes enhanced their catalytic activity for small-molecule detection, and polydopamine (PDA) coatings on BC enabled optical biosensing with molecular imprinting. A PVAN interlayer allowed strong grafting of polyaniline (PANI) to BC fibers, improving electrical conductivity and enabling bioelectronic interfacing. BC also supported SERS detection through AgNP loading and aptamer immobilization for sensitive toxin identification, and was utilized in ionic thermoelectric hydrogels, where carboxylated TOBC formed robust, conductive double-network structures. Lastly, in situ incorporation of AuNPs into BC and BC/CMC hydrogels demonstrated tunable plasmonic properties for enhanced signal transduction. Collectively, these functionalization approaches highlight BC’s unique versatility in achieving advanced conductivity, specificity, and integration within next-generation biosensor systems.

Meanwhile, Figure 7 illustrates significant outcomes of bacterial nanocellulose (BNC) functionalization for biosensor development. Cross-reactivity assays confirmed reliable SARS-CoV-2 variant detection, where negative and positive clinical samples were clearly differentiated with a defined cutoff response, Figure 7a.^156^ Environmental sensing was demonstrated through GO–BC films, with the humidity response curve of 20BG-x-60 indicating stable and sensitive performance, Figure 7b.^164^ Biomedical applications were also advanced. Capacitance spectra of screen-printed BNC electrodes enabled label-free p53 detection, with responses scaling to concentration Figure 7c.^163^ Enhanced electrochemical activity was achieved using AgNP/Cu_2_O/CuO-modified BNC electrodes, as confirmed by FE-SEM surface analysis Figure 7d.^168^ Polymer integration further improved conductivity, with FIB/SEM images of PVAN/PANI grafted BC showing uniform coatings without compromising cytocompatibility, Figure 7e.^171^ Additional modifications extended versatility: PDA-coated BNC membranes produced flexible black films for colorimetric detection, Figure 7f,^24^ AgNP@BC membranes enhanced optical activity, Figure 7g,^174^ and ionic thermoelectric BNC hydrogels provided tunable properties for wearable sensors Figure 7h.^176^ These strategies demonstrate BNC’s adaptability as a sustainable, biocompatible, and multifunctional platform for next-generation biosensors.

**Figure 7:**
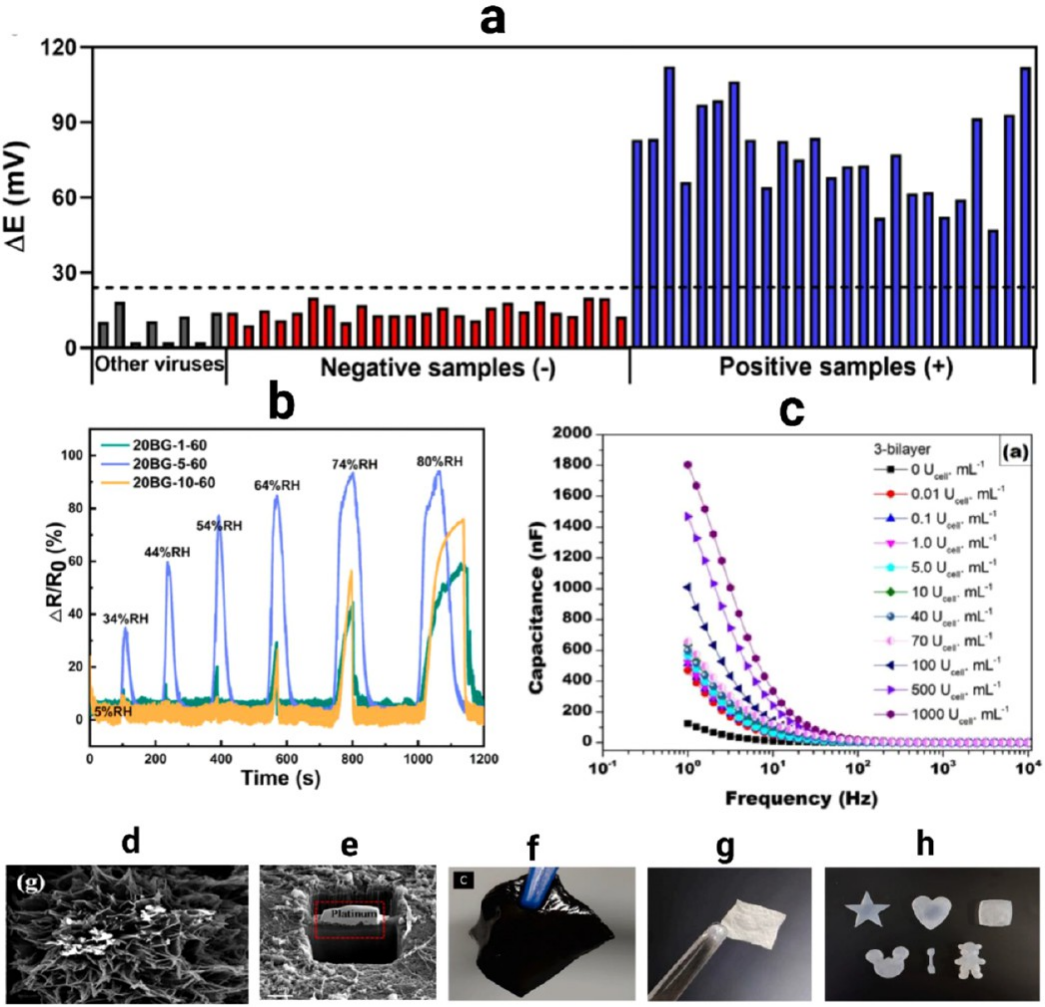
Comparison of the electrochemical response obtained by the cross-reactivity studies (gray bars), 25 SARS-CoV-2-negative clinical samples (red bars), and 25 positive SARS-CoV-2 clinical samples containing different lineages (blue bars). The dotted line indicates the cutoff value of ΔE­(V) (ΔE­(V) = E_sample_ - E_blank_) response established to indicate whether the sample was positive for SARS-CoV-2 variants as determined by our biosensor (a). Reproduced with permission from ref.^156^. Copyright 2023 Elsevier. Humidity sensing performance curve of 20BG-x-60 (b). Reproduced d with permission from ref.^164^. Copyright 2024 Elsevier. Capacitance spectra of screen-printed biosensors in bacterial nanocellulose substrate of cell lysates at different p53 concentrations (c). Reproduced with permission from ref.^163^. Copyright 2022 Elsevier. FE-SEM picture of Ag NPs/Cu2O/CuO/BNC/CPE (d). Reproduced with permission from ref.^168^. Copyright 2023 Royal Society of Chemistry. FIB/SEM images illustrating the FIB lift-out process used to extract a thin film TEM sample from the bulk BC/PVAN/PANI (e). Reproduced with permission from ref^171^. Copyright 2019 Elsevier. Photograph of BNC with PDA, resulting in black membranes in wet (f). Reproduced with permission from ref.^24^. Copyright 2023 Elsevier. Digital image of Ag NPs @ BCM (g). Reproduced with permission from ref.^174^. Copyright 2023 Elsevier. Schematic diagram of various BNC hydrogels (h). Reproduced with permission from ref.^176^. Copyright 2023 Elsevier.

BNC-based biosensors generally perform within the same range or better in terms of linear range and LOD compared to other alternatives. However, their stability, repeatability, and reproducibility show mixed results, with both lower and higher RSD compared to other options.^25^ In terms of the selection of functional materials, no specific rules or general methods exist for predicting biosensor performance. The performance depends on several factors, including the properties of the target analytes, biorecognition entities, material features, synergistic effects from functionalization materials and methods, and test protocols. Thus, many aspects of biosensor research involving BNC remain unexplored.

### 4.2 Neural Interfaces

The development of neural interfaces in smart bioelectronics is a rapidly growing field aimed at advancing our ability to monitor, stimulate, or modulate nerve tissues using bioelectronic devices.^177^ Among the many materials being explored for these interfaces, bacterial nanocellulose has emerged as a highly promising candidate due to its unique properties, such as biocompatibility, flexibility, and the ability to function.^178^ For instance, Yang et al, study presents supersoft multichannel electrodes by layering gold on bacterial nanocellulose (Au-BNC), which has mechanical properties closely matching biological tissues.^179^ These durable and flexible electrodes demonstrated excellent conductivity and in vivo brain activity recording potential, pioneering the use of naturally derived cellulose for neural interfaces and opening avenues for broader biomedical applications like electromyography and electroencephalography sensors. Bacterial nanocellulose is increasingly used as a coating material for neural electrodes, significantly enhancing the biocompatibility of these devices.

One of the main challenges with traditional materials is their tendency to cause inflammation and rejection when implanted in the body for long periods. The porous structure of BNC mimics the extracellular matrix of neural tissues, promoting better integration with the surrounding biological environment and reducing inflammation.^180^ The coating ensures longer-lasting implants that work harmoniously with the body. In addition to preventing inflammation, it improves the stability and longevity of neural implants, addressing one of the key limitations of existing materials.^181^

Figure 8 highlights recent advanced functionalization techniques of bacterial nanocellulose (BC) for smart integration in neural interface applications. In situ polymerization of PEDOT onto BC nanofibers using FeCl_3_ produced 3D BC/PEDOT nanocomposites with enhanced electroactivity suitable for neural signal transmission, Figure 8a.^182^ The BC/PEDOT/GO film is fabricated via in situ polymerization of EDOT on BC nanofibers assisted by FeCl_3_, forming highly electroactive BC/PEDOT composite nanofibers. GO is then combined onto the BC/PEDOT surface through electrostatic interactions between PEDOT and GO, with the resulting composite characterized by zeta potential measurements at various ratios, Figure 8b.^183^ Similarly, fabrication of Au-BC electrode arrays via electron beam evaporation and precise masking onto hot-pressed BC films enabled the development of flexible, biocompatible, and multichannel bioelectronic devices. Integration of BC with 3D graphene foams (3D-BC/G), fabricated via CVD on nickel scaffolds, resulted in porous nanofiber-embedded structures that preserved graphene’s architecture while improving surface topography and supporting neural stem cell (NSC) attachment and proliferation.

**Figure 8:**
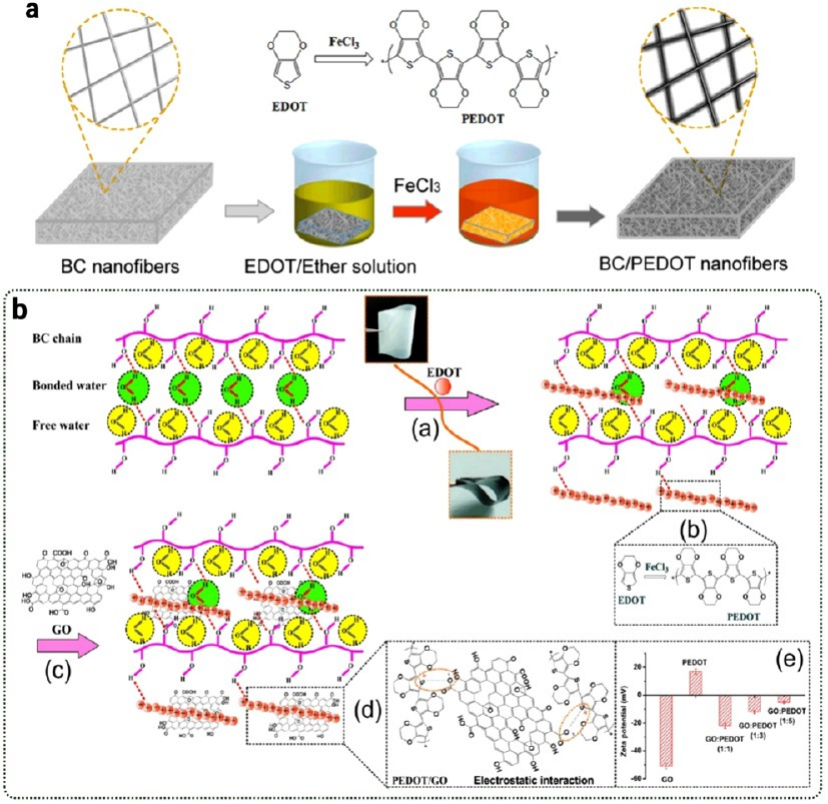
Fabricating 3D BC/PEDOT Nanofibers where the in situ polymerization of EDOT occurs on the surface of BC nanofibers assisted by FeCl_3_, producing 3D BC/PEDOT composite nanofibers with high electroactivity (a). Reproduced with permission from ref.^182^. Copyright 2015 American Chemical Society. The BC/PEDOT/GO film is fabricated via in situ polymerization of EDOT on BC nanofibers assisted by FeCl_3_, forming highly electroactive BC/PEDOT composite nanofibers. GO is then combined onto the BC/PEDOT surface through electrostatic interactions between PEDOT and GO, with the resulting composite characterized by zeta potential measurements at various ratios (b). Reproduced with permission from ref.^183^. Copyright 2016 American Chemical Society.

Furthermore, the functionalization of BC/PEDOT with graphene oxide (GO) through electrostatic binding yielded BC/PEDOT/GO films with improved electrical conductivity and surface charge modulation, promoting stable charge transfer and enhanced scaffold performance. These functionalization strategies demonstrate how BC can be engineered into conductive, porous, and biologically interactive platforms ideal for neural interfacing, offering promising solutions for brain–machine integration and neuroregenerative devices.

In addition, beyond simple coatings, BNC is being developed into multifunctional materials that not only improve biocompatibility but also enhance the electrical performance of neural interfaces. By combining BNC with other conductive materials like PEDOT or graphene oxide, researchers have created hybrid systems that are both flexible and highly conductive. Chen et al, study presents a novel approach to synthesize electroactive and flexible 3D nanostructured biomaterials by coating bacterial cellulose nanofibers with poly­(3,4-ethylenedioxythiophene) (PEDOT) via in situ interfacial polymerization.^182^ The BC/PEDOT composite nanofibers were prepared by freeze-drying BC hydrogel into a sponge-like membrane, followed by in situ interfacial polymerization of EDOT using FeCl_3_ as an oxidizing agent under ultrasonic oscillation. The method enables fine-tuning of coating thickness, electrical conductivity, pore size, and mechanical properties, resulting in nanofibers with excellent electrochemical performance and biocompatibility. The biocompatibility and cytotoxicity of the BC/PEDOT nanofibers were evaluated using PC12 neural cells, which were cultured on the nanofiber substrates and assessed through MTT assays, immunostaining, and fluorescence microscopy. The BC/PEDOT nanofibers exhibit enhanced neural cell response under electrical stimulation, demonstrating potential for applications in biosensing, drug delivery, and implantable electrodes for tissue engineering.

The same group of Chen et al. further studied the synthesis of biocompatible bacterial cellulose (BC) nanofibers coated with poly­(3,4-ethylenedioxythiophene) (PEDOT) and doped with graphene oxide (GO) through in situ interfacial polymerization.^183^ The BC/PEDOT/GO nanofiber films exhibited a conductivity of up to 1.25 S/cm and significantly reduced impedance compared to BC/PEDOT films. The film thickness was controlled by varying EDOT and FeCl_3_ concentrations, while GO doping introduced abundant free carboxyl and hydroxy groups for surface modification. Cytotoxicity and proliferation assays using PC12 neural cells showed high cell viability (>90%) and improved cell alignment and differentiation after electrical stimulation. These properties make composite material promising for applications in biosensing, regenerative medicine, and tissue engineering.

These multifunctional BNC-based materials are particularly useful in neural recording and stimulation, where electrical signals need to be accurately transmitted across the interface. With the electrical enhancements, these devices have shown the ability to both sense and stimulate neural signals, which is essential for applications such as brain-computer interfaces and neural prosthetics.

In neuroprosthetics, a field that combines neuroscience and biomedical engineering to create artificial devices that replace or improve the function of the nervous system, devices that restore or replace the function of a damaged nervous system require highly durable and stable materials. BNC’s unique properties have led to its application in neuroprosthetic devices, where it is integrated into electrodes to improve their long-term performance. For instance, Meng et al. study focuses on the development of ultrasoft, sensitive bacterial cellulose-based fibers for biocompatible strain sensing, specifically addressing the mismatch between bioelectronics and soft tissue.^184^ The experimental approach involved fabricating bacterial cellulose fibers combined with polyaniline (PANI) to create a flexible, highly sensitive strain sensor capable of detecting minute tensile forces, with mechanical properties closely resembling those of biological tissues. The fibers demonstrated excellent performance in monitoring subtle biological motions, such as human pulse and bullfrog heartbeats, and were further configured into multipoint sensors for advanced applications. These characteristics position BNC-based fibers as promising candidates for neuroprosthetic devices, potentially enabling real-time monitoring and enhanced human-machine interfaces. These neuroprosthetics show improved compatibility with neural tissues, making them ideal for long-term implantation. Such advancements have helped develop devices that can better interface with human tissue, ensuring they last longer and function more reliably than traditional prosthetic materials. One of the most exciting applications of BNC in neural interfaces is its use in devices that can simultaneously stimulate and record neural activity.

Previous study from Pértile et al, investigates the functionalization of bacterial cellulose (BC) membranes using recombinant proteins with bioactive peptides, such as IKVAV and RGD, to enhance neuronal and mesenchymal cell adhesion.^185^ The experimental approach involved producing and purifying recombinant proteins fused with a carbohydrate-binding module (CBM3) to stably adsorb onto BC surfaces, followed by cell adhesion, viability assays, and neurotrophin expression analysis. The results showed a significant improvement in PC12 and mesenchymal cell adhesion, as well as increased secretion of nerve growth factor (NGF), indicating potential for nerve tissue regeneration. This modified BC scaffold offers promising applications in neuroprosthetics, where promoting cell adhesion and providing neurotrophic support are critical for neural integration and repair.

Traditional materials often struggle with this dual functionality, but BNC-based materials, enhanced with conductive fillers, have shown great promise. For example, carbon nanomaterial embedded in BNC can provide the necessary conductivity for neural stimulation, while the BNC matrix itself supports the growth and health of neurons.^152^ In the study of Guo et al., the three-dimensional bacterial cell graphene foam (3D-BC/G) prepared by in situ polymerization at the BC interface on the skeleton surface of the porous graphene foam can not only support the growth and adhesion of neural stem cells (NSCs), but also induce NSCs to selectively differentiate into neurons, forming neural network 1 in a short time.^186^ This combination allows for continuous, real-time monitoring of neural signals while providing therapeutic stimulation. It offers a versatile solution that can be used in neural implants, where dual functionality is crucial for both diagnosis and treatment.

Advances in printing technologies have enabled the creation of screen-printed BNC electrodes,^187,188^ which are being explored for their potential in neural applications. These electrodes are flexible, lightweight, and highly biocompatible, making them ideal for use in neural interfaces. In a recent study of Reynolds et al, BNC electrodes were used to monitor surface biopotentials in plant tissues as a model system for biological signal recording.^187^ The success of this research suggests that BNC-based electrodes could soon be used to record electrical activity in the brain or peripheral nerves.

In fact, Wilkins et al. further developed screen-printed bacterial nanocellulose thin film where the Electrode impedance and surface area were characterized using electrochemical impedance spectroscopy, cyclic voltammetry, and chronoamperometry.^188^ Annealing temperature was evaluated for effects on electrode performance. Electrodes annealed at 80 °C had a 25% higher impedance across frequencies in the range 0.01 Hz to 100 kHz and a 6–19% lower ratio of geometric surface area to electrochemically active surface area than electrodes annealed at 60 °C. These screen-printed electrodes offer a cost-effective and versatile solution for large-scale production of neural interfaces, bringing BNC technology closer to widespread use in medical devices.

One of the most significant challenges in developing neural implants is ensuring their long-term compatibility with the body. Chronic neural implants often face problems like inflammation, scarring, and device failure due to material degradation. For instance, in Wilkins et al, although they did not directly assess long-term biodegradation, their impedance data show that structural changes in BNC (e.g., by annealing) increase impedance by 25%. This suggests that during degradation, as the cellulose network fragments and ECSA decreases, impedance would follow a similar upward trajectory—initially stable, then progressively increasing as conductive pathways collapse.^188^ However, studies have shown that BNC-based neural implants can overcome these issues. The natural biocompatibility of BNC reduces the body’s immune response, allowing the implant to function effectively for longer periods. This makes BNC an ideal material for chronic neural implants that need to remain in place for years or even decades. The material’s flexibility and strength further contribute to its success in maintaining long-term stability in neural implants.

In the field of bioelectronics, BNC has gained attention for its role in developing bioelectronic neural interfaces. These devices can monitor neural activity and respond to it by delivering electrical signals or therapeutic compounds. BNC’s flexibility, transparency, and ability to incorporate conductive materials make it an ideal substrate for bioelectronic devices that require intimate contact with neural tissues. These interfaces are being developed for applications in brain-computer interfaces (BCIs), where they can help restore lost motor functions or enable new forms of communication. With its potential to be customized for specific bioelectronic needs, BNC is at the forefront of next-generation neural interfaces. In addition to recording and stimulating neural activity, drug delivery is another important function of neural interfaces.

BNC is being explored as a drug delivery vehicle in neural implants, where it can slowly release anti-inflammatory or neuroprotective drugs directly to the site of the implant. This not only improves the performance of the implant but also reduces the risk of complications like inflammation or infection. Researchers have developed BNC-based drug delivery systems that can be incorporated into neural interfaces, providing both therapeutic benefits and enhanced device longevity.

As research in this area continues to advance, it is likely that BNC will play an increasingly important role in the development of next-generation neural interfaces, helping to improve the quality of life for patients with neurological conditions and paving the way for new forms of bioelectronic communication and therapy. Also, BNC-based electrodes should align with following standards: (1) ISO 10993 requires full biocompatibility testing (cytotoxicity, irritation, systemic and chronic toxicity, hemocompatibility, implantation, degradation); and (2) ISO 14708–3 adds device-level safety tests (thermal rise/ΔT, charge-injection safety, mechanical durability, EMI/MRI compatibility, labeling).

The presents the significant outcomes of functionalized bacterial nanocellulose (BC) integrated into neural interface applications as shown in Figure 9, highlighting its adaptability, biocompatibility, and performance. Figure 9a presents immunohistochemistry staining of neurons (NeuN, red), astrocytes (GFAP, green), and nuclei (DAPI, blue) at 2- and 4-weeks postimplantation, showing Au-BC electrodes elicited reduced gliosis and milder inflammation compared to Au–PI.^179^ Figure 9b shows immunofluorescence images of cortical cells cultured for 8 days on 3D-BC/graphene scaffolds versus 3D-graphene. Denser neural networks and enhanced neuron–astrocyte interactions were observed on 3D-BC/G, confirming

**Figure 9:**
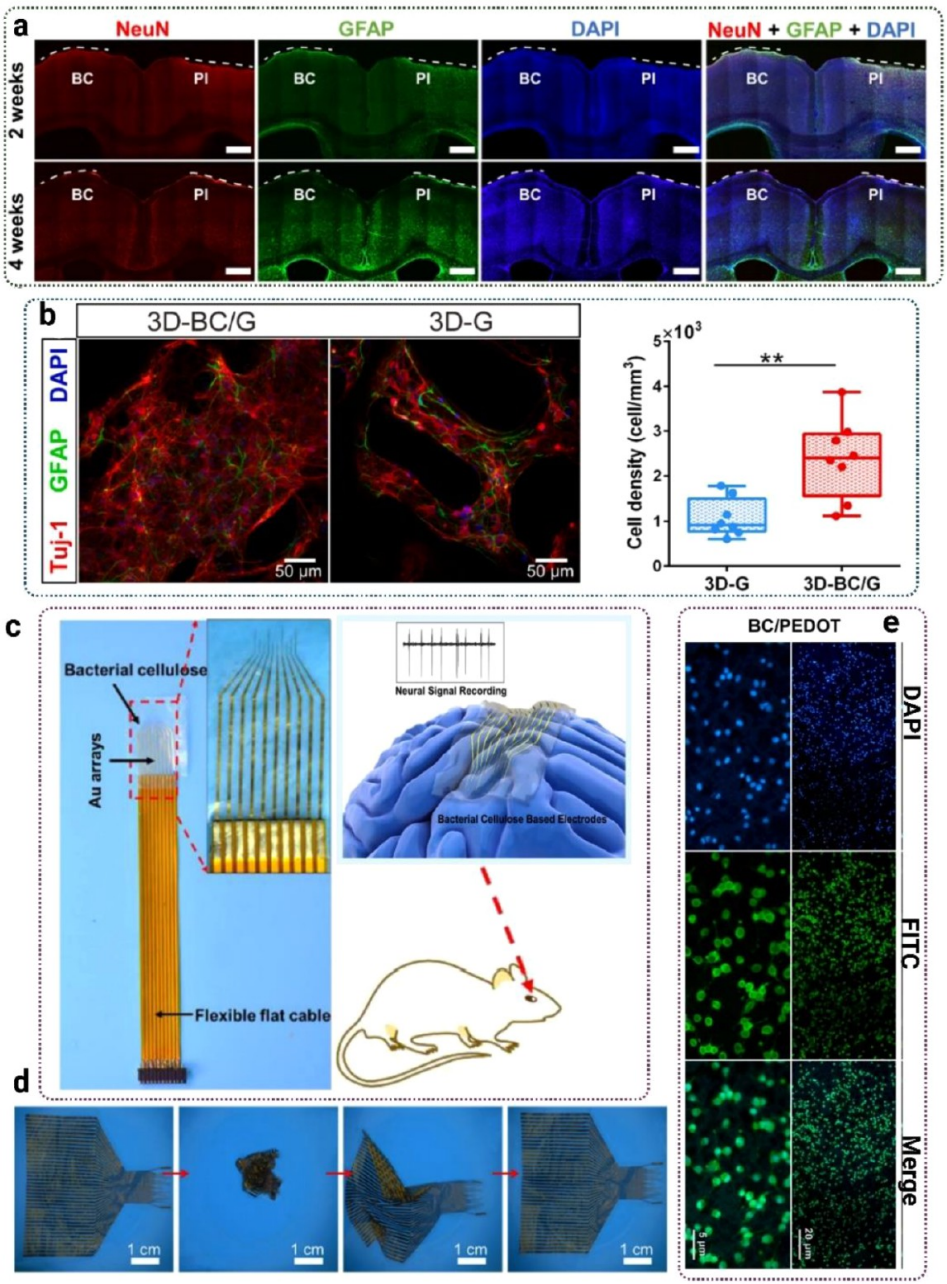
Immunohistochemistry staining of mice brain slices for long-term implantation of the Au-BC electrodes and Au–PI electrodes. (a) Immunohistochemistry staining of neurons (NeuN, red), nuclei (DAPI, blue), and astrocytes (GFAP, green) at 2- and 4-weeks post implantation of the Au-BC (left) and Au–PI (right) electrodes. The white dashed lines indicate the section (position) of the electrodes (a). Reproduced with permission from ref.^179^. Copyright 2018 American Chemical Society. Representative immunofluorescence images of cortical cells after 8 days of culture on 3D-BC/G (left) and 3D-G (right) stained for neurons (with Tuj-1, red), astrocytes (with GFAP, green), and nuclei (with DAPI, blue) (b). Reproduced with permission from ref.^186^. Copyright 2021 Elsevier. In-vivo evaluation of Au-BC electrodes in the recording system consists of flexible flat cable and Au-BCelectrodes. The six recording sites on the cerebral cortex of a rat (c). Flexibility evaluation of Au-BC electrodes. The highly flexible Au-BC electrodes remain intact after repeated twisting and untwisting for 100 times (d). Reproduced with permission from ref.^179^. Copyright 2018 American Chemical Society. Fluorescence images of BC/PEDOT nanofibers. Green fluorescence: Alexa Fluor 488 conjugated phalloidin for actin cytoskeleton; blue fluorescence: DAPI DNA stain for cell nuclei (e). Reproduced with permission from ref.^182^. Copyright 2015 American Chemical Society.

superior support for neural growth.^186^ Electrophysiological performance was demonstrated in Figure 9c, where Au-BC electrode arrays reliably recorded electrocorticography (ECoG) from the rat cortex with strong mechanical bonding and low contact resistance to flexible flat cables.^179^ Their durability was validated in Figure 9d, as electrodes remained intact after 100 cycles of twisting and untwisting, confirming excellent flexibility.^179^ Microscale patterning down to 5 μm and nanoscale roughness further improved tissue contact. Figure 9e highlights fluorescence images of PC12 cells on 3D BC/PEDOT nanofibers, showing healthy morphology, abundant pseudopodia, and robust actin cytoskeleton organization.^182^ These findings demonstrate that functionalized BC integrates conductivity, flexibility, and bioactivity, enabling stable long-term neural recording while promoting neural adhesion and regeneration.

The use of bacterial nanocellulose in neural interfaces represents a breakthrough in the field of smart bioelectronics. Its unique properties, such as biocompatibility, flexibility, and the ability to function with conductive and bioactive materials, make it an ideal candidate for a wide range of applications, from neuroprosthetics to drug delivery systems.

### 4.3 Tissue Engineering and Potential Integration in Bioelectronics

Bacterial nanocellulose (BNC) is increasingly recognized as a scaffold for tissue engineering because of its strength, flexibility, high water retention, and biocompatibility. These properties also position it as an enabling material for bioelectronics, where soft, biomimetic scaffolds must not only support tissue repair but also integrate with sensors, stimulators, and monitoring platforms. Functionalization strategies have been central to bridging BNC’s tissue engineering role with its bioelectronic potential. For example, Anton-Sales et al. developed single-layer and laminated BNC patches for soft tissue reinforcement, showing in vivo that multilayered patches provided the necessary tensile resistance for hernia repair with low adhesion rates.^189^ While the initial focus was surgical reinforcement, such mechanically stable scaffolds create a foundation for electrostimulation patches or bioelectronic meshes capable of delivering signals during healing.

Surface modification has also proven essential. Osorio et al. functionalized three-dimensional BNC with fibroblast-derived proteins such as collagen and fibronectin to mimic the extracellular matrix.^190^ This approach enhanced cell adhesion, proliferation, and integrin-mediated interactions while improving surface wettability and thermal stability. For bioelectronics, such biomimetic scaffolds are critical in ensuring stable tissue–device interfaces that can support embedded electrodes or conductive coatings without rejection. Zhou et al. advanced this further by engineering ultrafine BNC nanofibril-reinforced hydrogels with gelatin and glycerol, achieving high transparency, stretchability, and strong tissue adhesion.^191^ Importantly, these hydrogels could adhere to skin, monitor electrophysiological signals continuously for over 30 days, and act as smart wound dressings with NIR-triggered antibacterial functions. This demonstrates how BNC scaffolds can evolve into self-contained bioelectronic devices, merging tissue repair with real-time sensing and therapy.

Other functionalization methods enhance both sensing and therapeutic capacity. Farooq et al. incorporated carboxylated multiwalled carbon nanotubes (c-MWCNTs) and polyethylenimine into BC, enabling stable bacteriophage immobilization for ultrasensitive biosensing (detecting as low as 3 CFU/mL of *S. aureus*).^165^ Beyond food safety, this conductive, electroactive composite suggests applications in implant infection monitoring and electroactive scaffolds for neural or cardiac tissues where electrical cues drive regeneration. Similarly, Panaitescu et al. developed PHO/BC nanocomposites through melt processing, showing that 3 wt % BC increased PHO’s thermal stability by 25 °C, raised Young’s modulus by 76%, and improved tensile strength by 44% without cytotoxicity or inflammation.^192^ These biocompatible composites are highly relevant for vascular scaffolds and artificial arteries, which could be paired with bioelectronic monitoring systems to assess blood flow, mechanical strain, or graft integrity.

However, bacterial cellulose nanofibers were described as >5 μm in length and up to 100 nm in width, and PHO/BC composites were fabricated as dense films of 0.8–1 mm thickness. While the study confirmed good cytocompatibility and absence of inflammatory response, it did not report pore size distribution.

In cartilage regeneration, An et al. combined BNC with a decellularized cartilage extracellular matrix (DCEC) to form multilayered scaffolds that improved chondrocyte adhesion and migration.^193^ Although designed for tissue repair, the layered, bioactive structure could be adapted to electroactive scaffolds for musculoskeletal or neural prosthetics, particularly if paired with conductive fillers or stimulation electrodes.

Mira-Cuenca et al. demonstrated a different integration route by patterning BC films with superparamagnetic iron oxide nanoparticles (SPIONs) using screen printing.^194^ These patterned films were MRI-visible, enabling noninvasive implant monitoring in ex vivo models and demonstrated that BC films with 25 wt % SPIONs yield strong T_2_/T_2_* MRI contrast, but with saturated signals. According to ASTM F2119 guidance, future optimization requires reducing SPION loading and quantifying artifact-to-implant size ratios to balance visibility with minimized distortion.^195^ The ability to spatially control nanoparticle distribution not only allows tracking but also provides a blueprint for multifunctional smart implants, where biosensing and therapeutic features can be colocalized in predefined regions.

Figure 10 illustrates these diverse strategies—protein modification, conductive nanocomposites, hydrogels, and patterned nanoparticle films—and their outcomes. A representative example is the BGY-ICG-R hydrogel, designed as a smart skin for wound monitoring and therapy. Its schematic is shown in Figure 10a, while colorimetric changes with corresponding RGB signals under different pH conditions and when applied to wounds, the hydrogel enabled near-infrared (NIR) triggered antibacterial therapy, with images and pH analyses comparing treated versus untreated wounds.^191^ Another approach involves nanoparticle patterning, where BC films were functionalized with SPIONs or Au nanoparticles. Patterned configurations—SPIONs applied to one side, embedded between layers, or distributed on the surface—are displayed in Figure 10b, while MRI visualization of films inserted into tissue confirmed precise localization, Figure 10c.^192^ Importantly, wound closure rates significantly improved with NIR treatment compared to controls, as quantified in Figure 10d.^191^ Protein modification strategies further demonstrate regenerative potential. Immunocytochemistry revealed abundant collagen type I (red) and fibronectin (green) in modified BNC scaffolds, supporting extracellular matrix development, Figure 10e.^190^

**Figure 10:**
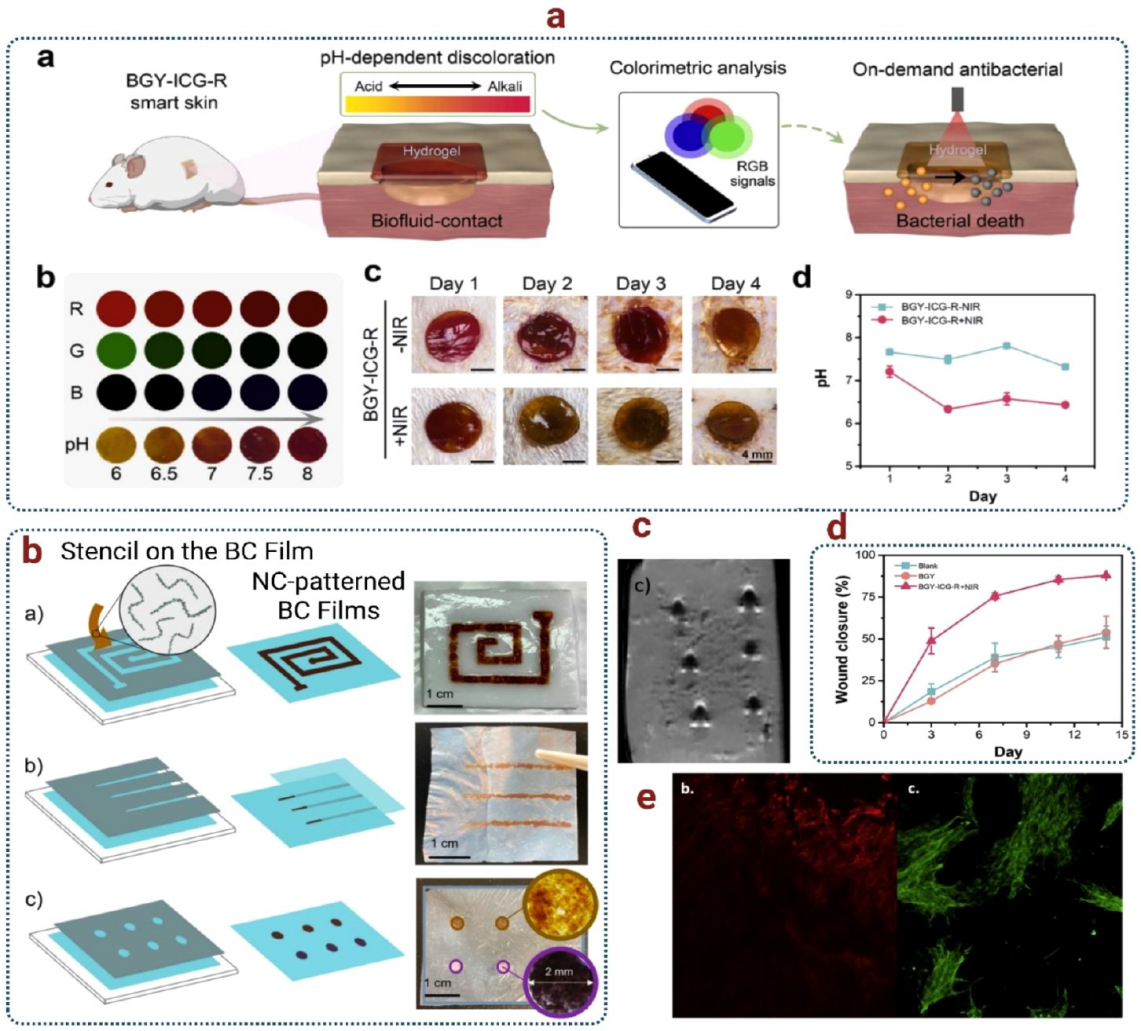
BGY-ICG-R hydrogel as a smart skin for colorimetric pH-monitoring and near-light-triggered antibacterial. (a) Schematic illustration of BGY-ICG-R as a smart skin. (b) The color of the BGY-ICG-R hydrogel and its corresponding RGB signals after the hydrogel exposing to the buffer solution with various pH values. (c) Images of the BGY-ICG-R hydrogel covering on the wound after treatment with or without NIR. (d) pH value analyses of the wound detected by BGY-ICG-R hydrogel after treatment with or without NIR (a). Reproduced with permission from ref.^191^. Copyright 2023 Elsevier. Patterning BC films with inorganic NPs. The BCf with NPs are added to the partially blotted BC films using a mask for depositing the fibers to specific areas: (a) BCf–SPIONs applied to one side of the film; (b) BCf–SPIONs placed between two BC films to isolate (lower row) the NPs. (c) Film functionalized with SPIONs (upper row) or Au NPs (lower row) on the surface (b). MR image of a film inserted in a meat loin were MRI, T2*-weighted coronal visualization of the film (c). Reproduced with permission from ref^192^. Copyright 2021 American Chemical Society. Wound closure rates of the different hydrogel treated (d). Reproduced with permission from ref.^191^. Copyright 2023 Elsevier. Immunocytochemistry demonstrating collagen type I (red) in Mod-BNC, c: immunocytochemistry demonstrating fibronectin (green) in Mod-BNC. Images in b and c represent an area of 375 × 375 μm in the xy-plane (e). Reproduced with permission from ref.^190^. Copyright 2019 Elsevier.

Table 4 presents selected studies showcasing how bacterial nanocellulose (BNC) has been engineered into devices ranging from biosensors with ultrasensitive detection of pathogens, toxins, and biomarkers, to neural interfaces that combine softness, conductivity, and biocompatibility for stable signal recording and stimulation, and tissue-engineering platforms that integrate sensing, antibacterial activity, and real-time monitoring through multifunctional hydrogels and MRI-visible films These examples highlight BNC’s transition from a passive scaffold to an active bioelectronic material, enabling applications in diagnostics, neural repair, and smart implantable systems.

**Table 4:** Performance Comparison Table for Device Type Produced from Bacterial Nanocellulose

**Research Area**	**Nomenclature**	**Performance Metrics**	**Functions/Applications**	**Reference**
Biosensor	BC/G-PEG/ACE2/BSA/Nafion	Limit of detection (LOD): 4.26 × 10^–18^ g mL^–1^ of spike protein (SP) and 0.05 viral RNA copies mL^–1^.	Rapid, ultrasensitive, and label-free detection of SARS-CoV-2 in clinical samples	^156^
		Detection time: 10 min.		
		Cost: US$3.50 per test		
		Stability: < 4% drift over 60 min in PBS.		
	Pd@PCN-222 CRISPR nanozyme	Limit of detection (LOD): 1.21 pg/mL in buffer solution and 28 pg/mL in real corn samples.	Rapid, ultrasensitive, and preamplification-free electrochemical detection of ochratoxin A (OTA), a common foodborne mycotoxin	^157^
		Linear detection range: 0.005–50 ng/mL ochratoxin A (OTA).		
		Stability: Retained 93.6% of initial signal after 2 weeks at 4 °C.		
	Ag NPs-BCM substrate	Limit of detection (LOD): As low as 2.9 × 10^–8^ M for GSH and detects 4-MBN down to 10^–8^ M.	Sensitive and reproducible detection of glutathione (GSH) in serum using SERS	^158^
		Stability: Retained performance for 45 days under light-protected storage, with only ∼ 5% intensity loss at 15 days.		
	BC-Zn-BIM@Lac electrode	Bandgap: Reduced from 1.275 eV (BC–Zn^2+^ – BIM) to 0.681 eV for BC-Zn-BIM → semiconductor-like properties.	Sensitive, selective electrochemical detection of Bisphenol A (BPA) in food packaging and environmental samples	^159^
		Electrochemical impedance (Ret): BC-Zn-BIM@Lac electrode: 180.98 Ω		
		Stability: Retained 94.1% of initial current after 2 weeks storage at 4 °C.		
	BNC immunosensor with IDE/CHT/CS/anti-p53	Detection limit (LOD): As low as 0.16 U cell mL^–1^ (best performance with 3-bilayer LbL films).	Electrochemical immunosensor for detecting the p53 cancer biomarker in cell lysates and Early cancer diagnostics, since abnormal p53 expression is linked to many tumors.	^163^
		Detection range: 0.01 to 1000 U cell mL^–1^ (MCF7 breast cancer lysates).		
		The carbon interdigitated electrodes screen-printed on BNC were 2.5 cm × 0.8 cm overall, with track widths of 1 mm, and relatively large finger gaps compared to conventional photolithographic IDEs		
	20BG–5–60 humidity sensor	Conductivity: Increased to 3.88 × 10^–11^ S/m after reduction (20BG–5–60).	Human health monitoring (respiration patterns). Noncontact sensing (finger tracking, spatial localization). Potential use in human–machine interfaces and artificial intelligence (AI) applications.	^164^
		Response/recovery times: 13 s (response) and 47 s (recovery).		
		Long-term stability: Stable performance over 60 days.		
	BC/c-MWCNTs-PEI-phage biosensor	Detection limit (LOD): 3 CFU·mL^–1^ in PBS and 5 CFU·mL^–1^ in milk.	Ultrasensitive, selective, and stable electrochemical biosensor for *Staphylococcus aureus* detection and first biosensor to use high-density oriented phage immobilization on a bacterial cellulose composite to ensure strong binding and lytic activity.	^165^
		Detection range: 10^2^ – 10^7^ CFU·mL^–1^ (PBS); 10^2^ – 10^6^ CFU·mL^–1^ (milk).		
		Stability: Maintained performance for ≥ 6 weeks at 4 °C (RSD ≤ 0.15).		
	BCM-Au NPs-Ap/TNF-α/Au@4-MBN@Ag-Ap SERS biosensor	Detection limit (LOD): 0.35 pg/mL TNF-α.	Ultrasensitive and selective detection of Tumor Necrosis Factor-alpha (TNF-α), a key inflammatory cytokine linked to cancer and autoimmune diseases.	^166^
		Detection range: 10^–8^ – 10^–4^ mg/mL.		
	NBC-NBN composite nanofluidic membrane device	Zeta potential/surface charge density: NBC: – 73.8 mV (−5.3 mC m^–2^). NBN: – 29.2 mV (−2.1 mC m^–2^).	Nanofluidic energy harvester capable of converting both salinity gradients (blue energy) and low-grade heat (thermo-osmotic energy) into electricity.	^167^
		Internal resistance (Rct): NBC50-NBN50:60 kΩ.		
		Stability: Maintained ∼ 90% power after 7 weeks in seawater/river water. Stable after boiling in ultrapure water for 4 h.		
	Ag NPs/Cu2O/CuO/BNC/CPE	Detection limit: 42.3 nM and two linear ranges 0.1–76 and 76–1000.0 μM	Sensitive and selective electrochemical detection of Metformin (MET) for pharmaceutical and clinical monitoring.	^168^
	HNC/AgNPs-PVA modified SPGE lactate sensor	Thermal stability: Stable up to 450 °C.	Electrochemical enzymatic biosensor for lactate detection in sweat. Wearable health monitoring, especially sports and exercise physiology. Early detection of muscle fatigue via sweat lactate levels.	^169^
		Hydrogen peroxide sensing: Linear range: 0–7 mM H_2_O_2_. Lactate sensing: Linear range: 0–25 mM lactate.		
		Limit of detection (LOD): 0.56 mM.		
	BC/PPy/TiO_2_–Ag biosensor	Detection limit (DL): 0.5 McFarland standard (∼1.5 × 10^8^ CFU/mL).	Rapid and low-cost detection and monitoring of bacterial growth via electrical resistance changes.	^170^
		Sensitivity and selectivity: Higher sensitivity to Gram-negative bacteria (*E. coli* > *A. hydrophila*) compared to Gram-positive. Within Gram-positive, better response to *S. aureus* vs *S. epidermidis*.		
	BC/PVAN/PANI nanocomposite membranes	Electrical conductivity: Up to (4.5 ± 1.7) × 10^–2^ S cm^–1^	Electrically conductive and biocompatible bioelectronic interface membrane.	^171^
		Thermal stability: Stable up to 225 °C		
		Cytocompatibility: Neural stem cells (SVZ) viability: ∼90% after 7 days (non-cytotoxic).		
	BC/CMC/AuNPs composite hydrogel	Thermal stability: Decomposition temperature increased to 730 °C	Selective preconcentration and ultrasensitive detection of glutathione (GSH), a critical thiol biomarker for Alzheimer’s disease diagnosis.	^175^
		GSH detection (via LDI-MS): Linear range: 50–10,000 nM.		
		Limit of detection (LOD): 49–54 nM (below Alzheimer’s diagnostic cutoff of 2700 nM).		
	PAM/TOBC/LiTFSI dual-network ionic thermoelectric hydrogel	Conductivity: up to 0.71 mS cm^–1^	Flexible, self-powered ionic thermoelectric hydrogel sensor for converting low-grade human body heat into electricity.	^176^
		Sensing performance: Gauge factor (GF): 1.34 (0–300% strain). 3.91 (350–850%). 13.15 (900–1400%).		
		Response/recovery times: 135/160 ms.		
		Stability: Retention rates after 100 bending cycles: 98.9% Seebeck, 98.6% conductivity. After 100 stretching cycles: 96.8% Seebeck, 96.5% conductivity		
Neural Interfaces	Au-BC electrodes	Impedance at 1 kHz: 55.6 ± 5.0 kΩ (Au-BC) vs 70.1 ± 5.8 kΩ (Au–PI).	Soft, biocompatible neural interfacing electrodes for electrocorticography (ECoG). Recording brain activity (e.g., epileptic seizures, neural mapping).	^178^
		Young’s modulus: 120 kPa		
		Thermal stability: Stable up to 300 °C		
		Biocompatibility: PC-12 neural cell viability: 97 ± 1% live cells after 3 days.		
		Hemolysis assay: Negligible red blood cell lysis.		
		In vivo implantation: Lower astrocyte inflammatory response compared to Au–PI electrodes (GFAP fluorescence intensity: 0.31 ± 0.01 vs 1.1 ± 0.03, *p* < 0.001). Long-term subcutaneous implants: Minimal immune reaction after 8 weeks. In vivo neural recording: Successfully recorded epileptiform activity (5–25 Hz) from rat cerebral cortex. High-throughput mapping possible with 10-channel arrays.		
	3D BC/PEDOT composite nanofibers	Tensile strength: 4.0 ± 0.6 MPa. Young’s modulus: 0.83 ± 0.2 MPa. Stable CV signals under bending (up to 180°) and after 1000 cycles.	Neural stimulation and recording (prostheses, brain–machine interfaces). Implantable biosensors with stable signal transduction. Smart drug delivery systems controlled by electrical stimulation.	^182^
		PC12 neural cell viability: ∼95% (low cytotoxicity).		
		Electrical stimulation increased calcium influx in PC12 neural cells.		
	BC/PEDOT/GO composite nanofibers	Impedance at 1 kHz: 15 kΩ (vs. 40 kΩ for BC/PEDOT).	Neural tissue engineering – supports neurite outgrowth and orientation. Electrode–cell interfaces for stimulation and recording. Biomedical and regenerative medicine scaffolds mimicking ECM	^183^
		Water contact angle: 53.2		
		PC12 neural cell viability: ∼95% on BC/PEDOT/GO		
		Young’s modulus: 1.445 ± 0.112 GPa		
	(BC/oxBC)@PANI fibers	Elastic modulus: ∼ 102 kPa (close to soft tissue).	Continuous monitoring of subtle organ motions (heart, vessels, muscles). Wearable and implantable health monitoring.	^184^
		Stretchability: withstands tensile strain up to 2.5% with sensitivity to tiny forces (8.8 × 10^–3^ N)		
		Low cytotoxicity in cell assays → suitable for implantation.		
		Real-time detection of bullfrog heartbeat.		
	Recombinant protein–modified bacterial cellulose scaffolds	Biocompatibility (viability): NPCs: > 95% viability after 7 days (Live/Dead staining).	Supports neural progenitor adhesion, neurite outgrowth, and network formation. Enhances MSC adhesion and proliferation for regenerative scaffolds. Adaptable platform where protein cues can be customized for specific cell types.	^185^
		MSCs: > 93% viability after 7 days.		
	3D-BC/G scaffold	Neural stem cells (NSCs) cultured on 3D-BC/G showed >99% viability after 3 days	Promoting NSC proliferation, neuronal differentiation, and network formation. Providing a physiologically relevant 3D in vitro model for neurodegenerative disease studies. Potential use in neural repair (spinal cord injury, Parkinson’s, Alzheimer’s).	^186^
		RNA-Seq identified 31 differentially expressed cell-cycle genes and upregulation of FoxO, Wnt, Notch, and TGF-β pathways regulating proliferation and neurogenesis		
Tissue Engineering	BGY-ICG-R hydrogel	Electrophysiological Performance: Ionic conductivity: 1.85 S/m. Stable ECG and EMG signal recording after 30 days exposure. Detected throat vibration (voice) and facial expression signals reliably.	Adhesive, transparent, conductive bioelectrode for epidermal signal monitoring (ECG, EMG, motion, voice). Flexible and stable E-skin platform for humidity, pressure, and handwriting recognition.	^191^
		Electronic Skin (E-skin): Humidity sensing: reproducible detection at 50–80% RH (deep breathing, airflow). Pressure sensing: discriminated loads of 10, 20, 50 g.		
		Signature sensing: produced distinct current responses for letters/numbers after 30 days.		
		Antibacterial: ∼ 100% kill ratio for *S. aureus* and *E. coli* after 10 min NIR (1 W/cm^2^).Wound healing in vivo: closure rate 88% at 14 days (vs ∼ 51–54% controls).		
	BC/SPIONs patterned films	MRI visibility: SPION patterns produced strong hypointense T2 and T2 contrast* on 7 T MRI. Clear detection of 5 × 5 mm^2^ and 1 mm dot arrays, even inside ex vivo pork muscle tissue.	BC/SPIONs films allow noninvasive monitoring of implant position, deformation, shrinkage, or adhesions by MRI, reducing the need for exploratory surgery	^194^
		Stability: No leaching of SPIONs observed after immersion in water for 7 days or PBS for 7 days (UV–Vis confirmed).		
		Leaching observed only after 4 weeks under strong mechanical shaking in water. Protection via BC overlayer is possible		

## 5 Challenges and Perspectives on Using Bacterial Nanocellulose in Bioelectronics

The recent advances and case studies in bacterial nanocellulose bioelectronics demonstrate the material’s versatility and potential across various applications.^195–197^ From conductive composites and biosensors to tissue engineering scaffolds and environmental sensors, BNC is proving to be a valuable material in developing innovative bioelectronic devices. The successful implementation of BNC in wearable health monitoring devices, implantable sensors, wound dressings, and flexible energy storage devices underscores its potential to revolutionize the field of bioelectronics. Ongoing research and development efforts are essential to further enhance the properties and functionality of BNC-based materials, addressing existing challenges and paving the way for their widespread adoption in smart bioelectronics.

### 5.1 Challenges of Using Nanocellulose in Bioelectronics

Despite its advantages, the production of BNC at an industrial scale remains a challenge. The fermentation process is time-consuming and requires specific conditions to optimize yield and quality. Recent genetic engineering has demonstrated clear improvements in bacterial nanocellulose production. For example, in *Komagataeibacter hansenii*, manipulation of the *cmcax* gene was shown to alter both yield and fibril structure: overexpression of *cmcax* enhanced cellulose output, while disruption reduced production and shifted fibrils toward cellulose II.^198^ Similarly, in *K. xylinus* BPR2001, knockout of *gdh* (glucose dehydrogenase) eliminated gluconic acid byproducts and resulted in up to a 230% increase in cellulose yield under shaken conditions compared with the wild type. Overexpression of the entire *bcsABCD* operon has also produced two- to 4-fold higher cellulose titers relative to native strains. These exemplars demonstrate how targeted genetic modifications can substantially boost BNC yields, supporting the potential of engineered strains for scalable production.

Another scaling up production while maintaining the unique properties of BNC poses significant technical and economic challenges, for instance, Jozala et al., review reports g/L yields across lab- and pilot-scale reactors (1–60 g/L) and a theoretical 80-ton yield from a 500 m^3^ system, but it does not present cost-per-kg analyses or industrial tonnage benchmarks and current reactors include static trays, stirred tanks, and modified airlift, are positioned at lab-to-pilot scale with costs > $200–500/kg, whereas industrial viability requires >1000 tons/year at < $50/kg.^199^

Meanwhile, recent studies emphasize cost reduction using agro-industrial wastes (e.g., potato juice, orange peel, cheese whey) as nutrient sources and advanced reactor designs (fed-batch, airlift, rotating biological contactors) to enhance oxygen transfer and yields. Genetic engineering further improves strain performance on low-cost substrates. Life cycle assessments report carbon footprints between 34–296 kg CO_2_-eq/kg, with waste-derived feedstocks reducing impacts by up to 76%. However, despite these advances, current BNC reactors remain at lab-to-pilot scale (g/L yields, > $200–500/kg), far from the industrial benchmark of tonnage-scale production at < $50/kg. Bridging this gap will require both scale-up and technoeconomic optimization.^200^

Speaking of the techno-economic feasibility of BNC production, it has been quantitatively assessed in a limited number of studies. Dourado et al. conducted one of the earliest process simulations for industrial-scale BNC fermentation under static culture, revealing a cost structure strongly dominated by capital expenses (CAPEX).^201^ In their model, direct capital costs amounted to approximately US$ 1.3 million, of which nearly 70% were associated with installation, piping, and construction, while equipment itself accounted for the remaining 30%. Operating expenditures (OPEX) were likewise skewed toward fixed costs: equipment depreciation and maintenance represented 51% of total costs, followed by personnel at 32%, whereas raw materials and energy contributed only 7%, highlighting a CAPEX-driven process with modest consumable costs. More recent studies modeling kombucha-derived BC provide complementary insights into the balance of capital and operational drivers. Behera et al. simulated a 60-ton/year facility using SuperPro Designer, estimating a total investment (CAPEX) of US$ 13.72 million, with equipment purchases alone costing US$ 2.1 million.^202^ Notably, 69% of this investment was tied to downstream equipment (belt filters, dryers, extruders) and 55% to static fermentation units, underscoring the equipment-intensive nature of BNC processes. Annual OPEX was projected at US$ 3.8 million, of which 89% derived from facility-dependent costs and labor, while raw material costs (sucrose, NaOH, tea leaves) were relatively minor inputs. This configuration yielded a payback time of 4.23 years, a return on investment of 23.6%, and an internal rate of return of 16.5%, with a minimum selling price of US$ 1.63/kg of cellulose.

Complementary to these simulations, recent reviews emphasize that efforts to reduce OPEX often focus on coculture strategies and the use of low-cost substrates such as brewery waste or agricultural residues.^203^ While such approaches can significantly reduce raw material costs, they frequently introduce greater engineering complexity and higher CAPEX, especially when employing advanced reactor designs such as dual-vessel or mesh dispenser systems.

In addition, recent life cycle assessment (LCA) studies have quantified the cradle-to-gate greenhouse gas (GHG) emissions of nanocellulose production, including bacterial cellulose (BC). A broad meta-analysis of 95 studies reported values ranging from 34 to 296 kg CO_2_-eq per kg of nanocellulose depending on feedstock and process configuration.^204^ Within this range, bacterial cellulose from static fermentation has been estimated at 200–250 kg CO_2_-eq per kg of product, with the high burden driven primarily by glucose feedstock requirements and energy-intensive aeration and purification. Importantly, valorization of agro-industrial waste streams as carbon sources can reduce these impacts by more than 50%, lowering BC’s footprint closer to that of mechanically fibrillated nanocellulose (40–80 kg CO_2_-eq/kg).^205^ And while bacterial cellulose is widely regarded as biocompatible, several ISO 10993 end points remain insufficiently tested, including chronic toxicity, genotoxicity, sensitization, and long-term implantation. To meet regulatory expectations, future studies should address these under GLP-compliant chronic testing conditions, particularly for applications involving permanent or long-term implants.

Thus, integrating BNC with conventional electronic components requires overcoming several hurdles. For instance, BNC’s natural insulating properties necessitate modifications to enhance conductivity, which can complicate the manufacturing process. No long-term conductivity decay rates have been published thus this remains a critical gap. Achieving stable and reliable interfaces between BNC and electronic elements is critical for device performance and durability.^206^ The long-term stability of BNC-based bioelectronic devices is a concern, particularly in harsh environmental conditions or when exposed to bodily fluids. Ensuring that BNC retains its properties and functionality over extended periods is crucial for medical implants and long-term wearables.^207^ While BNC can be functionalized, the range and efficiency of these modifications can be limited by the material’s inherent properties. Achieving uniform dispersion of nanoparticles or ensuring consistent chemical modifications across large batches of BNC can be challenging.^94^ Uniformity of nanoparticle loading could be standardized by reporting the coefficient of variation across ≥ 100 cm^2^ sheets, as suggested for reproducibility. As with any new material intended for medical use, BNC must undergo rigorous testing to meet regulatory standards. Ensuring the safety and efficacy of BNC-based devices requires extensive research and clinical trials, which can be time-consuming and costly.^3^

The use of bacterial nanocellulose in bioelectronics offers numerous advantages, including biocompatibility, mechanical strength, customizability, hydrophilicity, and sustainability. These properties make BNC an attractive material for a wide range of applications, from medical implants to wearable sensors. For instance, bacterial nanocellulose (BNC) is highly stable in vivo due to the absence of endogenous cellulases in human tissues, yet this stability can be tuned through oxidative and enzymatic strategies. Melro et al. report that degradation of BNC under physiological conditions occurs primarily via hydrolysis, oxidative pathways, and exogenous cellulase activity, with in vivo evaluations showing partial degradation of wound dressings and drug carriers and implants persisting for up to one year.^208^ Complementing this, Kaczmarek and Białkowska describe enzymatic functionalization methods such as cellulase-mediated hydrolysis, laccase/TEMPO oxidation, and lytic polysaccharide monooxygenase activity, which introduce reactive groups and increase susceptibility to biodegradation, as well as indirect strategies involving immobilized oxidative enzymes for controlled disassembly of the nanocellulose network.^209^ These studies demonstrate that oxidative and enzymatic tailoring offers a viable route to achieve tunable degradation and adjustable in vivo half-life of BNC scaffolds, enabling design flexibility for applications ranging from long-term implants to resorbable biomedical devices. However, challenges such as scalability, integration with electronic components, long-term stability, functionalization limitations, and regulatory hurdles must be addressed to fully realize the potential of BNC in bioelectronics. Ongoing research and technological advancements are essential to overcome these challenges and pave the way for the widespread adoption of BNC-based smart bioelectronic devices.

### 5.2 Future Perspectives

BNC research is rapidly advancing, driven by its unique properties such as high crystallinity, purity, biodegradability, biocompatibility, and mechanical strength.^5,25,210^ These features make BNC an excellent candidate for functionalization, expanding its potential applications across various fields.^159,211^ A notable trend in BNC research is the creation of multifunctional nanocomposites. By incorporating materials such as metallic NPs, polymers, CNTs, biomolecules and graphene into the BNC matrix, researchers are developing nanocomposites with improved electrical, thermal, medical and optical properties.^5,140,152,161^ These advanced materials are being tested for use in flexible electronics, biosensors, conductive materials and healthcare, significantly broadening the scope of BNC applications. Bioengineering of BNC-producing bacteria is another important development. By modifying metabolic pathways and introducing specific genes to produce BNC, biomanufacturing BNC with tailored properties can be achieved.^212–214^

This approach not only optimizes production and yield but also allows for the creation of BNC with specific characteristics, such as increased porosity or functional groups. These modifications are particularly useful for applications in biomedicine and environmental science, where precise control over material properties is essential. Furthermore, functionalizing BNC with biomolecules such as proteins, enzymes, and peptides as well as synthetic nanomaterials such as graphene and gold is gaining popularity. This strategy imparts specific biological activities to BNC, making it suitable for applications in tissue engineering, wound healing, and drug delivery.^215,216^ For example, protein-functionalized BNC and graphene-functionalized BNC can be used in wound dressings to enhance healing by targeting specific proteins involved in the wound healing process.^216,217^ Also, peptide-functionalized BNC can enhance cell adhesion and growth in tissue scaffolds.^218^ These innovative techniques are expanding the uses of BNC in various sectors, showcasing its versatility and potential.

The potential for commercializing BNC-based product is vast and promising.^200^ As reported by the global nanocellulose market, the global nanocellulose market was valued at USD 419 million in 2023 and is projected to reach USD 1.24 billion by 2029. The market is segmented into cellulose nanocrystals (CNCs), cellulose nanofibrils (CNFs), microfibrillated cellulose (MFCs), and BNC. MFCs hold the largest market share, followed by CNFs, with CNCs and BNC accounting for smaller shares.^219^ Regarding the BNC application in the medical field, it is being developed for advanced wound dressings, drug delivery and screening, artificial blood vessels, tissue and scaffolds engineering, or diagnostics and biosensors.^36^ The ultrafine structure, mechanical robustness, biodegradability, nontoxic nature, high water retention capacity and reactivity for chemical modification of BNC make it highly advantageous for biomedical applications.^36^ Several BNC-based wound dressings and implants are already on the market, with more in development.^220^ Functionalized BNC systems for drug delivery are also being designed to deliver therapeutic agents in a controlled and targeted manner. By incorporating drug molecules into the BNC matrix or functionalizing the BNC surface with targeting ligands, these systems can improve the effectiveness and safety of treatments for various diseases. The scalability of BNC production, combined with its customizable nature, makes it an attractive option for pharmaceutical companies seeking to innovate in drug delivery technologies.

Environmental applications of functionalized BNC are also emerging, particularly in water purification and air filtration. BNC’s high surface area and ability to function with various active agents make it excellent material for adsorbing contaminants from water and air.^221,222^ Researchers are exploring the use of BNC-based filters and membranes to remove heavy metals, organic pollutants, pesticides and pathogens, aiming to provide sustainable and efficient solutions for environmental remediation.^222,223^ In the textile industry, BNC is being investigated as a sustainable alternative to synthetic fibers. Functionalized BNC can be processed into fibers and fabrics with desirable properties such as high tensile strength, biodegradability, and the ability to be modified for specific functions, such as antimicrobial activity.^224^ This potential could revolutionize the textile industry by offering eco-friendly materials that meet the performance standards of conventional textiles.

Future research and development in functionalized BNC should focus on several key areas to fully realize its potential. Developing cost-effective and scalable production methods is crucial to making BNC a commercially viable material.^200^ Exploring new functionalization methods to further enhance BNC’s properties is also important. Advanced chemical, physical, and biological techniques can introduce a wider range of functionalities, enabling the creation of BNC materials tailored for specific high-performance applications. Additionally, developing standardized protocols for functionalization and characterization will ensure consistency and reproducibility across different research and industrial applications.

Interdisciplinary collaboration in various topics will be essential in advancing BNC research as recently observed, when combining expertise from materials science, biotechnology, and engineering which will facilitate the development of innovative BNC-based solutions for complex challenges. Figure 11a shows the consolidated numbers of published research from 2005 to 2025 and topics, Figure 11b, when searching bacterial cellulose and bacterial nanocellulose. It should be noted that the terms *bacterial nanocellulose (BC)* and *bacterial cellulose (BNC* are used interchangeably in the literature. Figure 11 therefore highlights that both terms must be considered in literature searches to capture the full breadth of interdisciplinary research and the state-of-the-art development of this material.^225^ Collaborative efforts can also drive the creation of multifunctional BNC materials that leverage the strengths of different scientific fields. Addressing regulatory considerations will be crucial for the widespread adoption of functionalized BNC. Establishing clear guidelines for the safe production, use, and disposal of BNC byproducts will help mitigate potential risks to human health and the environment. The future of functionalized BNC research is promising. Emerging trends, commercialization potential, and promising research directions are leading to innovative applications of BNC in various industries. The continued advancement of this field will undoubtedly lead to new materials and technologies that significantly impact society.

**Figure 11:**
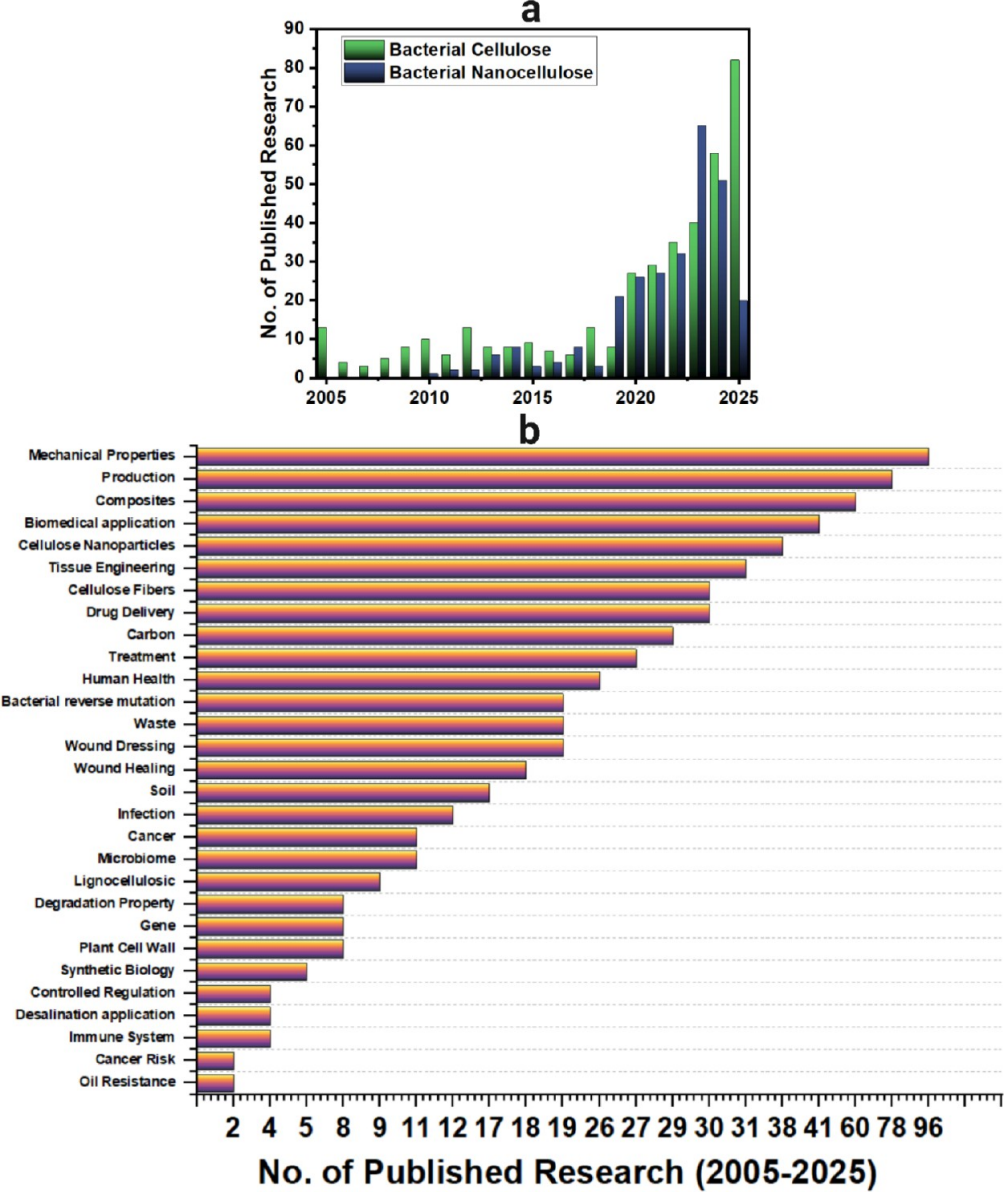
Consolidated number of published research for the past 20 years (a) and topics (b) upon searching bacterial nanocellulose and bacterial cellulose. *Adapted from* Science.gov.^225^

## 6 Conclusion

Bacterial nanocellulose (BNC) has emerged as a highly versatile and valuable material in the development of smart bioelectronics. Its biocompatibility, mechanical strength, and adaptability through functionalization make it ideal for a variety of applications, from biosensors to tissue engineering. Recent advancements in BNC functionalization, such as incorporating conductive materials and nanoparticles, have expanded its potential in smart bioelectronics specifically for biosensors, neural interfaces, and tissue engineering scaffolds. These developments demonstrate the material’s ability to seamlessly integrate with biological systems, providing real-time monitoring, enhancing tissue regeneration, and enabling precise drug release. BNC’s sustainable production and tunable properties make it a promising material for future bioelectronic applications. As ongoing research continues to refine functionalization techniques and address challenges like large-scale production, BNC is expected to play a pivotal role in the next generation of biomedical devices and environmental technologies, significantly improving both healthcare outcomes and sustainability.

## References

[1] Gordon E. B., Choi I., Amanipour A., Hu Y., Nikkhah A., Koysuren B., Jones C., Nitin N., Ovissipour R., Buehler M. J., Blackstone N. T., Kaplan D. L. (2025). Biomaterials in
cellular agriculture and plant-based foods for the future. Nat. Rev. Mater.

[2] Brown A. J. (1886). XLIII.—On
an acetic ferment which forms cellulose. J.
Chem. Soc., Trans.

[3] Sharma C., Bhardwaj N. K. (2019). Bacterial nanocellulose: Present status, biomedical
applications and future perspectives. Mater.
Sci. Eng.

[4] Zhong C. (2020). Industrial-Scale
Production and Applications of Bacterial Cellulose. Front. Bioeng. Biotechnol.

[5] Ullah M. W., Alabbosh K. F., Fatima A., Islam S. U., Manan S., Ul-Islam M., Yang G. (2024). Advanced biotechnological
applications
of bacterial nanocellulose-based biopolymer nanohybrids: A review. Adv. Ind. Eng. Polym. Res.

[6] Walling B., Bharali P., Ramachandran D., Viswanathan K., Hazarika S., Dutta N., Mudoi P., Manivannan J., Manjunath Kamath S., Kumari S. (2023). In-situ
biofabrication
of bacterial nanocellulose (BNC)/graphene oxide (GO) nano-biocomposite
and study of its cationic dyes adsorption properties. Int. J. Biol. Macromol.

[7] Wang J., Tavakoli J., Tang Y. (2019). Bacterial
cellulose production, properties
and applications with different culture methods – A review. Carbohydr. Polym..

[8] Gomes B. S., Simões B., Mendes P. (2018). The increasing dynamic,
functional
complexity of bio-interface materials. Nat.
Rev. Chem.

[9] Wang F., Kim H. J., Park S., Kee C. D., Kim S. J., Oh I. K. (2016). Bendable and flexible
supercapacitor based on polypyrrole-coated
bacterial cellulose core-shell composite network. Compos. Sci. Technol.

[10] Peng S., Xu Q., Fan L., Wei C., Bao H., Xu W., Xu J. (2016). Flexible polypyrrole/cobalt
sulfide/bacterial cellulose composite
membranes for supercapacitor application. Synth.
Met.

[11] Peng S., Fan L., Wei C., Liu X., Zhang H., Xu W., Xu J. (2017). Flexible polypyrrole/copper sulfide/bacterial cellulose nanofibrous
composite membranes as supercapacitor electrodes. Carbohydr. Polym.

[12] Sheng N., Chen S., Yao J., Guan F., Zhang M., Wang B., Wu Z., Ji P., Wang H. (2019). Polypyrrole@TEMPO-oxidized
bacterial cellulose/reduced graphene oxide macrofibers for flexible
all-solid-state supercapacitors. Chem. Eng.
J.

[13] Fortunato, E. ; Gaspar, D. ; Duarte, P. ; Pereira, L. ; Águas, H. ; Vicente, A. Optoelectronic Devices from Bacterial NanoCellulose. In Bacterial Nanocellulose: from Biotechnology to Bio-Economy. Gama, M. ; Bielecki, S. ; Dourado, F. Eds.; Elsevier, 2016 pp. 179–197.

[14] Zhao C., Park J., Root S. E., Bao Z. (2024). Skin-inspired soft
bioelectronic materials, devices and systems. Nat. Rev. Bioeng.

[15] Leitao A. F., Gupta S., Silva J. P., Reviakine I., Gama M. (2013). Hemocompatibility study of a bacterial cellulose/polyvinyl alcohol
nanocomposite. Colloids Surf., B.

[16] Liu D., Meng Q., Hu J. (2023). Bacterial
Nanocellulose Hydrogel:
A Promising Alternative Material for the Fabrication of Engineered
Vascular Grafts. Polymers.

[17] Jacob S., Reshmy R., Antony S., Madhavan A., Sindhu R., Kumar Awasthi M., Kuddus M., Pillai S., Varjani S., Pandey A. (2022). Nanocellulose in tissue engineering and bioremediation:
mechanism of action. Bioengineered.

[18] Fu L., Zhang J., Yang G. (2013). Present status
and applications of
bacterial cellulose-based materials for skin tissue repair. Carbohydr. Polym.

[19] Babaei-Ghazvini A., Patel R., Vafakish B., Yazdi A. F., Acharya B. (2024). Nanocellulose
in targeted drug delivery: A review of modifications and synergistic
applications. Int. J. Biol. Macromol.

[20] Munawaroh H. S. H., Anwar B., Yuliani G., Murni I. C., Arindita N. P. Y., Maulidah G. S., Martha L., Hidayati N. A., Chew K. W., Show P.-L. (2023). Bacterial cellulose
nanocrystal as drug delivery system
for overcoming the biological barrier of cyano-phycocyanin: a biomedical
application of microbial product. Bioengineered.

[21] Hasan N., Rahman L., Kim S.-H., Cao J., Arjuna A., Lallo S., Jhun B. H. W., Yoo J.-W. (2020). Recent
advances
of nanocellulose in drug delivery systems. J.
Pharm. Investig.

[22] Das S., Ghosh B., Sarkar K. (2022). Nanocellulose
as sustainable biomaterials
for drug delivery. Sens. Int..

[23] Müller A., Zink M., Hessler N., Wesarg F., Müller F. A., Kralisch D., Fischer D. (2014). Bacterial
nanocellulose with a shape-memory
effect as potential drug delivery system. RSC
Adv.

[24] Suleimenova A., Frasco M., Soares Da Silva F. A.
G., Gama M., Fortunato E., Sales M. G. F. (2023). Bacterial nanocellulose membrane
as novel substrate for biomimetic structural color materials: Application
to lysozyme sensing. Biosens. Bioelectron.:
X.

[25] De
Assis S. C., Morgado D. L., Scheidt D. T., De Souza S. S., Cavallari M. R., Ando Junior O. H., Carrilho E. (2023). Review of Bacterial
Nanocellulose-Based Electrochemical Biosensors: Functionalization,
Challenges, and Future Perspectives. Biosensors.

[26] Akki A. J., Jain P., Kulkarni R., Badkillaya R. R., Kulkarni R., Zameer F., Anjanapura V. R., Aminabhavi T. M. (2024). Microbial biotechnology alchemy: Transforming bacterial
cellulose into sensing disease- A review. Sens.
Int..

[27] Golmohammadi H., Morales-Narváez E., Naghdi T., Merkoçi A. (2017). Nanocellulose
in Sensing and Biosensing. Chem. Mater.

[28] Huebsch N., Mooney D. J. (2009). Inspiration and
application in the evolution of biomaterials. Nature.

[29] Cui M., Gong Y., Du M., Wang K., Li T., Zhu X., Wang S., Luo Z. (2021). An antifouling electrochemical biosensor
based on a protein imprinted hydrogel for human immunoglobulin G recognition
in complex biological media. Sens. Actuators,
B.

[30] Narkar A. R., Tong Z., Soman P., Henderson J. H. (2022). Smart biomaterial
platforms: Controlling and being controlled by cells. Biomaterials.

[31] Malik S., Muhammad K., Waheed Y. (2023). Emerging Applications of Nanotechnology
in Healthcare and Medicine. Molecules.

[32] Aditya T., Allain J. P., Jaramillo C., Restrepo A. M. (2022). Surface Modification
of Bacterial Cellulose for Biomedical Applications. Int. J. Mol. Sci..

[33] Wen X., Zheng Y., Wu J., Wang L., Yuan Z., Peng J., Meng H. (2015). Immobilization
of collagen peptide
on dialdehyde bacterial cellulose nanofibers via covalent bonds for
tissue engineering and regeneration. Int. J.
Nanomed..

[34] Mujtaba M., Negi A., King A. W. T., Zare M., Kuncova-Kallio J. (2023). Surface modifications
of nanocellulose for drug delivery applications; a critical review. Curr. Opin. Biomed. Eng.

[35] Cheng C., Yang R., Wang Y., Fu D., Sheng J., Guo X. (2023). A bacterial cellulose-based separator with tunable pore size for
lithium-ion batteries. Carbohydr. Polym.

[36] Samyn P., Meftahi A., Geravand S. A., Heravi M. E., Najarzadeh H., Sabery M. S. K., Barhoum A. (2023). Opportunities for bacterial
nanocellulose
in biomedical applications: Review on biosynthesis, modification and
challenges. Int. J. Biol. Macromol.

[37] Norrrahim M. N. F., Nurazzi N. M., Jenol M. A., Farid M. A. A., Janudin N., Ujang F. A., Yasim-Anuar T. A., Syed Najmuddin S. U., Ilyas R. A. (2021). Emerging development of nanocellulose
as an antimicrobial
material: an overview. Mater. Adv.

[38] Saadi M. A. S. R., Cui Y., Bhakta S. P., Hassan S., Harikrishnan V., Siqueira I. R., Pasquali M., Bennett M., Ajayan P. M., Rahman M. M. (2025). Flow-induced 2D
nanomaterials intercalated aligned
bacterial cellulose. Nat. Commun.

[39] Zhang H., Zhang D., Cai H., Ma Y., Li K., Zhang P., Guo Y. (2025). A bacterial cellulose-based multifunctional
conductive hydrogel for flexible strain sensors and supercapacitors. Carbohydr. Polym.

[40] Yousefpour P., Ni K., Irvine D. J. (2023). Targeted modulation of immune cells and tissues using
engineered biomaterials. Nat. Rev. Bioeng.

[41] Wang F., Cai X., Shen Y., Meng L. (2023). Cell–scaffold interactions
in tissue engineering for oral and craniofacial reconstruction. Bioact. Mater.

[42] Zhang Y., Habibovic P. (2022). Delivering
Mechanical Stimulation to Cells: State of
the Art in Materials and Devices Design. Adv.
Mater.

[43] Wieszczycka K., Staszak K., Woźniak-Budych M. J., Litowczenko J., Maciejewska B. M., Jurga S. (2021). Surface functionalization
–
The way for advanced applications of smart materials. Coord. Chem. Rev.

[44] Yoon S., Fuwad A., Jeong S., Cho H., Jeon T. J., Kim S. M. (2024). Surface Deformation of Biocompatible Materials: Recent
Advances in Biological Applications. Biomimetics.

[45] Liu S., Yu J.-M., Gan Y.-C., Qiu X.-Z., Gao Z.-C., Wang H., Chen S.-X., Xiong Y., Liu G.-H., Lin S.-E. (2023). Biomimetic
natural biomaterials for tissue engineering
and regenerative medicine: new biosynthesis methods, recent advances,
and emerging applications. Military Med. Res.

[46] Reversat A., Gaertner F., Merrin J., Stopp J., Tasciyan S., Aguilera J., De Vries I., Hauschild R., Hons M., Piel M., Callan-Jones A., Voituriez R., Sixt M. (2020). Cellular locomotion using environmental
topography. Nature.

[47] Wang Y., Naleway S. E., Wang B. (2020). Biological
and bioinspired materials:
Structure leading to functional and mechanical performance. Bioact. Mater.

[48] Egan P., Sinko R., LeDuc P., Keten S. (2015). The role of mechanics
in biological and bio-inspired systems. Nat.
Commun.

[49] Hernandez J. L., Woodrow K. A. (2022). Medical Applications
of Porous Biomaterials: Features
of Porosity and Tissue-Specific Implications for Biocompatibility. Adv. Healthcare Mater.

[50] Lazarus B. S., Velasco-Hogan A., Gómez-Del Río T., Meyers M. A., Jasiuk I. (2020). A review of impact resistant biological
and bioinspired materials and structures. J.
Mater. Res. Technol.

[51] Nikolova M. P., Chavali M. S. (2019). Recent advances in biomaterials for
3D scaffolds: A
review. Bioact. Mater.

[52] Mishra R. K., Ha S. K., Verma K., Tiwari S. K. (2018). Recent progress
in selected bio-nanomaterials and their engineering applications:
An overview. J. Sci.: Adv. Mater. Devices.

[53] Liu S., Yang M., Xu W. (2024). Three-Dimensional
Hierarchical Cellulose
Structures Based on Microbial Synthesis and Advanced Biofabrication. Chem. Bio Eng.

[54] Gao K., Wu N., Ji B., Liu J. (2023). A Film Electrode upon Nanoarchitectonics
of Bacterial Cellulose and Conductive Fabric for Forehead Electroencephalogram
Measurement. Sensors.

[55] Horta-Velázquez A., Morales-Narváez E. (2022). Nanocellulose in wearable sensors. Green Anal. Chem.

[56] Brown R. M. (2004). Cellulose
structure and biosynthesis: What is in store for the 21st century?. J. Polym. Sci., Part A: Polym. Chem.

[57] Akintunde M. O., Adebayo-Tayo B. C., Ishola M. M., Zamani A., Horvath I. S. (2022). Bacterial
Cellulose Production from agricultural Residues by two Komagataeibacter
sp. Strains. Bioengineered.

[58] Aytekin A. Ö., DemirbaĞ D. D., Bayrakdar T. (2016). The statistical
optimization of bacterial cellulose production via semi-continuous
operation mode. J. Ind. Eng. Chem.

[59] Rühs P. A., Storz F., López
Gómez Y. A., Haug M., Fischer P. (2018). 3D bacterial cellulose
biofilms formed by foam templating. npj Biofilms
Microbiomes.

[60] Embuscado M. E., BeMiller J. N., Marks J. S. (1996). Isolation
and partial characterization
of cellulose produced by Acetobacter xylinum. Food Hydrocolloids.

[61] Li G., Wang L., Deng Y., Wei Q. (2022). Research progress of
the biosynthetic strains and pathways of bacterial cellulose. J. Ind. Microbiol. Biotechnol.

[62] Naomi R., Bt Hj Idrus R., Fauzi M. B. (2020). Plant- vs. Bacterial-Derived Cellulose
for Wound Healing: A Review. Int. J. Environ.
Res. Public Health.

[63] Ross P., Mayer R., Benziman M. (1991). Cellulose
biosynthesis and function
in bacteria. Microbiol Rev.

[64] Thiruvengadam V., Vitta S. (2013). Ni-bacterial cellulose
nanocomposite; A magnetically active inorganic-organic
hybrid gel. RSC Adv.

[65] Manan S., Ullah M. W., Ul-Islam M., Shi Z., Gauthier M., Yang G. (2022). Bacterial cellulose: Molecular regulation of biosynthesis, supramolecular
assembly, and tailored structural and functional properties. Prog. Mater. Sci.

[66] Römling U., Galperin M. Y. (2015). Bacterial cellulose biosynthesis: diversity of operons,
subunits, products, and functions. Trends Microbiol.

[67] Babi M., Williams A., Reid M., Grandfield K., Bassim N. D., Moran-Mirabal J. M. (2023). Unraveling
the Supramolecular Structure
and Nanoscale Dislocations of Bacterial Cellulose Ribbons Using Correlative
Super-Resolution Light and Electron Microscopy. Biomacromolecules.

[68] Portela R., Leal C. R., Almeida P. L., Sobral R. G. (2019). Bacterial
cellulose:
A versatile biopolymer for wound dressing applications. Microb. Biotechnol.

[69] “Biocompatibility,” Link: Biocompatibility | definition of biocompatibility by Medical dictionary (thefreedictionary.com). The Free Dictionary 2024.

[70] Helenius G., Bäckdahl H., Bodin A., Nannmark U., Gatenholm P., Risberg B. (2006). In vivo biocompatibility of bacterial cellulose. J. Biomed. Mater. Res.

[71] Qui K., Netravali A. (2014). A Review of Fabrication and Applications of Bacterial
Cellulose Based Nanocomposites. Polym. Rev..

[72] Shi Z., Zhang Y., Phillips G. O., Yang G. (2014). Utilization of bacterial
cellulose in food. Food Hydrocolloids.

[73] Cakar F., Katı A., Özer I., Demirbag D. D., Sahin F., Aytekin A. Ö. (2014). Newly developed medium and strategy for bacterial cellulose
production. Biochem. Eng. J.

[74] Hestrin S., Schramm M. (1954). Synthesis of cellulose by Acetobacter
xylinum. II.
Preparation of freeze-dried cells capable of polymerizing glucose
to cellulose. Biochem. J.

[75] Zeng X., Small D. P., Wan W. (2011). Statistical
optimization of culture
conditions for bacterial cellulose production by Acetobacter xylinum
BPR 2001 from maple syrup. Carbohydr. Polym.

[76] Jagannath A., Kalaiselvan A., Manjunatha S. S., Raju P. S., Bawa A. S. (2008). The effect
of pH, sucrose and ammonium sulphate concentrations on the production
of bacterial cellulose (Nata-de-coco) by Acetobacter xylinum. World J. Microbiol. Biotechnol.

[77] Rani M. U., Rastogu N., Appaiah K. A. A. (2011). Statistical
optimization of medium
composition for bacterial cellulose production by Gluconacetobacter
hansenii UAC09 using coffee cherry husk extract–an agro-industry
waste. J. Microbiol. Biotechnol.

[78] Hasanin M. S., Abdelraof M., Hashem A. H., El Saied H. (2023). Sustainable bacterial
cellulose production by Achromobacter using mango peel waste. Microb. Cell Fact.

[79] Soares
da Silva F. A. G., Fernandes M., Souto A. P., Ferreira E. C., Dourado F., Gama M. (2019). Optimization of bacterial nanocellulose
fermentation using recycled paper sludge and development of novel
composites. Appl. Microbiol. Biotechnol.

[80] Du R., Wang Y., Zhao F., Qiao X., Song Q., Li S., Kim R. C., Pan L., Han Y., Xiao H., Zhou Z. (2020). Production Optimization
and Partial Characterization of Bacterial
Cellulose from Gluconacetobacter xylinus TJU-D2. Waste Biomass Valorization.

[81] Saleh A. K., El-Gendi H., Soliman N. A., El-Zawawy W. K., Abdel-Fattah Y. R. (2022). Bioprocess development for bacterial cellulose biosynthesis
by novel Lactiplantibacillus plantarum isolate along with characterization
and antimicrobial assessment of fabricated membrane. Sci. Rep.

[82] Aswini K., Gopal N. O., Uthandi S. (2020). Optimized
culture conditions for
bacterial cellulose production by Acetobacter senegalensis MA1. BMC Biotechnol.

[83] Anusuya R. S., Anandham R., Kumutha K., Gayathry G., Mageshwaran V., Uthandi S. (2020). Characterization and
Optimization of bacterial cellulose
produced by Acetobacter spp. J. Environ. Biol..

[84] Embuscado M. E., Marks J. S., BeMiller J. N. (1994). Bacterial
cellulose II.Optimization
of cellulose production by Acetobacter xylinum through response surface
methodology. Food Hydrocolloids.

[85] Bae S., Shoda M. (2005). Statistical optimization
of culture conditions for bacterial cellulose
production using Box-Behnken design. Biotechnol.
Bioeng.

[86] Rastogi A., Banerjee R. (2020). Statistical optimization
of bacterial cellulose production
by Leifsonia soli and its physico-chemical characterization. Process Biochem.

[87] He F., Yang H., Zeng L., Hu H., Hu C. (2020). Production
and characterization of bacterial cellulose obtained by Gluconacetobacter
xylinus utilizing the by-products from Baijiu production. Bioprocess Biosyst. Eng.

[88] Fernandes I. D. A., Pedro A. C., Ribeiro V. R., Bortolini D. G., Ozaki M. S. C., Maciel G. M., Haminiuk C. W. (2020). Bacterial cellulose:
From production optimization to new applications. Int. J. Biol. Macromol.

[89] Martínez E., Posada L., Botero J. C., Rios-Arango J. A., Zapata-Benabithe Z., López S., Molina-Ramírez C., Osorio M. A., Castro C. I. (2023). Nata de
fique: A cost-effective alternative
for the large-scale production of bacterial nanocellulose. Ind. Crops Prod.

[90] Henry S., Dhital S., Sumer H., Butardo V. (2024). Solid-State Fermentation
of Cereal Waste Improves the Bioavailability and Yield of Bacterial
Cellulose Production by a Novacetimonas sp. Isolate. Foods.

[91] Elfaleh I., Abbassi F., Habibi M., Ahmad F., Guedri M., Nasri M., Garnier C. (2023). A comprehensive review of natural
fibers and their composites: An eco-friendly alternative to conventional
materials. Results Eng.

[92] Serra A., González I., Oliver-Ortega H., Tarrès Q., Delgado-Aguilar M., Mutjé P. (2017). Reducing the
Amount of Catalyst in
TEMPO-Oxidized Cellulose Nanofibers: Effect on Properties and Cost. Polymers.

[93] Felgueiras C., Azoia N. G., Gonçalves C., Gama M., Dourado F. (2021). Trends on
the Cellulose-Based Textiles: Raw Materials and Technologies. Front. Bioeng. Biotechnol.

[94] Brown R. M., Willison J. H., Richardson C. L. (1976). Cellulose
biosynthesis in Acetobacter
xylinum: visualization of the site of synthesis and direct measurement
of the in vivo process. Proc. Natl. Acad. Sci.

[95] Matthysse A. G. (1983). Role of
bacterial cellulose fibrils in Agrobacterium tumefaciens infection. J. Bacteriol.

[96] Wong H. C., Fear A. L., Calhoon R. D., Eichinger G. H., Mayer R., Amikam D., Benziman M., Gelfand D. H., Meade J. H., Emerick A. W. (1990). Genetic organization of the cellulose
synthase operon in Acetobacter xylinum. Proc.
Natl. Acad. Sci. U. S. A.

[97] Acoustic Diaphragm And Its Production https://patents.google.com/patent/JPH06284495A/en. Accessed 11 September 2025.

[98] Bungay, H. R. ; Serafica, G. C. Production of microbial cellulose using a rotating disk film bioreactor, US 5,955,326 A, 1997.

[99] Lloyd A. (2004). Bacterial
cellulose scaffolds for cartilage repair. Materials
Today.

[100] Yoon S. H., Jin H. J., Kook M. C., Pyun Y. R. (2006). Electrically
Conductive Bacterial Cellulose by Incorporation of Carbon Nanotubes. Biomacromolecules.

[101] Nogi M., Yano H. (2008). Transparent Nanocomposites Based
on Cellulose Produced by Bacteria Offer Potential Innovation in the
Electronics Device Industry. Adv. Mater.

[102] Zhu H., Jia S., Yang H., Tang W., Jia Y., Tan Z. (2010). Characterization of
bacteriostatic sausage casing: A composite of
bacterial cellulose embedded with ε-polylysine. Food Sci. Biotechnol.

[103] Lin W. C., Lien C. C., Yeh H. J., Yu C. M., Hsu S. H. (2013). Bacterial cellulose and bacterial cellulose–chitosan
membranes for wound dressing applications. Carbohydr.
Polym.

[104] Fürsatz M., Skog M., Sivlér P., Palm E., Aronsson C., Skallberg A., Greczynski G., Khalaf H., Bengtsson T., Aili D. (2018). Functionalization of bacterial cellulose wound dressings with the
antimicrobial peptide ε -poly-L-Lysine. Biomed. Mater.

[105] Schaffner M., Rühs P. A., Coulter F., Kilcher S., Studart A. R. (2017). 3D printing
of bacteria into functional complex materials. Sci. Adv.

[106] Greca L. G., Lehtonen J., Tardy B. L., Guo J., Rojas O. J. (2018). Biofabrication
of multifunctional nanocellulosic 3D
structures: a facile and customizable route. Mater. Horiz.

[107] Fleury B., Abraham E., De La Cruz J. A., Chandrasekar V. S., Senyuk B., Liu Q., Cherpak V., Park S., Ten Hove J. B., Smalyukh I. I. (2020). Aerogel from Sustainably
Grown Bacterial Cellulose Pellicles as a Thermally Insulative Film
for Building Envelopes. ACS Appl. Mater. Interfaces.

[108] Rühs P. A., Malollari K. G., Binelli M. R., Crockett R., Balkenende D. W. R., Studart A., Messersmith P. B. (2020). Conformal
Bacterial Cellulose Coatings as Lubricious Surfaces. ACS Nano.

[109] Choi S. M., Shin E. J. (2020). The Nanofication and Functionalization
of Bacterial Cellulose and Its Applications. Nanomaterials.

[110] Acharyya P. P., Sarma M., Kashyap A. (2024). Recent advances
in
synthesis and bioengineering of bacterial nanocellulose composite
films for green, active and intelligent food packaging. Cellulose.

[111] Chang Y., Zhou L., Xiao Z., Liang J., Kong D., Li Z., Zhang X., Li X., Zhi L. (2017). Embedding Reduced Graphene Oxide in Bacterial Cellulose-Derived Carbon
Nanofibril Networks for Supercapacitors. ChemElectrochem.

[112] Jiang Y., Yan J., Wu X., Shan D., Zhou Q., Jiang L., Yang D., Fan Z. (2016). Facile synthesis
of carbon nanofibers-bridged porous carbon nanosheets for high-performance
supercapacitors. J. Power Sources.

[113] Lai F., Miao Y. E., Zuo L., Lu H., Huang Y., Liu T. (2016). Biomass-Derived Nitrogen-Doped Carbon
Nanofiber Network: A Facile
Template for Decoration of Ultrathin Nickel-Cobalt Layered Double
Hydroxide Nanosheets as High-Performance Asymmetric Supercapacitor
Electrode. Small.

[114] Ccorahua R., Troncoso O. P., Rodriguez S., Lopez D., Torres F. G. (2017). Hydrazine treatment improves conductivity
of bacterial cellulose/graphene nanocomposites obtained by a novel
processing method. Carbohydr. Polym.

[115] Ma L., Liu R., Niu H., Zhao M., Huang Y. (2016). Flexible and
freestanding electrode based on polypyrrole/graphene/bacterial cellulose
paper for supercapacitor. Compos. Sci. Technol.

[116] Muller D., Rambo C. R., Porto L. M., Schreiner W. H., Barra G. M. O. (2013). Structure and properties of polypyrrole/bacterial
cellulose
nanocomposites. Carbohydr. Polym.

[117] Shao Y., Fan Z., Zhong M., Xu W., He C., Zhang Z. (2021). Polypyrrole/bacterial cellulose nanofiber
composites
for hexavalent chromium removal. Cellulose.

[118] Wang H., Zheng R., He P., Li X., Shi Z., Yang G. (2024). A capacitive polypyrrole-wrapped
carbon cloth/bacterial
cellulose antibacterial dressing with electrical stimulation for infected
wound healing. Adv. Compos. Hybrid Mater.

[119] Fang Q., Zhou X., Deng W., Zheng Z., Liu Z. (2016). Freestanding bacterial cellulose-graphene
oxide composite membranes
with high mechanical strength for selective ion permeation. Sci. Rep.

[120] Luo H., Dong J., Xu X., Wang J., Yang Z., Wan Y. (2018). Exploring excellent
dispersion of graphene nanosheets in three-dimensional
bacterial cellulose for ultra-strong nanocomposite hydrogels. Compos., Part A.

[121] Troncoso O. P., Torres F. G. (2020). Bacterial Cellulose—Graphene
Based Nanocomposites. Int. J. Mol. Sci..

[122] Torres F. G., Ccorahua R., Arroyo J., Troncoso O. P. (2019). Enhanced
conductivity of bacterial cellulose films reinforced with NH_4_I-doped graphene oxide. Polym.-Plast. Technol.
Mater.

[123] Feng Y., Zhang X., Shen Y., Yoshino K., Feng W. (2012). A mechanically
strong, flexible and conductive film based on bacterial
cellulose/graphene nanocomposite. Carbohydr.
Polym.

[124] Parnsubsakul A., Ngoensawat U., Wutikhun T., Sukmanee T., Sapcharoenkun C., Pienpinijtham P., Ekgasit S. (2020). Silver nanoparticle/bacterial
nanocellulose paper composites for paste-and-read SERS detection of
pesticides on fruit surfaces. Carbohydr. Polym.

[125] Wang J., Wang X., Xiong P., Tu J., Yang Z., Yao F., Gama M., Zhang Q., Luo H., Wan Y. (2022). Flexible, robust and washable bacterial cellulose/silver
nanowire conductive paper for high-performance electromagnetic interference
shielding. J. Mater. Chem. A.

[126] Wang W., Khabazian S., Roig-Sanchez S., Laromaine A., Roig A., Tonti D. (2021). Carbons derived from
alcohol-treated bacterial cellulose with optimal porosity for Li–O2
batteries. Renewable Energy.

[127] Ma L., Bi Z., Xue Y., Zhang W., Huang Q., Zhang L., Huang Y. (2022). Bacterial
cellulose: an encouraging
eco-friendly nano-candidate for energy storage and energy conversion. J. Mater. Chem. A.

[128] Tian J., Hao R., Yang C., Ge X., Tang T., Liu Z., Wang J., Cao M., Jiang Y., Lin C. (2023). Fabrication
of a 3D bacterial cellulose
intercalated MoS2@rGO nanocomposite for high performance supercapacitors. New J. Chem.

[129] Wang C., Hu C., Ding Y., Li Z., Wang Z., Lin X., Zhou X., Xu J. (2025). h-CoFe2O4/Ti3C2Tx/BNCHybrid
Aerogels with Modulation Impedance Matching for Electromagnetic Wave
Absorption and Health Monitoring. ACS Appl.
Mater. Interfaces.

[130] Rawat J., Sajwan D., Garimella S., Sharma H., Dwivedi C. (2023). Boron Nitride quantum dots: A rising
star in sensing applications. Nano Trends.

[131] Zhang X., Yao J., Yan Y., Zhang Y., Tang Y., Yang Y. (2024). Bacterial Cellulose
Incorporating
Multicolor Fluorescent Probes for Visual Acidity Detection in Paper-Based
Cultural Relics. ACS Appl. Mater. Interfaces.

[132] Celi I. H., Peña González P. T., Martínez Bonilla C. A. (2023). Bacterial nanocellulose and CdTe
quantum dots: assembled nanopaper for heavy metal detection in aqueous
solution. J. Mater. Chem. C.

[133] Morales-Narváez E., Golmohammadi H., Naghdi T., Yousefi H., Kostiv U., Horák D., Pourreza N., Merkoçi A. (2015). Nanopaper as an Optical Sensing Platform. ACS Nano.

[134] Caligiuri V., Leone F., Favale O., De Santo M., Bruno M. D. L., Mileti O., Pane A., Patra A., Petti L., Guzman-Puyol S. (2024). Micro- and Nano-Structured
Bacteria Growth Media for Planar Bio-Photonics. Adv. Opt. Mater.

[135] Shi Y., Jiao H., Sun J., Lu X., Yu S., Cheng L., Wang Q., Liu H., Biranje S., Wang J., Liu J. (2022). Functionalization of
nanocellulose
applied with biological molecules for biomedical application: A review. Carbohydr. Polym.

[136] Fu J., Su J., Wang P., Yu Y., Wang Q., Cavaco-Paulo A. (2015). Enzymatic
processing of protein-based fibers. Appl. Microbiol.
Biotechnol.

[137] Torres F. G., Troncoso O. P., Gonzales K. N., Sari R. M., Gea S. (2020). Bacterial
cellulose-based biosensors. Med.
Devices Sens.

[138] Wang W., Li H.-Y., Zhang D. W., Jiang J., Cui Y.-R., Qiu S., Zhou Y.-L., Zhang X.-X. (2010). Fabrication
of Bienzymatic Glucose Biosensor Based on Novel Gold Nanoparticles-Bacteria
Cellulose Nanofibers Nanocomposite. Electroanalysis.

[139] Li D., Ao K., Wang Q., Lv P., Wei Q. (2016). Preparation
of Pd/bacterial cellulose hybrid nanofibers for dopamine detection. Molecules.

[140] Li G., Sun K., Li D., Lv P., Wang Q., Huang F., Wei Q. (2016). Biosensor based on
bacterial cellulose-Au
nanoparticles electrode modified with laccase for hydroquinone detection. Colloids Surf., A.

[141] Shishparenok A. N., Furman V. V., Dobryakova N. V., Zhdanov D. D. (2024). Protein Immobilization on Bacterial Cellulose for Biomedical
Application. Polymers.

[142] Kim S., Kang J. Y., Balance W. C., Sutton B. P., Shin D. H., Jang K. H., Shin M., Kong M., Kim J. W. (2021). Fabrication
of cell penetrating peptide-conjugated bacterial cellulose nanofibrils
with remarkable skin adhesion and water retention performance. Int. J. Pharm.

[143] van Zyl E. M., Coburn J. M. (2024). Functionalization of Bacterial Cellulose
with the Antimicrobial Peptide KR-12 via Chimerical Cellulose-Binding
Peptides. Int. J. Mol. Sci..

[144] Munir M., Nosheen S., Muhammad N., Uroos M., Mustafa W., Khan R., Saeed W. S., Wang R., Sharif F. (2024). Ionic liquid
treated bacterial cellulose sheets as
prospective biodegradable implant materials. Cellulose.

[145] Cabo M., More N., Alston J. R., Laws E., Kulkarni R., Mohan R. V., LaJeunesse D. R. (2025). Insight
on the Mechanical Properties of Facile Hydrophobic-Barrier-Patterned
Bacterial Nanocellulose via Self-Bonding Mechanism. ACS Nanosci. Au.

[146] Brown E. E., Laborie M. P. G., Zhang J. (2012). Glutaraldehyde
treatment
of bacterial cellulose/fibrin composites: impact on morphology, tensile
and viscoelastic properties. Cellulose.

[147] Treesuppharat W., Rojanapanthu P., Siangsanoh C., Manuspiya H., Ummartyotin S. (2017). Synthesis
and characterization of
bacterial cellulose and gelatin-based hydrogel composites for drug-delivery
systems. Biotechnol. Rep..

[148] Yang L., Yang Q., Lu D. N. (2014). Effect
of chemical
crosslinking degree on mechanical properties of bacterial cellulose/poly­(vinyl
alcohol) composite membranes. Monatsh. Chem.

[149] Quero F., Nogi M., Lee K. Y., Poel G. V., Bismarck A., Mantalaris A., Yano H., Eichhorn S. (2011). Cross-Linked
Bacterial Cellulose Networks Using Glyoxalization. ACS Appl. Mater. Interfaces.

[150] Sommer A., Dederko-Kantowicz P., Staroszczyk H., Sommer S., Michalec M. (2021). Enzymatic and Chemical
Cross-Linking
of Bacterial Cellulose/Fish Collagen Composites—A Comparative
Study. Int. J. Mol. Sci..

[151] Yang Q., Ma H., Dai Z., Wang J., Dong S., Shen J., Dong J. (2017). Improved thermal and
mechanical properties of bacterial cellulose with the introduction
of collagen. Cellulose.

[152] Liu Y., Liu H., Guo S., Zhao Y., Qi J., Zhang R., Ren J., Cheng H., Zong M., Wu X., Li B. (2024). A review of
carbon nanomaterials/bacterial cellulose
composites for nanomedicine applications. Carbohydr.
Polym.

[153] Si H., Luo H., Xiong G., Yang Z., Raman S. R., Guo R., Wan Y. (2014). One-Step In
Situ Biosynthesis of Graphene Oxide–Bacterial
Cellulose Nanocomposite Hydrogels. Macromol.
Rapid Commun.

[154] Wei J., Wang B., Li Z., Wu Z., Zhang M., Sheng N., Liang Q., Wang H., Chen S. (2020). A 3D-printable
TEMPO-oxidized bacterial cellulose/alginate hydrogel with enhanced
stability via nanoclay incorporation. Carbohydr.
Polym.

[155] Menegasso J. F., Moraes N. A. C., Vásquez T. P., Felipetti F. A., Antonio R. V., Dutra R. C. (2022). Modified montmorillonite-bacterial
cellulose composites as a novel dressing system for pressure injury. Int. J. Biol. Macromol.

[156] De Lima L. F., Ferreira A. L., Ranjan I., Collman R. G., De Araujo W. R., De La Fuente-Nunez C. (2023). A bacterial
cellulose-based and low-cost
electrochemical biosensor for ultrasensitive detection of SARS-CoV-2. Cell Rep. Phys. Sci.

[157] Wu C., Yue Y., Huang B., Ji H., Wu L., Huang H. (2024). CRISPR-powered
microfluidic biosensor for preamplification-free detection
of ochratoxin A. Talanta.

[158] Li J., Lu D., Yang J., You R., Chen J., Weng J., Lu Y. (2023). Three-dimensional flexible
SERS substrate
based on bacterial cellulose membrane for detection of glutathione
in serum. Cellulose.

[159] Zhao R., Zhang T., Qiu X., Cao Z., Gao S., Song X., Li Y., Chen F., Zhou X. (2024). Charge transport
properties and mechanisms of bacterial cellulose (BC)-Zinc complexes. Carbohydr. Polym.

[160] Zhang Y. H., Lei Q. Y., Liu R. J., Zhang L., Lyu B., Liu L. P., Ma J. Z. (2023). Self-healing cellulose-based flexible
sensor: A review. Ind. Crops Prod.

[161] Fernandes A., Cruz-Lopes L., Esteves B., Evtuguin D. (2023). Nanotechnology
Applied to Cellulosic Materials. Materials.

[162] Pan X., Li J., Ma N., Ma X., Gao M. (2023). Bacterial
cellulose hydrogel for sensors. Chem. Eng. J.

[163] Bondancia T. J., Soares A. C., Popolin-Neto M., Gomes N. O., Raymundo-Pereira P., Barud H. S., Machado S. A. S., Ribeiro S. J. L., Melendez M. E., Carvalho A. L., Reis R. M., Paulovich F. V., Oliveira O. N. (2022). Low-cost bacterial
nanocellulose-based interdigitated biosensor to detect the p53 cancer
biomarker. Biomater. Adv.

[164] Song J., Fan M., Zhang R., Qu M., Tang P., Wang H., Bin Y. (2024). Highly sensitive humidity
sensor based on composite film of partially reduced graphene oxide
and bacterial cellulose. Biosens. Bioelectron.

[165] Farooq U., Ullah M. W., Yang Q., Aziz A., Xu J., Zhou L., Wang S. (2020). High-density
phage particles immobilization
in surface-modified bacterial cellulose for ultra-sensitive and selective
electrochemical detection of Staphylococcus aureus. Biosens. Bioelectron.

[166] Wu Y., Nie Q., Zeng X., Wei P., Zhu C., You R., Lin C., Lin L. (2024). Bacterial cellulose membrane SERS
substrate for in-situ reduction of gold nanoparticles for detecting
TNF-α. Microchem. J.

[167] Jia X., Zhang M., Zhang Y., Fu Y., Sheng N., Chen S., Wang H., Du Y. (2024). Enhanced Selective
Ion Transport in Highly Charged Bacterial Cellulose/Boron Nitride
Composite Membranes for Thermo-Osmotic Energy Harvesting. Nano Lett.

[168] Zamani S., Ghanbari K., Bonyadi S. (2023). Electrochemical determination
of metformin via a carbon paste electrode modified with an Ag NPs/Cu
2 O/CuO-decorated bacterial nanocellulose composite. Anal. Methods.

[169] Phumma R., Phamonpon W., Rodthongkum N., Ummartyotin S. (2024). Fabrication
of Silver Nanoparticle Loaded into Nanocellulose
Derived from Hemp and Poly­(vinyl alcohol)-Based Composite as an Electrode
for Electrochemical Sensors for Lactate Determination. ACS Omega.

[170] Ghasemi S., Bari M. R., Pirsa S., Amiri S. (2020). Use of bacterial
cellulose film modified by polypyrrole/TiO2-Ag nanocomposite for detecting
and measuring the growth of pathogenic bacteria. Carbohydr. Polym.

[171] Rebelo A., Liu Y., Liu C., Schäfer K. H., Saumer M., Yang G. (2019). Poly­(4-vinylaniline)/polyaniline
bilayer functionalized bacterial cellulose membranes as bioelectronics
interfaces. Carbohydr. Polym.

[172] Subhedar A., Bhadauria S., Ahankari S., Kargarzadeh H. (2021). Nanocellulose
in biomedical and biosensing applications: A review. Int. J. Biol. Macromol.

[173] Zhang T., Wang W., Zhang D., Zhang X.-X., Ma Y., Zhou Y., Qi L. (2010). Biotemplated
Synthesis of Gold Nanoparticle–Bacteria
Cellulose Nanofiber Nanocomposites and Their Application in Biosensing. Adv. Funct. Mater.

[174] You R., Wang H., Wang C., Huang J., Zhu H., Liu Y., Zhang J. H., Liu J., Yu X., Lu Y. (2023). Bacterial
cellulose loaded with silver nanoparticles as a flexible, stable and
sensitive SERS-active substrate for detection of the shellfish toxin
DTX-1. Food Chem.

[175] Lormaneenopparat P., Yukird J., Rodthongkum N., Hoven V. P. (2023). Bacterial cellulose composite hydrogel for pre-concentration
and mass spectrometric detection of thiol-containing biomarker. Int. J. Biol. Macromol.

[176] Chen L., Lou J., Rong X., Liu Z., Ding Q., Li X., Jiang X., Ji X., Han W. (2023). Super-stretching and
high-performance ionic thermoelectric hydrogels
based on carboxylated bacterial cellulose coordination for self-powered
sensors. Carbohydr. Polym.

[177] Woods G. A., Rommelfanger N. J., Hong G. (2020). Bioinspired materials
for in vivo bioelectronic neural interfaces. Matter.

[178] Riva E. R., Micera S. (2021). Progress and challenges of implantable
neural interfaces based on nature-derived materials. Bioelectron. Med.

[179] Yang J., Du M., Wang L., Li S., Wang G., Yang X., Zhang L., Fang Y., Zheng W., Yang G., Jiang X. (2018). Bacterial Cellulose
as a Supersoft Neural Interfacing Substrate. ACS Appl. Mater. Interfaces.

[180] Jiji, S. ; Maharajan, K. ; Kadirvelu, K. Nanocellulose Materials. In Recent developments of bacterial nanocellulose porous scaffolds in biomedical applications. Oraon, R. ; Rawtani, D. ; Singh, P. ; Hussain, C. M. Eds.; Elsevier, 2022 pp. 83–104.

[181] Vomero M., Ciarpella F., Zucchini E., Kirsch M., Fadiga L., Stieglitz T., Asplund M. (2022). On the longevity of
flexible neural interfaces: Establishing biostability of polyimide-based
intracortical implants. Biomaterials.

[182] Chen C., Zhang T., Zhang Q., Feng Z., Zhu C., Yu Y., Li K., Zhao M., Yang J., Liu J., Sun D. (2015). Three-Dimensional
BC/PEDOT Composite Nanofibers with
High Performance for Electrode–Cell Interface. ACS Appl. Mater. Interfaces.

[183] Chen C., Zhang T., Zhang Q., Chen X., Zhu C., Xu Y., Yang J., Liu J., Sun D. (2016). Biointerface
by Cell Growth on Graphene Oxide Doped Bacterial Cellulose/Poly­(3,4-ethylenedioxythiophene)
Nanofibers. ACS Appl. Mater. Interfaces.

[184] Meng S., Zhang Y., Wu N., Peng C., Huang Z., Lin Z., Qi C., Liu Z., Kong T. (2023). Ultrasoft, sensitive fiber-like sensor by assembly
of bacterial cellulose
(BC) nanofibrils and BC molecules for biocompatible strain sensing. Nano Res.

[185] Pértile R., Moreira S., Andrade F., Domingues L., Gama M. (2012). Bacterial cellulose modified using
recombinant proteins to improve
neuronal and mesenchymal cell adhesion. Biotechnol.
Prog.

[186] Guo R., Li J., Chen C., Xiao M., Liao M., Hu Y., Liu Y., Li D., Zou J., Sun D., Torre V., Zhang Q., Chai R., Tang M. (2021). Biomimetic
3D bacterial cellulose-graphene foam hybrid scaffold regulates neural
stem cell proliferation and differentiation. Colloids Surf., B.

[187] Reynolds J., Wilkins M., Martin D., Taggart M., Rivera K. R., Tunc-Ozdemir M., Rufty T., Lobaton E., Bozkurt A., Daniele M. A. (2024). Evaluating Bacterial Nanocellulose
Interfaces for Recording Surface Biopotentials from Plants. Sensors.

[188] Wilkins M. D., Rivera K. R., Martin D., Bozkurt A. Y., Lobaton E. J., Daniele M. A. (2019). Characterization
of Screen-Printed
Bioimpedance Electrodes on Nanocellulose Substrate. IEEE Sensors.

[189] Anton-Sales I., Roig-Sanchez S., Traeger K., Weis C., Laromaine A., Turon P., Roig A. (2021). In vivosoft tissue
reinforcement with bacterial nanocellulose. Biomater. Sci.

[190] Osorio M., Ortiz I., Gañán P., Naranjo T., Zuluaga R., Van Kooten T., Castro C. (2019). Novel surface modification of three-dimensional
bacterial
nanocellulose with cell-derived adhesion proteins for soft tissue
engineering. Mater. Sci. Eng.

[191] Zhou T., Qiao Z., Yang M., Wu K., Xin N., Xiao J., Liu X., Wu C., Wei D., Sun J., Fan H. (2023). Hydrogen-bonding topological remodeling
modulated ultra-fine
bacterial cellulose nanofibril-reinforced hydrogels for sustainable
bioelectronics. Biosens. Bioelectron.

[192] Panaitescu D. M., Lupescu I., Frone A. N., Chiulan I., Nicolae C. A., Tofan V., Stefaniu A., Somoghi R., Trusca R. (2017). Medium Chain-Length Polyhydroxyalkanoate
Copolymer
Modified by Bacterial Cellulose for Medical Devices. Biomacromolecules.

[193] An H., Zhang M., Gu Z., Jiao X., Ma Y., Huang Z., Wen Y., Dong Y., Zhang P. (2024). Advances in
Polysaccharides for Cartilage Tissue Engineering Repair: A Review. Biomacromolecules.

[194] Mira-Cuenca C., Meslier T., Roig-Sanchez S., Laromaine A., Roig A. (2021). Patterning Bacterial Cellulose Films
with Iron Oxide Nanoparticles and Magnetic Resonance Imaging Monitoring. ACS Appl. Polym. Mater.

[195] Standard Test Method for Evaluation of MR Image Artifacts from Passive Implants. https://img.antpedia.com/standard/files/pdfs_ora/20240528/ASTM/F2119-24.pdf. Accessed 6 September 2025.

[196] Klemm D., Kramer F., Moritz S., Lindström T., Ankerfors M., Gray D., Dorris A. (2011). Nanocelluloses: A new
family of nature-based materials. Angew. Chem.
Int. Ed.

[197] Wan M. M., Sun X. D., Li Y. Y., Zhou J., Wang Y., Zhu J. H. (2016). Facilely fabricating multifunctional
N-enriched carbon. ACS Appl. Mater. Interfaces.

[198] Jacek P., Dourado F., Gama M., Bielecki S. (2019). Molecular
aspects of bacterial nanocellulose biosynthesis. Microb. Biotechnol.

[199] Jozala A. F., de Lencastre-Novaes L.
C., Lopes A. M., de Carvalho Santos-Ebinuma V., Mazzola P. G., Pessoa A., Grotto D., Gerenutti M., Chaud M. V. (2016). Bacterial nanocellulose production and application:
a 10-year overview. Appl. Microbiol. Biotechnol.

[200] Taokaew S. (2024). Bacterial Nanocellulose Produced
by Cost-Effective
and Sustainable Methods and Its Applications: A Review. Fermentation.

[201] Dourado, F. ; Fontão, A. ; Leal, M. ; Cristina Rodrigues, A. ; Gama, M. Process Modeling and Techno-Economic Evaluation of an Industrial Bacterial NanoCellulose Fermentation Process Bacterial Nanocellulose: from Biotechnology To Bio-Economy Gama, M. ; Dourado, F. ; Bielecki, S. Elsevier 2017 199–214

[202] Behera B., Laavanya D., Balasubramanian P. (2022). Techno-economic
feasibility assessment of bacterial cellulose biofilm production during
the Kombucha fermentation process. Bioresour.
Technol.

[203] Absharina D., Putra F. J., Ogino C., Kocsubé S., Veres C., Vágvölgyi C. (2025). Bacterial
Cellulose
Production in Co-Culture Systems: Opportunities, Challenges, and Future
Directions. Appl. Microbiol.

[204] Da Cruz T., Las-Casas B., Dias I. K., Arantes V. (2024). Nanocelluloses
as sustainable emerging technologies: State of the art and future
challenges based on life cycle assessment. Sustainable
Mater. Technol.

[205] Silva F., Branco S., Dourado F., Neto B., Gama M. (2025). Life cycle assessment of bacterial cellulose and comparison to other
cellulosic sources. J. Cleaner Prod.

[206] Pogorelova N., Rogachev E., Digel I., Chernigova S., Nardin D. (2020). Bacterial cellulose nanocomposites:
morphology and
mechanical properties. Materials.

[207] Torgbo S., Sukyai P. (2020). Biodegradation and
thermal stability
of bacterial cellulose as biomaterial: The relevance in biomedical
applications. Polym. Degrad. Stab.

[208] Melro L. A., Alves C., Fernandes M., Rocha S., Mehravani B., Ribeiro A. I., Azevedo S., Cardoso V. F., Carvalho Ó., Dourado N. (2025). S Bacterial
nanocellulose as a versatile scaffold for biomedical applications:
Synthesis, functionalization, and future prospects. Appl. Mater. Today.

[209] Kaczmarek M., Białkowska A. M. (2025). Enzymatic
functionalization of bacterial
nanocellulose: current approaches and future prospects. J. Nanobiotechnol.

[210] Xiao L., Feng S., Hua M. Z., Lu X. (2023). Rapid determination
of thiram on apple using a flexible bacterial cellulose-based SERS
substrate. Talanta.

[211] Rostamabadi H., Bist Y., Kumar Y., Yildirim-Yalcin M., Ceyhan T., Falsafi S. R. (2024). Cellulose nanofibers, nanocrystals,
and bacterial nanocellulose: Fabrication, characterization, and their
most recent applications. Future Postharvest
Food.

[212] Araujo J. A., Taxeidis G., Pereira E. H., Azeem M., Pantelic B., Jeremic S., Ponjavic M., Chen Y., Mojicevic M., Nikodinovic-Runic J., Topakas E., Brennan
Fournet M. (2024). Biotechnological model for ubiquitous mixed petroleum-
and bio-based plastics degradation and upcycling into bacterial nanocellulose. J. Cleaner Prod.

[213] Lin L., Chen L., Lu C., Chen G., Hong F. F. (2024). Chitosan
particles embedded bacterial nanocellulose flat membrane for hemodialysis. Int. J. Biol. Macromol.

[214] Núñez D., Oyarzún P., González S., Martínez I. (2024). Toward biomanufacturing
of next-generation bacterial
nanocellulose (BNC)-based materials with tailored properties: A review
on genetic engineering approaches. Biotechnol.
Adv.

[215] Kanugo, A. ; Chaudhari, P. ; Gautam, R. K. Biomedical Applications of Cellulose In Biopolymers For Biomedical Applications Wiley 2024 87–128.

[216] Chithra Lekha P., Marini L., Jhajharia S. K., Aadinath W., Muthuvijayan V., Paramasivan M., Gonmei M C., Padmanabhan M. K., Jeyaraman M., Mahajan R. L. (2024). Edge-functionalized coal-derived graphene oxide in
bacterial nanocellulose hydrogel for active wound healing. Int. J. Biol. Macromol.

[217] Lamboni L., Li Y., Liu J., Yang G. (2016). Silk Sericin-Functionalized
Bacterial Cellulose as a Potential Wound-Healing Biomaterial. Biomacromolecules.

[218] Buer Boyetey M.-J., Torgbo S., Sukyai P. (2023). Bio-scaffold
for bone
tissue engineering with focus on bacterial cellulose, biological materials
for hydroxyapatite synthesis and growth factors. Eur. Polym. J.

[219] Market Data Forecast: “Global Nanocellulose Market Size. https://www.marketdataforecast.com/market-reports/nanocellulose-market. 2025.

[220] Abeer M.
M., Mohd Amin M. C. I., Martin C. (2014). A review of bacterial
cellulose-based drug delivery systems: their biochemistry, current
approaches and future prospects. J. Pharm. Pharmacol.

[221] Walling B., Bharali P., Giridharan B., Gogoi B., Sorhie V., Alemtoshi, ManI S. K. (2023). Bacterial nanocellulose:
A novel nanostructured bio-adsorbent for green remediation technology. Acta Ecol. Sin..

[222] Sahari N. S., Shahir S., Ibrahim Z., Hasmoni S. H., Altowayti W. A. H. (2023). Bacterial nanocellulose and its application
in heavy
metals and dyes removal: a review. Environ.
Sci. Pollut. Res.

[223] Lee S., Abraham A., Lim A. C. S., Choi O., Seo J. G., Sang B. I. (2021). Characterisation of bacterial nanocellulose
and nanostructured
carbon produced from crude glycerol by Komagataeibacter sucrofermentans. Bioresour. Technol.

[224] Biranje S. S., Kaushik S., Marewad D., Yadav A., Vankundre V., Panse M., Joshi I., Goli A., Shahid M., Kulkarni K. (2024). Applications of nanocellulose
and its derivatives in developing sustainable textiles. Cellulose.

[225] Science.gov “Bacterial Cellulose” and “Bacterial Nanocellulose” Science.gov. 2025.

